# Burdens and risk factors for mortality in adolescents and young adults aged 10–24 years in Australia and comparison with OECD countries between 2000 and 2021: Global Burden of Disease Study 2021

**DOI:** 10.1136/bmjph-2025-002986

**Published:** 2025-11-28

**Authors:** Zahra Ali Padhani, Jodie C Avery, Salima Meherali, Gizachew A Tessema, Zohra S Lassi

**Affiliations:** 1 School of Public Health, Faculty of Health and Medical Sciences, The University of Adelaide, Adelaide, South Australia, Australia; 2 Robinson Research Institute, The University of Adelaide, Adelaide, South Australia, Australia; 3 College of Health Sciences, Faculty of Nursing, University of Alberta, Edmonton Clinic Health Academy, Edmonton, Alberta, Canada; 4 Curtin University School of Population Health, Curtin University, Perth, Western Australia, Australia

**Keywords:** Epidemiology, Adolescent, Public Health

## Abstract

**Introduction:**

Adolescents are considered the healthiest population of all age groups, yet they are vulnerable to illnesses and deaths from preventable causes. We aimed to investigate the burden of morbidity, mortality and risk factors for mortality among adolescents and young adults (10–24 years) in Australia compared with the Organisation for Economic Co-operation and Development (OECD) countries.

**Methods:**

We accessed the Global Burden of Disease (GBD) Study 2021 data for OECD countries, including Australia, between 2000 and 2021. Country and age-specific estimates with 95% uncertainty intervals were obtained from the GBD Compare and Results Tool for all-cause mortality and disability-adjusted life years (DALYs). The top 15 level 3 risk factors for mortality for adolescents and young adults were investigated, with rates reported per 100 000 population. Annualised rates of change (ARoC) in mortality and DALY rates were estimated using a log-linear model to quantify temporal trends between distinct time periods. Causes of death by age groups, sex and year for other diseases were measured using the Cause of Death Ensemble model.

**Results:**

Between 2000 and 2021, mortality rates in adolescents and young adults decreased in Australia and across OECD regions, with substantial reductions (47.1 in 2000 vs 27.9 in 2021) in Australia. DALYs decreased slightly (11 850.2 in 2000 vs 10 363.9 in 2021), ranking Australia eighth among OECD countries in 2021. The ARoC in Australia showed a decline in the rate of mortality and DALYs by 2.48% and 0.64%, respectively. Overall, OECD countries experienced a slower decline, with an ARoC of 0.92% for mortality and 0.04% for DALYs from 2000-2021. Young adults (20–24 years) had higher mortality and DALYs than other age groups. In 2021, males in Australia and OECD countries had higher mortality rates, while Australian females had more DALYs than males. Alcohol and drug use were the leading risk factors for death.

**Conclusion:**

Adolescent and young adult mortality in Australia has declined more than the OECD average, with minimal reduction in disease burden. Disparities in mortality rates and morbidity burden continue to grow across countries, age groups and genders due to the limited focus on males and young adults. This study calls for comprehensive health strategies that address these disparities to reduce the disease burden in young adults, specifically among males.

**WHAT IS ALREADY KNOWN ON THIS TOPIC**

- Existing research using Global Burden of Disease (GBD) study data (2013–2019) has explored global mortality, injuries and disease burden in adolescents and young adults.
- Prior studies focus on broad global trends or specific diseases rather than providing a comprehensive analysis for Organisation for Economic Co-operation and Development (OECD) countries, including Australia.
- Some research has reported on disease burden in Australia, but none has specifically analysed trends among adolescents and young adults in this context.

**WHAT THIS STUDY ADDS**

- This study utilised the estimates from the GBD Study 2021, reporting on mortality and disease burden among adolescents and young adults residing in OECD countries.
- It reports on mortality rates and disease burden among adolescents and young adults by age group, gender and country.
- It identifies the top 15 risk factors contributing to higher mortality rates, suggesting opportunities for improvement.

**HOW THIS STUDY MIGHT AFFECT RESEARCH, PRACTICE AND POLICY**

- Understanding adolescent and young adult morbidity and mortality trends is essential to guide investments and policies.
- Attention needs to be paid to the unique risk factors among adolescents and young adults and across different genders, specifically males.
- There is a need for evidence-based policies to scale up high-quality healthcare, school-based programmes and investment in specific areas of healthcare, especially for young adults and males residing in OECD countries, including Australia.

## Introduction

 Adolescence is a key phase of transition in a person’s life, characterised by profound physical, biological, intellectual and cognitive changes with the acquisition of new social roles and responsibilities.^1 2^ Despite being considered the healthiest population of all age groups, adolescents are vulnerable to illnesses, injuries and deaths from largely preventable causes.^3^ This vulnerability is partly driven by their engagement with risky behaviours related to diet, physical activity, substance use and sexual activity.^3^ The Global Burden of Disease (GBD) study reported that in 2019 alone, approximately 1.49 million adolescents and young adults aged 10–24 years died, with the majority of deaths attributed to injuries, interpersonal violence, maternal and nutritional conditions, and communicable and non-communicable diseases (NCDs).^4^

Adolescents and young adults were disproportionately affected by the COVID-19 pandemic and its aftermath, including the ensuing economic austerity measures and global financial crisis.^5^ Despite widespread recognition of the challenges faced by this age group, their health and well-being continue to be under-represented in national policies in both high-income and low-income and middle-income countries.^5^ Recent global initiatives, such as the Global Accelerated Action for the Health of Adolescents framework,^6^ 2030 Every Woman Every Child Global Strategy for Women’s, Children’s and Adolescents’ Health,^7^ ^8^ have highlighted the importance of adolescent health. However, progress in improving the health outcomes of young adults has been slow, with stark health inequities persisting particularly among Indigenous and low-socioeconomic youth.^9^

According to the World Bank’s 2023 report,^10^ adolescence is a critical period for the development of risk factors for NCDs, such as overweight and obesity, which are particularly prevalent in Latin America and the Caribbean. In countries like the Bahamas and Chile, median body mass index (BMI) exceeds the Organization for Economic Co-operation and Development (OECD) averages. While deaths due to interpersonal violence continue to claim lives, especially among young males in Venezuela, El Salvador and Brazil.^10^

Australia’s performance in adolescent health, while better than some OECD countries, remains mixed. The Australian Research Alliance of Children and Youth 2018 Report Card^11^ noted that while Australia shows progress in reducing smoking rates among youth, mental health issues, food insecurity, bullying and declining immunisation coverage are growing concerns. Premature death and disability in Australian adolescents and young people carry significant social and economic costs, with 38 per 100 000 deaths reported among young people (aged 15–24 years) in 2021.^1^ Given these challenges and limited research on adolescents and young adults, a systematic evaluation of disease burden, associated risk factors and trends over time is crucial to inform public health interventions and policy responses.^12^ Understanding how diseases and risk factors evolve will be instrumental in shaping national health policies and healthcare delivery for adolescents and young adults.

The growing burden of disease has negative consequences on the healthcare system and economic stability, making it imperative to understand and monitor disease trends and their associated risk factors.^13^ Currently, the Australian Institute of Health and Welfare (AIHW)^14^ provides comprehensive national-level data on mortality and disease burden, but there remains a critical gap in understanding these patterns, specifically among adolescents and young adults. Given that OECD nations share similar socioeconomic profiles and healthcare capabilities, comparing Australia’s disease burden metrics with these countries can identify best practices and inform evidence-based policy decisions. Therefore, this study aims to systematically analyse the burden of disease and risk factors for mortality among adolescents and young adults in Australia from 2000 to 2021 and compare it with OECD countries.

## Methods

### Overview

The GBD Study 2021 consists of annual assessments of 459 health outcomes and risk factors from 204 countries and territories and 983 locations.^15^ Since the last release of the GBD Study 2019, there has been a significant improvement in the methodology of cause-of-death estimation. The GBD Study 2021 has incorporated advanced methods to account for uncertainty in cause-of-death data to improve the estimation of less common causes of death. Moreover, the study has included 199 new sets of vital registration cause-of-death data from various countries and has also added new data sources, including surveillance data, verbal autopsy data and other data types, to improve the accuracy and completeness of cause-of-death estimates.^16^ The GBD 2021 Results tool^17^ estimates were provided for the number of deaths and the mortality rate per 100 000 population with uncertainty intervals (UI) by sex and age group, including for adolescents and young adults aged 10–24 years. All-cause mortality estimates were also provided for each OECD country. A detailed description of the metrics, data sources and statistical modelling for mortality and disability-adjusted life years (DALY) estimates at various geographical levels and causes of death and risk factors has been reported previously.^1618^,^20^

### Data sources

Our analysis used data from the GBD Study 2021 estimates to explore the burden and risk factors for mortality among adolescents and young adults (10–24 years) in OECD countries. We included data from 38 OECD member countries, which include Australia, Austria, Belgium, Canada, Chile, Colombia, Costa Rica, Czech Republic, Denmark, Estonia, Finland, France, Germany, Greece, Hungary, Iceland, Ireland, Israel, Italy, Japan, Korea, Latvia, Lithuania, Luxembourg, Mexico, Netherlands, New Zealand, Norway, Poland, Portugal, Slovak Republic, Slovenia, Spain, Sweden, Switzerland, Turkey, United Kingdom and USA. We obtained our data from the GBD Compare and Results Tool where we extracted the country-specific estimates on all-cause mortality and ‘DALYs’ as an indicator of disability. Data sources for the GBD study 2021 were methodically compiled, adhering to the Guidelines for Accurate and Transparent Health Estimates Reporting (GATHER) approach.^21^ This ensures that each step from data collection to visualisation is rigorously documented in a dedicated portal.

### Data analysis

We estimated the burden and trends of mortality and morbidity in adolescents and young adults aged 10–24 years in OECD countries, including Australia, between 2000 and 2021. Mortality burden was measured as mortality rate per 100 000 population, while morbidity was assessed using DALYs rate per 100 000 population. DALYs were calculated as the sum of Years of Life Lost (YLLs) and Years Lived with Disability (YLDs).^21^ YLLs are estimated as the number of deaths per 100 000 people multiplied by a global standard life expectancy at the age of death, while YLDs are estimated as the combined prevalence and duration of a disease or injury weighted by a measure of disease severity that ranges from 0 (full health) to 1 (fatal severity).^21^ As the sum of YLLs and YLDs, one DALY represents the loss of 1 year of healthy life in the population.^21^ For both mortality and DALY rates, the 95% UIs were derived for mortality and DALY rate estimates, with the 2.5th and 97.5th percentiles drawn as the lower and upper bounds. Details regarding the estimation of DALYs, YLLs and YLDs, including methods to assess the relative morbidity and mortality from individual diseases and injuries, as well as the disability weights, have been published elsewhere.^19 21^

Annualised rates of change (ARoC) in mortality and DALY rates were also estimated using a log-linear model to quantify temporal trends between distinct time periods. The ARoC was calculated as the difference in the natural logarithm of mortality rates at two time points, divided by the number of years between them, and multiplied by 100 to express the average percentage change per year. This approach is consistent with the log-linear trend model applied in the GBD studies,^22 23^ where mortality and DALY rates are modelled on a logarithmic scale over time.

In this study, we presented data on the number and rates of mortality and DALYs by country, gender and age. Based on the physical, cognitive, social and emotional development during adolescence and young adulthood, we followed the Sawyer’s classification and grouped age as young adolescents (early adolescence): 10–14 years; older adolescents (late adolescence): 15–19 years; young adults (young adulthood): 20–24 years; and adolescents and young adults (adolescence and young adulthood): 10–24 years.^24^ Causes of death by age groups, sex and year for other diseases were measured using the Cause of Death Ensemble model (CODEm). A detailed description of CODEm is reported elsewhere.^16^ We also investigated the top 15 risk factors of death for adolescents and young adults.

GBD classifies causes and risk factors at four main levels.^25^ *Level* 1 risk factors comprised of behavioural and metabolic risks, environmental and occupational risk factors, which are further disaggregated at *level 2* into 20 risk factors, including high BMI, child and maternal malnutrition and air pollution. *Level* 3 included more specific risk factors such as child growth failure and fine particulate matter (ie, airborne particles/pollutants with an aerodynamic diameter less than 2.5 µm (PM2.5) contributing to air pollution). At this level, nine risks at *level 2* were further broken down into 42 additional risk factors in addition to 11 risk factors, which were not further disaggregated. *Level 4* is a more nuanced classification that includes 70 discrete category risk factors. A full list of risk factors at all levels has been published elsewhere.^25^ In this study, we present estimates by *level 3* risk factors.

Similarly, the GBD 2021 cause of death consists of four levels of causes of diseases, injuries and deaths comprising both fatal and non-fatal causes. *Level* 1 causes include three broad aggregated categories of Injuries, NCDs and communicable, maternal, neonatal and nutritional diseases. *Level* 2 further disaggregated these categories into 22 clusters of causes, which were further categorised at *level* 3 and *level 4*. GBD study 2021, included 12 new causes of death, including COVID-19 and pandemic related mortalities, pulmonary arterial hypertension, and nine different types of cancers.^26^ A complete list of causes at all levels has been published elsewhere.^26^

### Patient and public involvement

Patients or members of the public were not involved in this study, nor will they be involved in the dissemination of the results.

## Results

### Mortality among adolescents and young adults

There were an estimated 1861 (95% UI: 1845 to 1879) adolescent and young adult deaths in Australia in 2000 and 1320 (95% UI 1304 to 1336) deaths in 2021. In OECD countries, 149 436 (95% UI 1 47 967 to 1 50 978) adolescent and young adult deaths were estimated in 2000 and 118 671 (95% UI 1 16 667 to 1 20 890) deaths in 2021 (table 1). The rate of adolescent and young adult mortality in Australia was 47.1 (95% UI 46.6 to 47.5) per 100 000 population in 2000, which declined to 27.9 (95% UI 27.6 to 28.3) per 100 000 population in 2021 (table 2). Similarly, a reduction in the rate of adolescent and young adult mortality was observed in OECD countries, with 56.6 deaths (95% UI 56.1 to 57.2) per 100 000 population in 2000 and 46.7 deaths (95% UI 45.9 to 47.6) per 100 000 population in 2021. From 2000 to 2021, the mortality rate in Australia declined at an average rate of 2.48% per year (95% UI −2.59% to −2.38%), while between 2000–2010 and 2010–2021, the mortality rate in Australia declined at an average rate of 3.76% (95% UI −3.94% to −3.58%) and 1.32% (95% UI −1.51% to −1.13%), respectively (table 2). In OECD countries, the mortality rate declined at an average rate of 0.92% (95% UI −1.05% to −0.78%) per year from 2000 to 2021. In 2000–2010, the mortality rate in OECD countries declined at an average rate of 1.90% (95% UI −2.11% to −1.69%), while there was a negligible decline (0.03%) in the mortality rate in OECD countries between 2010 and 2021 (95% UI −0.29% to 0.24%).

**Table 1:** Number of deaths and DALYs among adolescents and young adults aged 10–24 years from 2000 to 2021

Country	Mortality N (95% UI)	DALYs N (95% UI)		
	2000	2010	2021	2000	2010	2021
*All OECD countries average*	149 435.6 (147 967.1 to 150 977.6)	122 137.1 (120 814.3 to 123 502.6)	118 670.8 (116 666.7 to 120 890.3)	30 112 863.6 (24 819 012.9 to 36 336 249.4)	2 818 5743.3 (22 863 253.9 to 34 349 517.6)	28 764 291.7 (23 235 353.3 to 35401484.5)
Australia	1861.4 (1844.6 to 1879.3)	1394.6 (1382 to 1406.9)	1320.2 (1303.8 to 1336.3)	468 799.2 (381 388.6 to 569 778.6)	467 007.7 (368 821.2 to 576 082.1)	489 663.6 (38 4849.8 to 606 613.9)
Austria	665.6 (658.6 to 673.1)	512 (506.1 to 518)	339.4 (332.4 to 346.6)	154 893.8 (125 309.1 to 189 541.3)	149 020.1 (117 827.3 to 186 329.1)	137 431.2 (104 689.2 to 174 658.1)
Belgium	934 (925.1 to 942.3)	614.1 (607.5 to 620.4)	451.5 (440.4 to 463.1)	209 706.6 (169 931 to 258 862.4)	195 537.8 (153 329 to 249 664.9)	188 154.9 (143 788.5 to 240 601.5)
Canada	2658.5 (2639.7 to 2676.4)	2313.2 (2298 to 2328.8)	2310.2 (2263.4 to 2354.9)	640 617.6 (515 839.7 to 785 122.1)	647 151.8 (513 735.3 to 796 750.5)	666 964.5 (523 356.5 to 822 323.3)
Chile	1956.9 (1941.6 to 1973.3)	2079.3 (2058.9 to 2098.4)	1574.6 (1555.1 to 1593.9)	427 663.8 (352 731.1 to 5 15 856)	460 849.8 (374 635.5 to 553 568.1)	424 733 (336 416.8 to 520 344.5)
Colombia	19 241.2 (18 809.3 to 19 747.2)	12 849.9 (12 584.6 to 13 092.4)	10 413.6 (9338.6 to 11 556.9)	2 177 522.3 (1 954 330.2 to 2 440 110.3)	1 747 021.8 (1 512 607.8 to 2 029 381)	1 577 822.8 (1 337 430.7 to 1 858 130.2)
Costa Rica	638.9 (625.1 to 651.2)	712.8 (699.2 to 728.5)	703.3 (683.6 to 722.3)	124 650.1 (102 899.9 to 151 289.3)	136 080.1 (112 417.6 to 1 64 447)	127 493.2 (105 824.8 to 154 236.9)
Czechia	1075 (1060.3 to 1091.6)	654.8 (643.7 to 666.2)	390.3 (381.6 to 399.6)	220 395.4 (180 746.6 to 267 732.6)	161 290.5 (128 735.6 to 197 298.6)	132 063.3 (103 786.7 to 166 252.4)
Denmark	348.8 (344.3 to 353.1)	232.7 (228.8 to 236.7)	198 (193.4 to 202.9)	95 068.1 (75 660.6 to 117 689.6)	94 198.5 (72 530 to 118 425.8)	96 910.8 (74 902.2 to 123 998.3)
Estonia	253.5 (247.8 to 259.5)	128.5 (125.1 to 132.1)	75.8 (73.1 to 78.6)	38 149.7 (32 545.3 to 44 609.4)	25 240.6 (20 757.2 to 30 337.3)	19 180.2 (15 540 to 23 527)
Finland	459 (452.4 to 465.3)	378.2 (371.7 to 384.7)	302 (293.5 to 310.7)	108 000.6 (87 470.5 to 134 144.7)	101 763.6 (81 275.9 to 125 835.9)	94 139.7 (74 401 to 1 17 775)
France	5182.9 (5156.9 to 5210.8)	3506.7 (3488.9 to 3526.8)	2595.8 (2574.5 to 2619)	1 294 619.2 (1 042 273.7 to 1 609 802.8)	1 175 140 (915 822.3 to 1 493 298.8)	1 183 197.7 (900 140.2 to 1 506 821.4)
Germany	5527.4 (5502.6 to 5552)	3401 (3381.7 to 3419.9)	2544 (2520.5 to 2567)	1 507 278.6 (1 198 648.2 to 1 891 615.7)	1 324 500.7 (1 018 453.7 to 1 697 868.4)	1 238 940.2 (947 341.4 to 1 596 738.7)
Greece	1067.5 (1057.2 to 1078.6)	668.2 (661 to 675.4)	398.7 (386.8 to 409.4)	257 677.5 (206 833.9 to 319 486.1)	191 854.6 (151 774.2 to 239 368.1)	157 725.8 (120 546.4 to 1 99 132)
Hungary	941.5 (931.8 to 951.5)	555.5 (548.6 to 562)	445.3 (438.6 to 452.7)	211 336.2 (172 306.4 to 257 705.9)	154 437.2 (123 011.7 to 190 978.2)	129 465.9 (102 443.9 to 161 055.3)
Iceland	27.7 (26.6 to 28.7)	19.3 (18.4 to 20.2)	16.3 (15.4 to 17.4)	6879 (5531.5 to 8512.7)	6677.9 (5199.2 to 8427)	6465.9 (5007.2 to 8237.8)
Ireland	497.8 (490.6 to 504.6)	311.1 (306.5 to 315.5)	159.3 (154.8 to 164.1)	112 443.4 (91 380.1 to 138 593.7)	97 599.3 (76 441.3 to 122 117.7)	95 805.8 (72 927.6 to 122 613.4)
Israel	686.3 (678.3 to 693.3)	494.1 (488 to 500.3)	585.8 (576.4 to 596)	167 617.8 (134 372.4 to 207 328.1)	168 570.4 (131 370.4 to 213 250.5)	205 679.4 (160 016 to 2 60 467)
Italy	3885.6 (3866.1 to 3905.4)	2342.3 (2328.2 to 2355.4)	1560.9 (1548.1 to 1575.2)	1 018 449.9 (814 368.4 to 1 271 198.6)	853 676.3 (663 110.3 to 1 088 915.2)	812 631 (617 627.6 to 1 055 075.9)
Japan	7345 (7310.9 to 7376.9)	4855.4 (4833.1 to 4877.5)	3829.2 (3801.4 to 3854.3)	2 004 890.5 (1 618 064.5 to 2 467 804.8)	1 553 137.1 (1 239 270.1 to 1 931 774.3)	1 418 906.1 (1 117 950.5 to 1 778 280.9)
Latvia	451.7 (444.4 to 459.4)	226.1 (221.2 to 230.8)	104.3 (101.6 to 107)	64 685.7 (55 887.4 to 75 333.6)	40 856.4 (34 131.3 to 48 848.5)	24 970.5 (20 075 to 30 726.2)
Lithuania	674.4 (664.6 to 684.4)	440.2 (432.6 to 448.4)	168.7 (165 to 172.3)	96 741.9 (83 329.4 to 1 13 008)	70 743.6 (59 809.6 to 83 049.3)	40 885.5 (32 826.2 to 50 037.2)
Luxembourg	30.8 (29.8 to 31.9)	22.4 (21.3 to 23.3)	21.4 (20 to 22.8)	8121.8 (6492.7 to 10 115.3)	8479.6 (6608.4 to 10 788.4)	10 101.7 (7722.9 to 12 964.1)
Mexico	21 975 (21 618.7 to 22 349.6)	26 541.2 (26 024.9 to 27 048.9)	28 987.9 (28 445.8 to 29 568.6)	3 628 255.4 (3 069 589.1 to 4 328 434.2)	4 081 614 (3 513 370.8 to 4 804 600.8)	4 328 160.9 (3 704 325.6 to 5 104 502.5)
Netherlands	945.7 (936.4 to 954.9)	635.2 (627.9 to 642.2)	588.8 (579.2 to 598.6)	275 119.6 (217 514.1 to 341 665.7)	268 824 (207 159.2 to 343 016.8)	282595.6 (214704.1 to 364147.9)
New Zealand	462.7 (456.8 to 469.2)	426.1 (420.3 to 432.1)	341.4 (335.8 to 346.8)	104 019.2 (85 218.2 to 125 348.5)	109 453.4 (88 636.9 to 132 740.7)	110 009.1 (87 215 to 136 757.8)
Norway	376.3 (371.4 to 381.7)	317 (312.4 to 321.6)	233.6 (229 to 238.5)	90 133.2 (72 429.2 to 110 177.6)	96 044.6 (75 267.9 to 120 598.9)	94 588.6 (73 000.5 to 120 501.7)
Poland	4748.9 (4716.9 to 4779.6)	3352.5 (3329.8 to 3374.9)	2039.1 (2023.1 to 2054.6)	919 506.5 (760 007.8 to 1 113 556.6)	696 291.9 (569 295.3 to 850 293.2)	521 545.8 (416 649 to 648 565.5)
Portugal	1353.4 (1340.1 to 1365.4)	531.2 (524.7 to 537.3)	355.6 (347.5 to 363.7)	278095.1 (227336.7 to 335765.8)	184211.5 (143628.1 to 233647.4)	168969.4 (129337.5 to 214277.5)
Republic of Korea	4836.5 (4812 to 4860.9)	2867.6 (2848.9 to 2886.6)	1725.2 (1675.2 to 1779.1)	1 054 125.7 (868 133 to 1 285 070.5)	837 125.9 (670 544.3 to 1 034 798.8)	622 211.3 (484 908.9 to 783 154.3)
Slovakia	612.3 (602.1 to 623.1)	412.9 (406.7 to 419.8)	259.2 (253.8 to 264.1)	130 918.2 (106 894.5 to 158 471.8)	97 958.2 (78 880.6 to 119 964.5)	74 254.6 (59 018.3 to 92 255)
Slovenia	216.4 (211.1 to 221.8)	105.7 (102.7 to 108.9)	61.3 (58.4 to 64.3)	42 697.1 (35 127.6 to 51 450.3)	29 502.4 (23 445.1 to 36 641.9)	23 785.8 (18 573.6 to 29 934.7)
Spain	3523.5 (3500.1 to 3546.8)	1598.9 (1587.8 to 1609.7)	1301.8 (1286.6 to 1317.2)	864 007.1 (691 066.3 to 1 078 360.7)	655 522.3 (504 994.6 to 842 594.1)	648 709.8 (496 262.3 to 834 187.9)
Sweden	494 (488.5 to 499)	499.9 (494.5 to 506)	394.5 (363.6 to 422.3)	151 552.8 (119 620.4 to 190 633.6)	165 935.6 (129 747.2 to 209 840.8)	164 510.2 (127 272.6 to 209 548.4)
Switzerland	513.4 (507 to 519.5)	325.2 (320.9 to 329.7)	250.6 (245.2 to 256.5)	145 113.9 (115 135.3 to 178 631.3)	136 571.8 (105 512.6 to 1 72 800)	129 092.1 (97 987.2 to 164 527.6)
Türkiye	13 311.1 (12 086.4 to 14 663.8)	9361.9 (8623.8 to 10 162.8)	8450.6 (7283.6 to 9714.8)	2 423 627.7 (2 030 652 to 2 901 704.6)	2 010 229.6 (1 647 513 to 2 426 749)	2 039 445.6 (1 626 878.8 to 2 508 313.7)
United Kingdom	4112.4 (4076.1 to 4142.8)	3367 (3348.5 to 3385.4)	3025.3 (3000.6 to 3047.6)	1 170 075.6 (926 670.8 to 1 462 783.3)	1 202 455.6 (937 310 to 1 519 352.5)	1 216 910.3 (934 985.6 to 1542 964)
USA	35 542.7 (35 453.5 to 35 636.3)	33 082.6 (32 999.8 to 33 177.3)	40 147.2 (39 664.8 to 40 643.9)	741 9407.5 (6 102 649.1 to 8 932 045.1)	7 783 167.2 (6 274 259.5 to 9 465 524.5)	9 060 169.7 (7 311 285.3 to 10 948 301.3)

Source: https://vizhub.healthdata.org/gbd-results/

DALYs, disability-adjusted life years; OECD, Organisation for Economic Co-operation and Development; UI, uncertainty intervals; USA, The United States of America.

**Table 2:** Mortality rate and annual rate of change in mortality among adolescents and young adults aged 10–24 years from 2000 to 2021

Country	Mortality Rate per 100 000 population (95% UI)			ARoC % (95% UI)		
	2000	2010	2021	2000–2010	2010–2021	2000–2021
*All OECD countries average*	*56.6 (56.1 to 57.2*)	*46.8 (46.3 to 47.4*)	*46.7 (45.9 to 47.6*)	−1.90 (−2.11 to 1.69)	−0.03 (−0.29 to 0.24)	−0.92 (−1.05 to –0.78)
Australia	47.1 (46.6 to 47.5)	32.3 (32.0 to 32.6)	27.9 (27.6 to 28.3)	−3.76 (−3.94 to –3.58)	−1.32 (−1.51 to –1.13)	−2.48 (−2.59 to –2.38)
Austria	46.9 (46.4 to 47.4)	35.2 (34.8 to 35.6)	24.0 (23.5 to 24.5)	−2.87 (−3.10 to –2.65)	−3.47 (−3.76 to –3.17)	−3.18 (−3.34 to –3.03)
Belgium	50.4 (49.9 to 50.8)	31.9 (31.6 to 32.3)	23.0 (22.4 to 23.6)	−4.55 (−4.75 to –4.35)	−2.99 (−3.31 to –2.66)	−3.74 (−3.90 to –3.57)
Canada	43.3 (43.0 to 43.6)	36.0 (35.7 to 36.2)	35.7 (35.0–36.4)	−1.85 (−1.98 to –1.71)	−0.06 (−0.30 to 0.18)	−0.91 (−1.04 to –0.79)
Chile	50.3 (49.9 to 50.7)	49.9 (49.4 to 50.3)	40.1 (39.6 to 40.6)	−0.08 (−0.26 to 0.09)	−1.98 (−2.18 to –1.78)	−1.07 (−1.17 to –0.98)
Colombia	166.0 (162.2 to 170.3)	102.4 (100.3 to 104.4)	87.0 (78.0 to 96.6)	−4.83 (−5.29 to –4.41)	−1.48 (−2.64 to –0.35)	−3.07 (−3.72 to –2.47)
Costa Rica	53.4 (52.3 to 54.4)	57.0 (55.9 to 58.2)	64.3 (62.5 to 66.0)	0.65 (0.26 to 1.08)	1.10 (0.65 to 1.52)	0.88 (0.66 to 1.12)
Czechia	49.0 (48.4 to 49.8)	38.3 (37.7 to 39.0)	25.0 (24.4 to 25.6)	−2.47 (−2.79 to –2.16)	−3.88 (−4.24 to –3.51)	−3.21 (−3.39 to –3.03)
Denmark	37.9 (37.4 to 38.4)	22.8 (22.4 to 23.2)	18.9 (18.4 to 19.3)	−5.10 (−5.39 to –4.80)	−1.71 (−2.08 to –1.34)	−3.33 (−3.50 to –3.15)
Estonia	83.3 (81.4 to 85.2)	53.3 (51.9 to 54.8)	37.5 (36.2 to 38.8)	−4.46 (−4.96 to –3.95)	−3.20 (−3.79 to –2.63)	−3.80 (−4.08 to –3.52)
Finland	46.9 (46.3 to 47.6)	39.5 (38.8 to 40.2)	32.9 (32.0 to 33.9)	−1.72 (−2.04 to –1.41)	−1.66 (−2.07 to –1.24)	−1.69 (−1.89 to –1.49)
France	43.9 (43.7 to 44.2)	29.7 (29.6 to 29.9)	21.2 (21.1 to 21.4)	−3.92 (−4.02 to –3.81)	−3.05 (−3.18 to –2.92)	−3.46 (−3.53 to –3.40)
Germany	39.4 (39.2 to 39.6)	25.9 (25.8 to 26.1)	20.2 (20.0 to 20.3)	−4.18 (-4.28 to –4.08)	−2.29 (−2.42 to –2.16)	−3.19 (−3.25 to –3.13)
Greece	47.8 (47.3 to 48.3)	37.5 (37.1 to 37.9)	25.8 (25.1 to 26.5)	−2.43 (−2.64 to –2.23)	−3.38 (−3.76 to –3.04)	−2.93 (−3.12 to –2.76)
Hungary	43.7 (43.3 to 44.2)	32.1 (31.7 to 32.4)	30.7 (30.2 to 31.2)	−3.10 (−3.33 to –2.88)	−0.41 (−0.65 to –0.14)	−1.69 (−1.82 to –1.56)
Iceland	43.4 (41.8 to 45.1)	28.1 (26.8 to 29.4)	23.9 (22.5 to 25.5)	−4.36 (−5.21 to –3.50)	−1.46 (−2.43 to –0.43)	−2.84 (−3.30 to –2.35)
Ireland	52.1 (51.3 to 52.8)	34.5 (34.0 to 35.0)	16.2 (15.7 to 16.7)	−4.12 (−4.40 to –3.83)	−6.87 (−7.27 to –6.47)	−5.56 (−5.76 to –5.35)
Israel	40.8 (40.3 to 41.2)	26.3 (25.9 to 26.6)	26.0 (25.6 to 26.4)	−4.40 (−4.63 to –4.16)	−0.10 (−0.36 to 0.17)	−2.15 (−2.27 to –2.01)
Italy	41.4 (41.2 to 41.6)	26.4 (26.2 to 26.5)	17.9 (17.8 to 18.1)	−4.50 (−4.62 to –4.40)	−3.51 (−3.63 to –3.36)	−3.98 (−4.04 to –3.91)
Japan	31.9 (31.8 to 32.1)	25.6 (25.4 to 25.7)	21.8 (21.6 to 21.9)	−2.22 (−2.31 to –2.13)	−1.45 (−1.56 to –1.35)	−1.82 (−1.87 to –1.76)
Latvia	85.8 (84.4 to 87.3)	57.7 (56.4 to 58.9)	38.4 (37.4 to 39.4)	−3.98 (−4.37 to –3.61)	−3.68 (−4.12 to –3.26)	−3.82 (−4.03 to –3.63)
Lithuania	86.6 (85.4 to 87.9)	70.5 (69.3 to 71.8)	40.9 (40.0 to 41.8)	−2.06 (−2.39 to –1.73)	−4.95 (−5.32 to –4.60)	−3.58 (−3.75 to –3.41)
Luxembourg	40.6 (39.2 to 42.1)	24.8 (23.7 to 25.9)	20.0 (18.7 to 21.3)	−4.93 (−5.76 to –4.16)	−1.96 (−2.96 to –0.94)	−3.38 (−3.86 to –2.90)
Mexico	69.7 (68.6 to 70.9)	81.0 (79.5 to 82.6)	87.4 (85.7 to 89.1)	1.50 (1.13 to 1.85)	0.68 (0.34 to 1.04)	1.07 (0.90 to 1.24)
Netherlands	33.2 (32.9 to 33.5)	21.0 (20.8 to 21.3)	19.6 (19.2 to 19.9)	−4.55 (−4.77 to –4.35)	−0.67 (−0.92 to –0.41)	−2.52 (−2.64 to –2.39)
New Zealand	56.0 (55.3 to 56.8)	45.5 (44.9 to 46.2)	33.7 (33.2 to 34.3)	−2.07 (−2.34 to –1.80)	−2.73 (−3.01 to –2.47)	−2.42 (−2.56 to –2.28)
Norway	45.5 (44.9 to 46.2)	33.6 (33.1 to 34.1)	23.7 (23.2 to 24.2)	−3.04 (−3.33 to –2.77)	−3.19 (−3.50 to –2.86)	−3.12 (−3.28 to –2.96)
Poland	50.4 (50.1 to 50.8)	45.5 (45.2 to 45.8)	35.0 (34.8 to 35.3)	−1.02 (−1.16 to –0.89)	−2.38 (−2.51 to –2.25)	−1.73 (−1.80 to –1.67)
Portugal	62.1 (61.5 to 62.7)	30.0 (29.6 to 30.3)	21.7 (21.2 to 22.2)	−7.28 (−7.49 to –7.07)	−2.95 (−3.26 to –2.64)	−5.01 (−5.17 to –4.86)
Republic of Korea	44.6 (44.4 to 44.9)	28.7 (28.5 to 28.9)	22.4 (21.7 to 23.1)	−4.42 (−4.53 to –4.30)	−2.26 (−2.59 to –1.92)	−3.29 (−3.45 to –3.12)
Slovakia	46.1 (45.3 to 46.9)	39.0 (38.4 to 39.7)	31.0 (30.4 to 31.6)	−1.67 (−1.99 to –1.33)	−2.09 (−2.43 to –1.78)	−1.89 (−2.07 to –1.72)
Slovenia	51.8 (50.6 to 53.1)	32.1 (31.1 to 33.0)	20.7 (19.8 to 21.7)	−4.81 (−5.34 to –4.27)	−3.97 (−4.67 to –3.27)	−4.37 (−4.71 to –4.02)
Spain	42.8 (42.6 to 43.1)	22.4 (22.2 to 22.6)	18.6 (18.4 to 18.8)	−6.48 (−6.62 to –6.35)	−1.69 (−1.85 to –1.52)	−3.97 (−4.06 to –3.88)
Sweden	30.6 (30.3 to 30.9)	28.8 (28.5 to 29.1)	22.2 (20.5 to 23.8)	−0.61 (−0.83 to –0.38)	−2.35 (−3.20 to –1.63)	−1.52 (−1.96 to –1.15)
Switzerland	40.0 (39.5 to 40.5)	23.7 (23.4 to 24.0)	18.4 (18.0 to 18.9)	−5.23 (−5.49 to –4.98)	−2.29 (−2.61 to –1.96)	−3.69 (−3.85 to –3.52)
Turkey	65.3 (59.3 to 71.9)	48.5 (44.7 to 52.6)	43.9 (37.8 to 50.5)	−2.97 (−4.76 to –1.18)	−0.90 (−3.00 to 1.11)	−1.89 (−3.06 to –0.76)
United Kingdom	37.2 (36.9 to 37.5)	28.1 (27.9 to 28.2)	25.0 (24.8 to 25.2)	−2.81 (−2.95 to –2.67)	−1.06 (−1.18 to –0.94)	−1.89 (−1.97 to –1.82)
USA	59.2 (59.0 to 59.4)	51.1 (51.0 to 51.2)	62.0 (61.2 to 62.7)	−1.48 (−1.53 to –1.42)	1.75 (1.62 to 1.89)	0.22 (0.15 to 0.29)

Source: https://vizhub.healthdata.org/gbd-results/.

ARoC, annualised rate of change, expressed as a percentage change per year in the rate per 100 000 population over the period 2000–2021; OECD, Organisation for Economic Co-operation and Development; UI, uncertainty intervals; USA, The United States of America.

**Figure 1:**
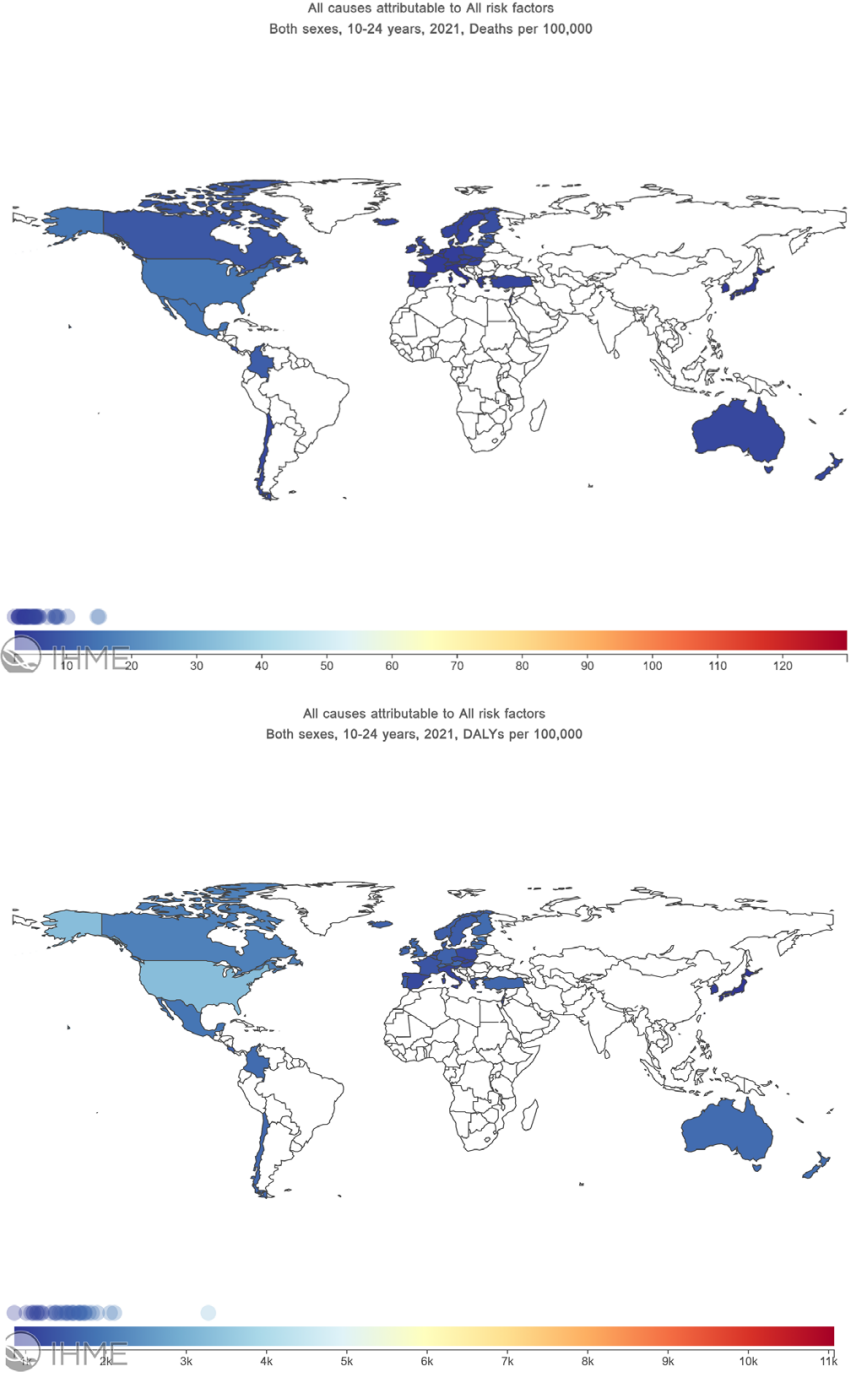
Global map showing deaths and DALYs among adolescents and young adults aged 10–24 years in OECD countries by all causes attributable—all risk factors. DALYs, disability-adjusted life years; OECD, Organisation for Economic Co-operation and Development.

Despite the overall declines, the highest adolescent and young adult mortality rates in 2021 were observed in Mexico (87.4 deaths per 100 000 population), Colombia (87.0 deaths per 100 000 population) and Costa Rica (64.3 deaths per 100 000 population). However, Ireland (16.2 deaths per 100 000 population) and Italy (17.9 deaths per 100 000 population) had the lowest burden of deaths among adolescents and young adults in 2021 (figure 1). Australia (27.9 deaths per 100 000 population) was ranked 16th out of 38 OECD countries in 2021 (table 2).

Stratified for age, 138 (95% UI 132 to 145) deaths were estimated among young adolescents (10–14 years), 448 (95% UI 436 to 460) deaths among older adolescents (15–19 years), and 734 (95% UI 731 to 738) deaths among young adults (20–24 years) residing in Australia in 2021 (online supplemental Table S1). In OECD countries, 13 120 (95% UI 12 623 to 13 693) deaths were estimated among young adolescents, 38 461 (95% UI 37 523 to 39 497) deaths among older adolescents, and 67 090 (95% UI 66 191 to 68 001) deaths were estimated among young adults. In Australia, the rate of deaths was higher among young adults (20–24 years; 45.5 deaths per 100 000 population; 95% UI 45.3 to 45.8) followed by older adolescents (15–19 years; 30.1 deaths per 100 000 population; 95% UI 29.3 to 30.9) and younger adolescents (10–14 years: 8.5 deaths per 100 000 population; 95% UI 8.1 to 8.9) (online supplemental Table S2). Similar trends were observed in OECD countries, with a high rate of deaths (77.2 deaths per 100 000 population; 95% UI 76.2 to 78.3) among young adults (20–24 years) compared with older adolescents (15–19 years; 46.0 deaths per 100 000 population; 95% UI 44.9 to 47.3) and younger adolescents (10–14 years; 15.7 deaths per 100 000 population; 95% UI 15.1 to 16.3). In 2000, the rate of deaths among young adults (20–24 years) was also estimated to be higher in Australia (76.6 deaths per 100 000 population; 95% UI 76.0 to 77.3) and OECD countries on average (84.2 deaths per 100 000 population; 95% UI 83.5 to 85.0), compared with other age groups (ie, adolescents aged 10–19 years).

By gender, 928 deaths (95% UI 915–940) were reported in 2021 among male adolescents and young adults residing in Australia compared with 393 deaths (95% UI 387 to 398) among females (online supplemental Table S3) In OECD countries; 85 358 deaths (95% UI 83 691 to 87 247) were estimated in 2021 among male adolescents and young adults and 33 313 deaths (95% UI 32 774 to 33 853) among adolescent and young adult females. The rate of death among male and female adolescents and young adults residing in Australia in 2021 was 38.3 deaths per 100 000 population (95% UI 37.8 38.8) and 17.1 deaths per 100 000 population (95% UI 16.8 to 17.3), respectively (online supplemental Table S4). The rate of death among male and female adolescents and young adults in OECD countries in 2021 was 65.6 deaths per 100 000 population (95% UI 64.3 to 67.1) and 26.8 per 100 000 population (95% UI 26.4 to 27.3), respectively. The rate of death among adolescent and young adult males (66.1 deaths per 100 000 population; 95% UI 65.5 to 66.8) was also estimated to be higher compared with females (27.2 deaths per 100 000 population; 95% UI 26.9 to 27.5) residing in Australia in 2000. In OECD countries, the rate of death among adolescent and young adult males (80.8 deaths per 100 000 population; 95% UI 80.0 to 81.8) was also estimated to be higher compared with females (31.5 deaths per 100 000 population; 95% UI 31.2 to 31.8) in 2000.

### DALYs among adolescent and young adults

The all-cause number of DALYs among Australian adolescents and young adults in the year 2000 was 468 799.2 (95% UI 381 388.6 to 569 778.6) and 489 663.6 (95% UI 384 849.8 to 606 613.9) in 2021 (table 1). The all-cause number of DALYs among adolescents and young adults in OECD countries in 2000 and 2021 was 30 112 863.6 (95% UI 24 819 012.9 to 36 336 249.4) and 28 764 291.7 (95% UI 23 235 353.3 to 35 401 484.5), respectively. The all-cause rate of DALYs among adolescents and young adults in 2000 in Australia was 11 850.2 DALYs (95% UI 9640.7 to 14 402.8) per 100 000 population, which declined to 10 363.9 DALYs (95% UI 8145.5 to 12 839.2) per 100 000 population in 2021 (table 3). While in the OECD countries, the rate of DALYs among adolescents and young adults in 2000 was 11 408.7 DALYs (95% UI 9403.0 to 13 766.5) per 100 000 population, which declined to 11 313.0 DALYs (95% UI 9138.5 to 13 923.4) per 100 000 population in 2021. From 2000 to 2021, the rate of DALYs in Australia has declined at an average rate of 0.64% per year (95% UI −2.71% to 1.36%). Between 2000–2010 and 2010–2021, the rate of DALYs in Australia has slightly declined at an average rate of 0.91% (95% UI −5.22% to 3.25%) and 0.39% per year (95% UI −4.49 to 3.70%), respectively. In OECD countries, the rate of DALYs declined at a negligible average rate of 0.04% per year (95% UI −1.95% to 1.87%) from 2000 to 2021. Between 2000 and 2010, the rate of DALYs in OECD countries declined at an average rate of 0.54% per year (95% UI −4.51% to 3.37%), while between 2010 and 2021, there was an increase of 0.41% per year (95% UI −3.32% to 4.20%) (table 3).

**Table 3:** Rate of DALYs and the annual rate of change in DALYs among adolescents and young adults aged 10–24 years from 2000 to 2021

Country	Disability-adjusted life-years (DALYs) Rate per 100 000 population (95% UI)			ARoC % (95% UI)		
	2000	2010	2021	2000–2010	2010–2021	2000–2021
*All OECD countries average*	*11 408.7 (9403.0* to 13 766.5)	*10 808.9 (8767.8* to 13 172.6)	*11 313.0 (9138.5* to 13 923.4)	−0.54 (−4.51to 3.37)	0.41 (−3.32 to 4.20)	−0.04 (−1.95 to 1.87)
Australia	11 850.2 (9640.7 to 14 402.8)	10 819.2 (8544.5 to 13 346.2)	10 363.9 (8145.5 to 12 839.2)	−0.91 (−5.22 to 3.25)	−0.39 (−4.49 to 3.70)	−0.64 (−2.71 to 1.36)
Austria	10 908.1 (8824.7 to 13 348.1)	10 236.3 (8093.6 to 12 799.1)	9727.4 (7409.9 to 12 362.3)	−0.64 (−5.00 to 3.72)	−0.46 (−4.97 to 3.85)	−0.55 (−2.80 to 1.61)
Belgium	11 307.6 (9162.9 to 13 958.2)	10 169.6 (7974.4 to 12 984.6)	9578.1 (7319.6 to 12 248.0)	−1.06 (−5.60 to 3.49)	−0.54 (−5.21 to 3.90)	−0.79 (−3.07 to 1.38)
Canada	10 425.8 (8395.1 to 12 777.5)	10 060.5 (7986.4 to 12 386.1)	10 316.3 (8095.1 to 12 719.3)	−0.36 (−4.70 to 3.89)	0.23 (−3.87 to 4.23)	−0.05 (−2.17 to 1.98)
Chile	10 987.7 (9062.5 to 13 253.5)	11 056.7 (8988.2 to 13 281.2)	10 823.0 (8572.6 to 13 259.4)	0.06 (−3.88 to 3.82)	−0.19 (−3.98 to 3.53)	−0.07 (−2.07 to 1.81)
Colombia	18 780.8 (16 855.8 to 21 045.6)	13 926.5 (12 057.8 to 16 177.3)	13 183.3 (11 174.7 to 15 525.4)	−2.99 (−5.57, to 0.41)	−0.50 (−3.36 to 2.30)	−1.69 (−3.01, to 0.39)
Costa Rica	10 421.3 (8602.9 to 12 648.5)	10 875.5 (8984.4 to 13 142.6)	11 658.1 (9676.7 to 14 103.5)	0.43 (−3.42 to 4.24)	0.63 (−2.78 to 4.10)	0.53 (−1.28 to 2.35)
Czechia	10 050.3 (8242.3 to 12 209.0)	9434.9 (7530.6 to 11 541.3)	8456.1 (6645.5 to 10 645.2)	−0.63 (−4.83 to 3.37)	−1.00 (−5.02 to 3.15)	−0.82 (−2.90 to 1.22)
Denmark	10 336.2 (8226.2 to 12 795.7)	9216.7 (7096.6 to 11 587.1)	9228.5 (7132.7 to 11 808.0)	−1.15 (−5.90 to 3.43)	0.01 (−4.41 to 4.63)	−0.54 (−2.78 to 1.72)
Estonia	12 527.9 (10 687.5 to 14 649.2)	10 473.1 (8612.8 to 12 587.9)	9484.4 (7684.3 to 11 633.8)	−1.79 (−5.31 to 1.64)	−0.90 (−4.49 to 2.73)	−1.33 (−3.07 to 0.40)
Finland	11 048.0 (8947.8 to 13 722.4)	10 632.7 (8492.1 to 13 147.9)	10 265.9 (8113.4 to 12 843.4)	−0.38 (−4.80 to 3.85)	−0.32 (−4.39 to 3.76)	−0.35 (−2.50 to 1.72)
France	10 977.7 (8837.9 to 13 650.2)	9956.1 (7759.1 to 12 651.7)	9682.8 (7366.4 to 12 331.3)	−0.98 (−5.65 to 3.59)	−0.25 (−4.92 to 4.21)	−0.60 (−2.94 to 1.59)
Germany	10 737.8 (8539.2 to 13 475.9)	10 094.7 (7762.2 to 12 940.4)	9813.8 (7504.0 to 12 648.0)	−0.62 (−5.52 to 4.16)	−0.26 (−4.95 to 4.44)	−0.43 (−2.79 to 1.87)
Greece	11 534.1 (9258.2 to 14 300.7)	10 756.4 (8509.2 to 13 420.2)	10 216.6 (7808.3 to 12 898.7)	−0.70 (−5.19 to 3.71)	−0.47 (−4.92 to 3.78)	−0.58 (−2.88 to 1.58)
Hungary	9816.4 (8003.5 to 11 970.3)	8914.5 (7100.6 to 11 023.8)	8913.4 (7053.0 to 11 088.3)	−0.96 (−5.22 to 3.20)	0.00 (−4.06 to 4.05)	−0.46 (−2.52 to 1.55)
Iceland	10 788.8 (8675.4 to 13 351.1)	9723.5 (7570.5 to 12 270.3)	9483.2 (7343.8 to 12 081.9)	−1.04 (−5.67 to 3.47)	−0.23 (−4.67 to 4.25)	−0.61 (−2.85 to 1.58)
Ireland	11 762.9 (9559.4 to 14 498.5)	10 829.0 (8481.4 to 13 549.4)	9743.7 (7417.0 to 12 470.2)	−0.83 (−5.36 to 3.49)	−0.96 (−5.48 to 3.50)	−0.90 (−3.19 to 1.27)
Israel	9963.9 (7987.6 to 12 324.4)	8962.3 (6984.5 to 11 337.8)	9126.4 (7100.2 to 11 557.4)	−1.06 (−5.68 to 3.50)	0.16 (−4.25 to 4.58)	−0.42 (−2.63 to 1.76)
Italy	10 849.6 (8675.5 to 13 542.1)	9615.6 (7469.1 to 12 265.3)	9342.1 (7100.3 to 12 129.3)	−1.21 (−5.95 to 3.46)	−0.26 (−4.97 to 4.41)	−0.71 (−3.07 to 1.60)
Japan	8712.3 (7031.3 to 10 723.8)	8176.8 (6524.4 to 10 170.2)	8077.2 (6364.0 to 10 122.9)	−0.63 (−4.97 to 3.69)	−0.11 (−4.26 to 3.99)	−0.36 (−2.48 to 1.74)
Latvia	12 291.3 (10 619.4 to 14 314.5)	10 420.8 (8705.5 to 12 459.2)	9204.8 (7400.2 to 11 326.5)	−1.65 (−4.97 to 1.60)	−1.13 (−4.74 to 2.39)	−1.38 (−3.14 to 0.31)
Lithuania	12 427.7 (10 704.7 to 14 517.3)	11 325.1 (9574.7 to 13 295.0)	9907.5 (7954.6 to 12 125.2)	−0.93 (−4.16 to 2.17)	−1.22 (−4.67 to 2.15)	−1.08 (−2.86 to 0.59)
Luxembourg	10 702.7 (8555.9 to 13 329.5)	9402.8 (7327.9 to 11 963.0)	9441.4 (7218.1 to 12 116.8)	−1.29 (−5.98 to 3.35)	0.04 (−4.59 to 4.57)	−0.60 (−2.92 to 1.66)
Mexico	11 514.1 (9741.2 to 13 736.0)	12 460.8 (10 726.0 to 14 668.0)	13 043.8 (11 163.7 to 15 383.4)	0.79 (−2.47 to 4.09)	0.42 (−2.48 to 3.28)	0.59 (−0.99 to 2.18)
Netherlands	9656.3 (7634.5 to 11 992.0)	8908.0 (6864.7 to 11 366.6)	9389.5 (7133.7 to 12 099.1)	−0.81 (−5.58 to 3.98)	0.48 (−4.23 to 5.15)	−0.13 (−2.47 to 2.19)
New Zealand	12 590.5 (10 314.8 to 15 172.2)	11 700.7 (9475.4 to 14 190.1)	10 865.5 (8614.1 to 13 507.4)	−0.73 (−4.71 to 3.19)	−0.67 (−4.54 to 3.22)	−0.70 (−2.70 to 1.28)
Norway	10 905.9 (8763.7 to 13 331.2)	10 175.0 (7973.9 to 12 776.3)	9578.7 (7392.5 to 12 202.8)	−0.69 (−5.14 to 3.77)	−0.55 (−4.97 to 3.87)	−0.62 (−2.81 to 1.58)
Poland	9763.5 (8069.9 to 11 823.9)	9453.6 (7729.4 to 11 544.5)	8961.5 (7159.1 to 11 144.0)	−0.32 (−4.25 to 3.58)	−0.49 (−4.34 to 3.33)	−0.41 (−2.39 to 1.54)
Portugal	12 767.2 (10 436.9 to 15 414.9)	10 403.2 (8111.3 to 13 195.0)	10 300.5 (7884.5 to 13 062.5)	−2.05 (−6.42 to 2.34)	−0.09 (−4.68 to 4.33)	−1.02 (−3.19 to 1.07)
Republic of Korea	9730.8 (8013.9 to 11 862.7)	8380.9 (6713.2 to 10 359.9)	8072.3 (6291.0 to 10 160.3)	−1.49 (−5.69 to 2.57)	−0.34 (−4.53 to 3.77)	−0.89 (−3.02 to 1.13)
Slovakia	9855.0 (8046.6 to 11 929.1)	9257.7 (7454.7 to 11 337.4)	8882.1 (7059.6 to 11 035.3)	−0.63 (−4.70 to 3.43)	−0.38 (−4.31 to 3.57)	−0.49 (−2.50 to 1.50)
Slovenia	10 228.1 (8414.8 to 12 324.9)	8942.7 (7106.6 to 11 106.8)	8040.3 (6278.4 to 10 118.8)	−1.34 (−5.51 to 2.78)	−0.97 (−5.19 to 3.21)	−1.15 (−3.21 to 0.88)
Spain	10 504.4 (8401.9 to 13 110.5)	9184.5 (7075.4 to 11 805.5)	9271.9 (7093.0 to 11 922.9)	−1.34 (−6.17 to 3.40)	0.09 (−4.63 to 4.74)	−0.59 (−2.93 to 1.67)
Sweden	9391.8 (7413.0 to 11 813.7)	9558.3 (7473.8 to 12 087.4)	9271.5 (7172.9 to 11 809.8)	0.18 (−4.58 to 4.89)	−0.28 (−4.74 to 4.16)	−0.06 (−2.38 to 2.22)
Switzerland	11 313.5 (8976.3 to 13 926.6)	9954.6 (7690.7 to 12 595.2)	9494.6 (7206.9 to 12 100.8)	−1.28 (−5.94 to 3.39)	−0.43 (−5.08 to 4.12)	−0.83 (−3.14 to 1.42)
Turkey	11 881.9 (9955.3 to 14 225.7)	10 409.9 (8531.5 to 12 566.8)	10 593.6 (8450.6 to 13 029.0)	−1.32 (−5.11 to 2.33)	0.16 (−3.61 to 3.85)	−0.55 (−2.48 to 1.28)
United Kingdom	10 591.3 (8388.0 to 13 240.8)	10 031.1 (7819.2 to 12 674.7)	10 059.4 (7729.0 to 12 754.7)	−0.54 (−5.27 to 4.13)	0.03 (−4.50 to 4.45)	−0.25 (−2.56 to 2.00)
USA	12 357.3 (10 164.2 to 14 876.6)	12 017.6 (9687.8 to 14 615.3)	13 981.4 (11 282.5 to 16 895.1)	−0.28 (−4.29 to 3.63)	1.38 (−2.35 to 5.06)	0.59 (−1.32 to 2.42)

Source: https://vizhub.healthdata.org/gbd-results/.

ARoC, annualised rate of change, expressed as a percentage change per year in the rate per 100 000 population over the period 2000 to 2021; OECD, Organisation for Economic Co-operation and Development; UI, uncertainty intervals; USA, The United States of America.

Of the OECD countries, the USA (13 981.4 DALYs per 100 000 population), Colombia (13 183.3 DALYs per 100 000 population) and Mexico (13 043.8 DALYs per 100 000 population) were the top three countries with the highest rate of DALYs among adolescents and young adults in 2021. Slovenia (8040.3 DALYs per 100 000 population) and the Republic of Korea (8072.3 DALYs per 100 000 population) had the lowest DALYs among adolescents and young adults in 2021 (figure 1). Australia (10 363.9 DALYs per 100 000 population) was ranked eighth out of 38 OECD countries in DALYs in 2021 (table 3).

Stratified for age, there was a higher number of DALYs among young adults (20–24 years) and older adolescents (15–19 years) compared with young adolescents (10–14 years) residing in Australia and OECD countries in 2021 (online supplemental Table S5). In Australia, the rate of DALYs was higher among young adults (20–24 years; 14 104.5 DALYs per 100 000 population; 95% UI 11 113.0 to 17 197.7) followed by older adolescents (15–19 years; 10 868.0 DALYs per 100 000 population; 95% UI 8599.8 to 13 452.7) and younger adolescents (10–14 years: 6184.7 DALYs per 100 000 population; 95% UI 4741.6 to 7973.4). Similar trends were observed in OECD countries, with a high rate of DALYs among young adults (20–24 years; 15 319.0 DALYs per 100 000 population; 95% UI: 12 540.4–18 323.8) compared with older adolescents (15–19 years; 11 631.9 DALYs per 100 000 population; 95% UI: 9381.6–14 336.1) and younger adolescents (10–14 years; 6842.1 DALYs per 100 000 population; 95% UI: 5294.3–8701.0). In 2000, the rate of DALYs among young adults (20–24 years) was also estimated to be higher in Australia (16 557.9 DALYs per 100 000 population; 95% UI 13 490.6 to 19 956.0) and in OECD countries on average (14 878.2 DALYs per 100 000 population; 95% UI 12 358.8 to 17 649.1), compared with other age groups (ie, adolescents aged 10–19 years) (online supplemental Table S6). By gender, it was observed that in the year 2021, female adolescents and young adults had a higher number of DALYs compared with men, while there were slightly higher numbers of DALYs among male adolescents and young adults in OECD countries compared with females (online supplemental Table S7). Similarly, it was estimated that female adolescents and young adults residing in Australia in 2021 (10 748.3 DALYs per 100 000 population; 95% UI 8126.1 to 13 670.1) had a higher rate of DALYs compared with men (9999.3 DALYs per 100 000 population; 95% UI 8163.7 to 12 079.4; online supplemental Table S8). In the OECD countries, it was estimated that male adolescents and young adults in 2021 had a higher rate of DALYs (11 499.0 DALYs per 100 000 population; 95% UI 9687.1 to 13 712.3) compared with females (11 118.1 per 100 000 population; 95% UI 8547.8 to 14 205.8). In 2000, male adolescents and young adults in Australia (12 147.1 DALYs per 100 000 population; 95% UI 10 257.5 to 14 384.8) and in OECD countries (12 163.5 DALYs per 100 000 population; 95% UI 10 429.2 to 14 176.4) had a higher rate of DALYs compared with females.

### Risk factors of mortality in adolescents and young adults

**Figure 2:**
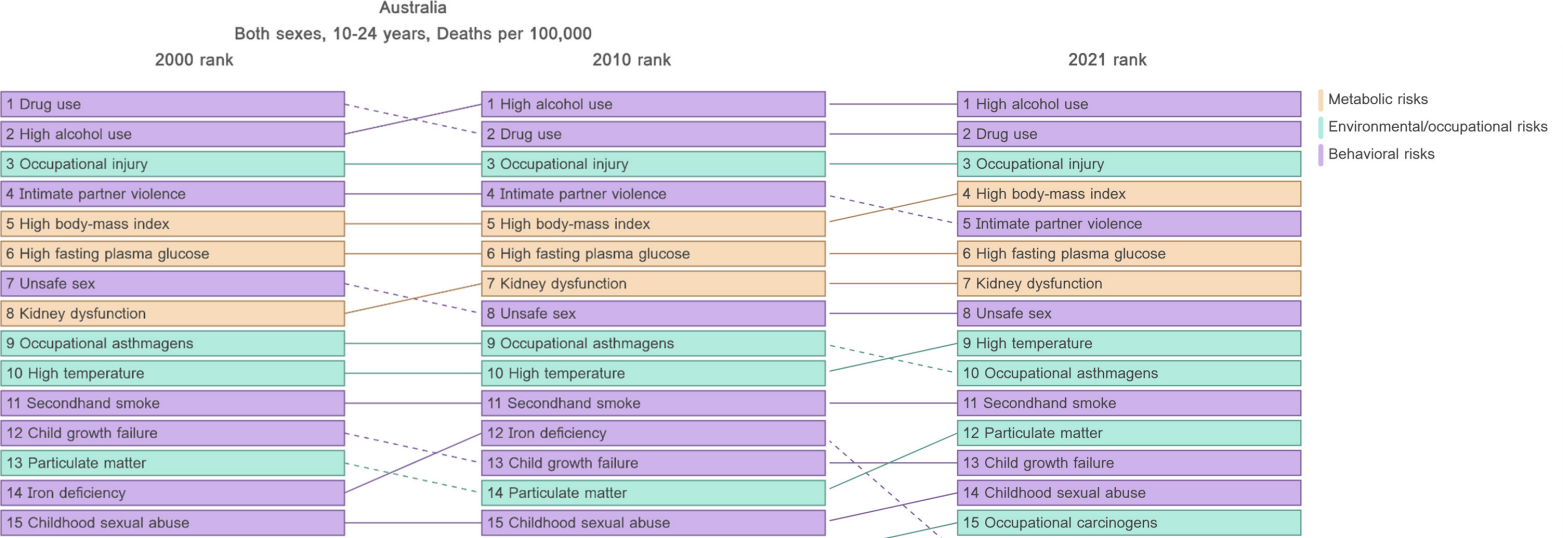
Top 15 risk factors of death for adolescent and young adults (10–24 years, both genders) in Australia in the years 2000, 2010 and 2021.

In both 2000 and 2021, high alcohol use, drug use, occupational injury, high BMI and intimate partner violence (IPV) were the five leading risk factors for death among adolescents and young adults in Australia (figure 2 and online supplemental figure S1). In OECD countries, the situation was slightly different. High alcohol use, drug use and occupational injury were also the leading risk factors for death among adolescents and young adults, followed by kidney dysfunction and high fasting plasma glucose. Unsafe sex was ranked fifth in the years 2000 and 2010 and was ranked sixth in 2021 (online supplemental figure S2).

Stratified for age and gender, high alcohol and drug use have been the leading risk factors across all age groups (ie, younger and older adolescents and young adults) and genders in Australia and OECD countries (online supplemental figure S3 to S6). With some variations in ranking, metabolic risk factors such as high BMI, high fasting plasma glucose and kidney dysfunction have also been the leading risk factors among adolescents and young adults in Australia and OECD countries in the years 2000 and 2021. Since 2010, secondhand smoke has been ranked the fifth leading cause of death among young adolescents residing in Australia (both males and females) and in OECD countries (only females). Occupational injuries were a predominant factor of death among older adolescents and young adults in Australia and OECD countries, especially among males.

Child growth failure and particulate matter (PM_2_._5_ air pollutant) were also among the leading risk factors of death among young adolescents (10–14 years) in OECD countries. Deaths due to high temperatures were ranked as the fifth leading risk factor for death among older adolescent males living in the OECD countries (online supplemental figures S3 and S4).

IPV and unsafe sex were the most common risk factors among female adolescents and young adults in Australia and OECD countries (online supplemental figures S5 and S6). Unsafe sex in Australia was ranked fifth among female young adolescents in the year 2000, which later dropped to the seventh position in 2021. Unsafe sex remained a leading risk factor among male adolescents and young adults in OECD countries. IPV has been ranked third among female adolescents and young adults in Australia since 2000. With a few shifts, IPV was also ranked third among female adolescents and young adults in OECD countries in 2021. The top 15 causes of death by gender, age and country are given in online supplemental figures S1-S6.

## Discussion

The study presents a comprehensive analysis of the number and rates of mortality and morbidity burden among adolescents and young adults in Australia compared with OECD countries from 2000 to 2021. The estimates suggest a steady decline in mortality rates in all age groups from 2000 to 2021. A higher burden of morbidity and mortality rates was observed among young adults (20–24 years) and older adolescents (15–19 years), with fewer deaths among young adolescents (10–14 years). These age-related patterns emerged as a crucial consideration in understanding the burden of disease and mortality risks among young adults (20–24 years), therefore highlighting specific vulnerabilities during this life transition. Gender disparities presented another important dimension of mortality and disease burden among adolescents and young adults. Higher mortality rates among males were observed across all ages, while a shift in the rate of DALYs shows a higher burden of disease among females in Australia, as compared with OECD countries on average. This unexpected change in disease burden patterns warrants further investigation into underlying causes and suggests the need for more gender-specific health interventions. The AIHW 2021 has also reported similar findings showing a higher mortality rate among young adults aged 20–24 years (ie, 48 deaths per 100 000 young people) compared with older adolescents aged 15–19 years (32 deaths per 100 000 young people).^27 28^ The recent AIHW 2023 report has also reported a decrease in deaths among young people (15–24 years) in Australia since 2009 and 2021, from 41 deaths per 100 000 to 38 deaths per 100 000, with higher mortality rates among males (52 per 100 000) compared with females (23 per 100 000).^29^ Similar trends were observed globally, with a higher number of deaths among young adults aged 20–24 years (n=7 39 860 deaths) compared with adolescents aged 15–19 years (n=5 71 849 deaths) in the year 2021.^30 31^ The WHO 2010–2018 report on the health of adolescents and youth in the Americas reported higher mortality rates among those aged 15–24 years compared with those aged 10–19 years, with 70%–80% deaths reported among males.^32^ These patterns were also evident across different regions and countries such as Afghanistan, Bangladesh, Canada, Chile, Colombia, Estonia, Ethiopia, India, Lithuania, Malawi, Mexico, New Zealand, Pakistan, Poland, Türkiye, Slovakia, USA, etc, where probability of dying was higher among adolescents aged 15–19 years compared with those aged 10–14 years in the year 2023.^33 34^

The country-specific data of this study showed higher mortality rates among adolescents and young adults in Mexico, Colombia and Costa Rica and higher DALYs in the USA, Colombia and Mexico. According to the United Nations International Children’s Emergency Fund 2023 data, Mexico and Colombia continued to report a higher probability of death among adolescents aged 10–19 years (six deaths/1000 children) in 2023, with an overall much higher probability of deaths in Türkiye (seven deaths/1000 children).^33^ The WHO data from 2013 to 16 also reported high mortality rates in Mexico and Colombia.^27 28^

The study identified several risk factors contributing to high mortality rates among adolescents and young adults in Australia and in OECD countries. High alcohol and drug use were identified as the leading risk factors for death among young adults, followed by metabolic risk factors such as high BMI, kidney dysfunction and high fasting plasma glucose. Environmental and occupational risks showed variation by age groups and countries. Gender and age-specific risk factors, including IPV and unsafe sex, were found to be prominent issues among females, with IPV being a persistent concern among females.^35^ Child growth failure, particulate matter and high temperatures were found as the leading factors of death among young adolescents in OECD countries, whereas secondhand smoking was also a contributing factor among younger adolescents in Australia and in OECD countries. According to the WHO reports, the rate of drinking is the highest among 15–19 year olds in the European region (45.9%) and in the Americas (43.9%).^36^ Similar to our study findings, the WHO 2021 data also identified IPV among the top three leading causes of death for adolescents aged 10–19 years in both the Americas and the European regions. In contrast, the Western Pacific region reported road injuries (5.50 deaths per 100 000 population) and drowning (4.17 deaths per 100 000) as the leading causes of adolescent mortality. However, it also reported kidney diseases (0.64 deaths per 100,000) as one of the leading causes of adolescent mortality in the Western Pacific region.^37^ The findings suggest lifestyle behaviours that develop in later adolescence and early adulthood, such as alcohol and drug use, are often linked to poor mental health, unsafe sexual practices and a high BMI, resulting from insufficient physical activity and unhealthy eating, which can have long-term consequences such as high blood pressure, diabetes and kidney diseases.^38^ These behaviours initiated during adolescence can impact health in later life.

Thus, recognising the growing health issues among adolescents and young adults, OECD countries, including Australia, have implemented diverse strategies to address several health challenges. For alcohol control, OECD countries have adopted the ‘PPPP strategy’ focusing on pricing policies, policing drunk driving, primary care counselling and protecting children from alcohol promotion.^39 40^ Recent innovations include minimum unit pricing targeting cheap alcohol consumed by heavy drinkers, implemented in countries like Ireland, Scotland and Wales, and the application of new rules requiring health warning labels on alcohol products.^39 40^ Australia has taken steps to address the issue of alcohol consumption and drug use among adolescents and young adults. The country has identified adolescents and young adults as a priority population in the National Drug Strategy 2017–2026.^41^ The National Health and Medical Research Council has also published the Australian Guidelines to reduce health risks from drinking alcohol.^42^ The government of Australia has also created an online portal called ‘Positive Choices’ to help schools and communities in Australia access up-to-date and accurate evidence and resources on alcohol and drugs.^43^ To fight obesity, OECD member countries have adopted various initiatives, including media campaigns promoting healthy eating, nutritional education, taxes on energy-dense foods, simplified food labelling and industry collaborations to improve product nutrition.^44^ These interventions, particularly food labelling and lifestyle-focused programmes, have proven both effective and adaptable across different national contexts. Despite these initiatives, progress has been relatively slow in OECD countries, including Australia.

The health of adolescents and young adults is influenced by several factors, such as socioeconomic status, family, peers, culture and environmental hazards. According to the Australian Unity Wellbeing Index, young people aged 18–25 had the lowest well-being scores compared with other age groups.^45^ This has been impacted by the COVID-19 pandemic and the increasing issue of climate change and environmental distress. Climate change is associated with an increased risk of post-traumatic stress disorder and other mental health issues among adolescents and young adults.^46 47^ Australia has faced climate catastrophes, including the Black Summer mega-fires in 2019/2020 and widespread flooding in 2020, 2021 and 2022.^48^ These catastrophes have resulted in extensive damage to infrastructure, homes, ecosystems and the health and well-being of adolescents and young adults.^48 49^ According to the 2022 Mission Australia Youth Survey, young people with high levels of climate change concerns were reported to have higher psychological distress and more negative outlooks about the future.^48^ Similar findings were reported in studies conducted in Canada^50^ and Norway.^51^ Climate change is another major public health and environmental concern, and there is a need for inter-sectoral policies to address the impact of climate change on the health of adolescents and young adults.

Another major concern among adolescents and young adults, specifically young women, is IPV. Violence against women has been labelled as the *shadow pandemic*.^52^ A recent study using the WHO Global Database on Prevalence of Violence Against Women estimated a 24% global prevalence of physical/sexual IPV in a lifetime against ever-partnered adolescent girls aged 15–19 years.^53^ The prevalence of physical or sexual (or both) IPV in the lifetime of 15–19 year-old girls was reported to be highest in the Oceania region (47%) and lower-rated in central Europe (20%).^53^ According to Growing Up in Australia: The Longitudinal Study of Australian Children, 29% of adolescents aged 18–19 years have reported some form of IPV.^35^ About one in four adolescents reported experiencing emotional abuse, one in eight reported experiencing physical violence and 1 in 12 reported experiencing sexual abuse. The survey also reported that young women are more likely to be victims of sexual abuse compared with men. Several countries, including Australia, have adopted an integrated approach to address gender-based violence (GBV). Australia’s National Plan to End Violence against Women and Children, Spain’s State Pact against Gender Violence and Contingency Plan, Sweden’s National Strategy to Prevent and Combat Men’s Violence Against Women and Switzerland’s National Action Plan 2022–2026 are some great examples of countries' contribution to reduced IPV, but much of the work on GBV faces insufficient case-reported funding and inadequate coordination among the stakeholders involved.^54^

The health of adolescents and young adults has received insufficient attention in public health policy; however, the rising disease burden and poor lifestyle behaviours in adolescence have long-term health impacts in adulthood.^38^ The data on risk factors for mortality suggest a life course approach with a prominent focus on adolescents and young adults. The findings suggest gender and age-specific healthcare policies and interventions with a slight shift in focus from the health of women to the health of men and from children to young adults. These shifts may challenge the political willingness to invest in programmes that may take years to see some beneficial impacts.^38^

While Australia has made some progress in reducing adolescent and young adult’s mortality rates, it has shown a minimal increase in DALYs over the study period. This disparity between mortality and DALYs points to the need for more comprehensive health strategies that address not just survival but also better quality of life for adolescents and young adults. The fact that Australia ranks among the top eight OECD countries for DALYs, despite its relatively low mortality rate, further emphasises this challenge.

### Limitations

The study has some limitations. First, the GBD Study 2021 uses different datasets, which may be a potential source of bias, such as missing data for location years. Second, the study did not fully account for the impact of the COVID-19 pandemic across most risk factors and health outcomes. Considerations should be taken into account because the impact of COVID-19 varies in different countries and regions due to different political situations and the implementation of policies.^55^ The third limitation is that the mortality estimates are conducted through modelling, which could be sensitive to the quality of the primary studies; however, the associated bias is not greater for the study countries.^19 56^ Finally, unlike the estimates for all population, our study focuses on a specific age group (ie, 10–24 years); therefore, all mortality rates are presented as crude age-specific rates. In line with the GBD methodology, age-standardised rates are only provided for the total population, while crude age-specific rates are reported for defined age groups, including ages 10–24 years. These age-specific rates are considered directly comparable across countries, as they are expressed per population within the respective age group. While the influence of age distribution within this relatively narrow age band is likely minimal, however, some residual bias may remain.

## Conclusion

Overall mortality has declined in Australia and OECD countries on average, but alcohol and drug use, metabolic risks and age-specific and gender-specific factors remain significant challenges. Understanding adolescent and young adult morbidity and mortality trends is essential to guide investments and policies. Most deaths are preventable, highlighting the need for interventions that improve both current and future generations’ health.

## Supplementary material

10.1136/bmjph-2025-002986 online supplemental file 1

## Data Availability

All data relevant to the study are included in the article or uploaded as supplementary information.

## References

[1] Australian Institute of Health and Welfare (AIHW) (2024). Health of young people. https://www.aihw.gov.au/reports/children-youth/health-of-young-people.

[2] Langley JM (2015). Adolescent immunization - Protecting youth and preparing them for a healthy future. Int J Pediatr Adolesc Med.

[3] World Health Organisation (WHO) (2023). Adolescent health. overview. https://www.who.int/health-topics/adolescent-health#tab=tab_1.

[4] Ward JL, Azzopardi PS, Francis KL (2021). Global, regional, and national mortality among young people aged 10–24 years, 1950–2019: a systematic analysis for the Global Burden of Disease Study 2019. The Lancet.

[5] Deželan T (2024). Handbook of Equality of Opportunity.

[6] World Health Organization (WHO) (2023). Global accelerated action for the health of adolescents (AA-HA!): guidance to support country implementation.

[7] WHO, UNAIDS, UNFPA, UNICEF, UNWomen, The World Bank Group (2018). Survive, thrive, transform. global strategy for women’s, children’s and adolescents’ health: 2018 report on progress towards 2030 targets.

[8] Boerma T, Requejo J, Victora CG (2018). Countdown to 2030: tracking progress towards universal coverage for reproductive, maternal, newborn, and child health. The Lancet.

[9] Armocida B, Monasta L, Sawyer S (2022). Burden of non-communicable diseases among adolescents aged 10–24 years in the EU, 1990–2019: a systematic analysis of the Global Burden of Diseases Study 2019. *The Lancet Child & Adolescent Health*.

[10] OECD; World Bank (2023). Health at a Glance: Latin America and the Caribbean 2023.

[11] The Australian Research Alliance for Children and Youth (ARACY); RAND Australia; UNICEF Australia (2018). REPORT CARD 2018, in The Wellbeing of young Australians.

[12] Melaku YA, Appleton SL, Gill TK (2018). Incidence, prevalence, mortality, disability-adjusted life years and risk factors of cancer in Australia and comparison with OECD countries, 1990-2015: findings from the Global Burden of Disease Study 2015. Cancer Epidemiol.

[13] Collaborators G (2023). The burden and trend of diseases and their risk factors in Australia, 1990-2019: a systematic analysis for the Global Burden of Disease Study 2019.

[14] Australian Institute of Health and Welfare (AIHW) (2023). Australian Burden of Disease Study 2023.

[15] Institute for Health Metrics and Evaluation (IHME) (2024). Global burden of disease (GBD). https://www.healthdata.org/research-analysis/gbd.

[16] Naghavi M, Ong KL, Aali A (2024). Global burden of 288 causes of death and life expectancy decomposition in 204 countries and territories and 811 subnational locations, 1990–2021: a systematic analysis for the Global Burden of Disease Study 2021. The Lancet.

[17] Institute for Health Metrics and Evaluation (IHME) (2024). GBD results. https://vizhub.healthdata.org/gbd-results.

[18] Schumacher AE (2024). Global age-sex-specific mortality, life expectancy, and population estimates in 204 countries and territories and 811 subnational locations, 1950–2021, and the impact of the COVID-19 pandemic: a comprehensive demographic analysis for the Global Burden of Disease Study 2021. The Lancet.

[19] Ferrari AJ (2024). Global incidence, prevalence, years lived with disability (YLDs), disability-adjusted life-years (DALYs), and healthy life expectancy (HALE) for 371 diseases and injuries in 204 countries and territories and 811 subnational locations, 1990–2021: a systematic analysis for the Global Burden of Disease Study 2021. The Lancet.

[20] Schumacher AE (2024). Request Global age-sex-specific mortality, life expectancy, and population estimates in 204 countries and territories and 811 subnational locations, 1950–2021, and the impact of the COVID-19 pandemic: a comprehensive demographic analysis for the Global Burden of Disease Study 2021.

[21] Institute for Health Metrics and Evaluation (IHME) (2024). GBD 2021 data and tools overview.

[22] Sheena BS, Hiebert L, Han H (2022). Global, regional, and national burden of hepatitis B, 1990–2019: a systematic analysis for the Global Burden of Disease Study 2019. *The Lancet Gastroenterology & Hepatology*.

[23] Tessema GA, Berheto TM, Pereira G (2023). National and subnational burden of under-5, infant, and neonatal mortality in Ethiopia, 1990-2019: Findings from the Global Burden of Disease Study 2019. *PLOS Glob Public Health*.

[24] Sawyer SM, Afifi RA, Bearinger LH (2012). Adolescence: a foundation for future health. The Lancet.

[25] Brauer M, Roth GA, Aravkin AY (2024). Global burden and strength of evidence for 88 risk factors in 204 countries and 811 subnational locations, 1990–2021: a systematic analysis for the Global Burden of Disease Study 2021. The Lancet.

[26] Alam K (2024). Global burden of 288 causes of death and life expectancy decomposition in 204 countries and territories and 811 subnational locations, 1990-2021: a systematic analysis for the Global Burden of Disease Study 2021. The Lancet (British Edition).

[27] Australian Institute of Health and Welfare (AIHW) (2021). Australia’s health 2018. deaths in Australia. cat. no.PHE 229. australia’s health series no.16. AUS 221. https://www.aihw.gov.au/reports/children-youth/deaths.

[28] Australian Institute of Health and Welfare (AIHW) (2018). Australia’s health 2018. deaths in Australia. cat. no.PHE 229. australia’s health series no.16. AUS 221. https://www.aihw.gov.au/reports/children-youth/deaths.

[29] Australian Institute of Health and Welfare. (AIHW) (2024). Health of young people.

[30] The World Bank Group (2025). Data. number of deaths ages 15-19 years 2025. https://data.worldbank.org/indicator/SH.DTH.1519?locations=BD.

[31] The World Bank Group (2025). Data. number of deaths ages 20-24 years 2025. https://data.worldbank.org/indicator/SH.DTH.2024.

[32] Pan American Health Organization; World Health Organization Regional Office of Americas. The Health of Adolescents and Youth in the Americas (2018). Summary Report., in Chapter II: The Current Status of the Health of Adolescents and Youth in the Americas.

[33] The United Nations International Children’s Emergency Fund (2025). Adolescent data portal. using data to better understand the lives of adolescents. compare countries by health. all cause mortality rate 2023. https://data.unicef.org/adp/compare-countries/?health=All+cause+mortality+rate.

[34] The United Nations International Children’s Emergency Fund (2023). Adolescent health dashboards. https://data.unicef.org/resources/adolescent-health-dashboards-country-profiles.

[35] O’Donnell K (2023). Intimate partner violence among australian 18–19 year olds, in growing up in australia snapshot series – issue 11.

[36] The World Health Organization (2025). Over 3 million annual deaths due to alcohol and drug use, majority among men.

[37] The World Health Organization (2025). Adolescent mortality rate (per 100 000 population). adolescent mortality rate - top 20 causes (global and regions). https://www.who.int/data/gho/indicator-metadata-registry/imr-details/4751.

[38] Gore FM, Bloem PJ, Patton GC (2011). Global burden of disease in young people aged 10–24 years: a systematic analysis. The Lancet.

[39] OECD (2023). Alcohol consumption. health at a glance 2023.

[40] Australian Institute of Health and Welfare (AIHW) (2021). Alcohol, tobacco and other drugs. https://www.aihw.gov.au/reports/children-youth/alcohol-tobacco-and-other-drugs.

[41] Department of health, australia (2017). National drug strategy 2017–2026, in National Drug Strategy.

[42] National Health and Medical Research Council (NHMRC) (2020). Australian guidelines to reduce health risks from drinking alcohol.

[43] The Australian Government Department of Health and Aged Care and University of Sydney (2024). Positive choices. https://positivechoices.org.au/information/about-positive-choices.

[44] OECD (2023). Overweight and obesity. health at a glance 2023.

[45] Deakin University and Australian Unity (2022). The declining wellbeing of Australians in 2022, in Our wellbeing in challenging times.

[46] Wu J, Snell G, Samji H (2020). Climate anxiety in young people: a call to action. Lancet Planet Health.

[47] Crandon TJ, Scott JG, Charlson FJ (2022). A social–ecological perspective on climate anxiety in children and adolescents. Nat Clim Chang.

[48] Teo SM (2024). Climate change concerns impact on young Australians’ psychological distress and outlook for the future. J Environ Psychol.

[49] Climate Council (2023). Summary of results from national study of the impact of climate-fuelled disasters on the mental health of australians.

[50] Galway LP, Field E (2023). Climate emotions and anxiety among young people in Canada: A national survey and call to action. *The Journal of Climate Change and Health*.

[51] Leonhardt M, Granrud MD, Bonsaksen T (2022). Associations between Mental Health, Lifestyle Factors and Worries about Climate Change in Norwegian Adolescents. Int J Environ Res Public Health.

[52] UN Women (2021). The shadow pandemic: violence against women during COVID-19. https://www.unwomen.org/en/news/in-focus/in-focus-gender-equality-in-covid-19-response/violence-against-women-during-covid-19.

[53] Sardinha L, Yüksel-Kaptanoğlu I, Maheu-Giroux M (2024). Intimate partner violence against adolescent girls: regional and national prevalence estimates and associated country-level factors. *The Lancet Child & Adolescent Health*.

[54] OECD (2023). Supporting lives free from intimate partner violence: towards better integration of services for victims/survivors.

[55] Chen S, Huang W, Zhang M (2025). Dynamic changes and future trend predictions of the global burden of anxiety disorders: analysis of 204 countries and regions from 1990 to 2021 and the impact of the COVID-19 pandemic. EClinicalMedicine.

[56] Vollset SE (2024). Burden of disease scenarios for 204 countries and territories, 2022–2050: a forecasting analysis for the Global Burden of Disease Study 2021. The Lancet.

