# Supplementary material for: Burdens and risk factors for mortality in adolescents and young adults aged 10–24 years in Australia and comparison with OECD countries between 2000 and 2021: Global Burden of Disease Study 2021
Source: BMJ Public Health. 2025 Nov 28;3(2):e002986. doi: 10.1136/bmjph-2025-002986 (PMC12666037; doi:10.1136/bmjph-2025-002986)

Supplementary File

Table of Contents

[*Table S1: Number of mortalities/deaths among adolescents and young adults stratified by age in years 2000, 2010, and 2021* 2](#_Toc211787533)

[Table S2: Rate of mortality among adolescents and young adults stratified by age in years 2000, 2010, and 2021 4](#_Toc211787534)

[Table S3: Number of mortalities/deaths among adolescents and young adults aged 10-24 years in 2000, 2010, and 2021, stratified by gender 5](#_Toc211787535)

[Table S4: Rate of mortality among adolescents and young adults aged 10-24 years in 2000, 2010, and 2021, stratified by gender 6](#_Toc211787536)

[Table S5: Number of DALYs among adolescents and young adults (both genders) in years 2000, 2010, and 2021, stratified by age 7](#_Toc211787537)

[Table S6: Rate of DALYs among adolescents and young adults (both genders) in years 2000, 2010, and 2021, stratified by age 9](#_Toc211787538)

[Table S7: Number of DALYs among adolescents and young adults aged 10-24 years in 2000, 2010, and 2021, stratified by gender 11](#_Toc211787539)

[Table S8: Rate of DALYs among adolescents and young adults aged 10-24 years in 2000, 2010, and 2021, stratified by gender 13](#_Toc211787540)

[Figure S1: Top 15 risk factors of death for adolescent and young adults (10-24 years, both genders) in Australia in the years 2000, 2010, and 2021 14](#_Toc211787541)

[Figure S2: Top 15 risk factors of death for adolescents and young adults (10-24 years) in OECD Countries in the years 2000, 2010, and 2021 17](#_Toc211787542)

[Figure S3: Top 15 risk factors of death for male adolescents and young adults (10-24 years) in OECD Countries in the years 2000, 2010, and 2021 21](#_Toc211787543)

[Figure S4: -Top 15 risk factors of death for male adolescents and young adults (10-24 years) in Australia in the years 2000, 2010, and 2021 25](#_Toc211787544)

[Figure S5: -Top 15 risk factors of death for female adolescents and young adults (10-24 years) in OECD Countries in the years 2000, 2010, and 2021 29](#_Toc211787545)

[Figure S6: Top 15 risk factors of death for female adolescents and young adults (10-24 years) in Australia in the years 2000, 2010, and 2021 33](#_Toc211787546)

#### *Table S1: Number of mortalities/deaths among adolescents and young adults stratified by age in years 2000, 2010, and 2021*

| Country | Number of Deaths (95% UI) | | | | | | | | |
| --- | --- | --- | --- | --- | --- | --- | --- | --- | --- |
|  | 2000 | | | 2010 | | | 2021 | | |
|  | 10-14 years | 15-19 years | 20-24 years | 10-14 years | 15-19 years | 20-24 years | 10-14 years | 15-19 years | 20-24 years |
| *All OECD countries average* | *20416.4 (20075.8 - 20739.6)* | *54915.8 (54309.7 - 55573.5)* | *74103.4 (73471.1 - 74786.9)* | *15173.2 (15602.1 - 14729.5)* | *42674.2 (43340.1 - 42028.2)* | *64289.6 (64656.4 - 63938.7)* | *13120.0 (12623.3 - 13692.9)* | *38461.2 (37523.2 – 39497.0)* | *67089.7 (66191.1 - 68000.7)* |
| Australia | 194.8 (191.5 - 198.1) | 699.6 (691.5 – 708.0) | 967.0 (958.9 - 975.4) | 139.5 (141.9 - 137.1) | 519.9 (526.2 - 513.7) | 735.1 (740.9 - 729.1) | 138.3 (131.8 - 144.9) | 447.6 (435.5 - 459.6) | 734.4 (730.7 – 738.0) |
| Austria | 67.9 (66.3 - 69.6) | 277.0 (272.8 - 281.4) | 320.7 (318.2 - 323.4) | 44.2 (45.5 – 43.0) | 183.9 (187.1 - 180.8) | 283.8 (286.6 – 281.0) | 27.6 (26.5 - 28.9) | 123.5 (120.1 - 127.2) | 188.2 (185.8 - 190.8) |
| Belgium | 97.1 (94.9 - 99.1) | 319.3 (314.7 - 323.3) | 517.7 (513.3 - 521.9) | 67.3 (68.9 - 65.6) | 198.7 (201.8 - 195.9) | 348.1 (351.0 - 344.9) | 59.9 (56.8 – 63.0) | 158.8 (153.3 - 164.6) | 232.8 (229.9 - 235.8) |
| Canada | 318.4 (313.6 - 323.2) | 1044.8 (1035.2 - 1054.8) | 1295.3 (1288.4 – 1303.0) | 221.8 (225.3 - 218.4) | 866.5 (874.2 - 857.9) | 1224.9 (1231.5 - 1218.6) | 219.4 (210.9 - 228.4) | 711.8 (693.3 - 730.1) | 1379.0 (1346.2 - 1421.4) |
| Chile | 310.0 (305.7 - 314.5) | 682.1 (675.3 - 688.7) | 964.8 (958.2 - 971.9) | 256.8 (261.6 - 252.1) | 744.5 (753.2 - 736.1) | 1078.0 (1086.9 - 1069.3) | 185.6 (180.9 - 189.9) | 456.3 (448.4 - 464.5) | 932.7 (924.2 - 941.9) |
| Colombia | 2072.8 (1975.8 - 2173.8) | 7353.1 (7134.9 – 7597.0) | 9815.3 (9612.4 - 10059.7) | 1639.2 (1747.6 – 1533.0) | 4425.0 (4571.1 - 4280.1) | 6785.8 (6838.4 - 6736.2) | 1129.5 (1011.3 - 1263.6) | 3458.1 (3062.7 - 3868.2) | 5825.9 (5214.7 - 6458.7) |
| Costa Rica | 107.9 (105.5 - 110.2) | 237.2 (231.6 - 242.4) | 293.7 (287.3 - 300.2) | 98.5 (100.8 - 96.2) | 247.7 (253.5 - 242.2) | 366.7 (374.1 - 359.7) | 91.8 (88.6 - 94.8) | 190.7 (184.6 - 196.2) | 420.8 (410.4 - 431.5) |
| Czechia | 120.9 (117.5 - 124.5) | 345.7 (339.3 - 352.6) | 608.4 (602.0 - 615.1) | 59.8 (61.4 - 58.1) | 214.3 (218.7 – 210.0) | 380.7 (387.4 - 374.4) | 61.0 (58.5 - 63.4) | 125.6 (122.1 – 129.0) | 203.7 (199.8 - 208.2) |
| Denmark | 45.5 (44.3 - 46.8) | 123.1 (121.1 – 125.0) | 180.2 (178.3 - 182.1) | 25.2 (26.1 - 24.3) | 86.0 (87.9 – 84.0) | 121.5 (123.0 - 120.1) | 21.9 (20.9 - 22.9) | 59.0 (57.1 - 60.9) | 117.1 (115.1 - 119.3) |
| Estonia | 28.4 (27.5 - 29.4) | 80.9 (78.8 - 83.2) | 144.3 (141.2 - 147.5) | 9.8 (10.2 - 9.5) | 34.9 (36.0 - 33.9) | 83.7 (86.1 - 81.4) | 9.7 (9.1 - 10.3) | 30.4 (29.2 - 31.7) | 35.8 (34.6 - 37.2) |
| Finland | 45.0 (43.5 - 46.6) | 164.2 (160.7 - 167.6) | 249.8 (247.0 - 252.3) | 29.6 (30.8 - 28.5) | 123.5 (126.6 - 120.5) | 225.1 (227.9 - 221.9) | 29.6 (28.1 - 31.2) | 108.4 (104.3 - 112.7) | 164.0 (160.9 - 167.2) |
| France | 604.8 (597.7 - 611.7) | 1936.0 (1922.0 - 1949.9) | 2642.1 (2630.8 - 2652.4) | 378.9 (383.7 – 374.0) | 1207.5 (1216.8 - 1198.6) | 1920.3 (1929.1 - 1912.2) | 312.6 (307.3 - 317.8) | 794.1 (785.3 - 803.7) | 1489.0 (1479.5 - 1499.5) |
| Germany | 630.8 (623.7 – 638.0) | 2101.1 (2087.8 - 2114.8) | 2795.5 (2786.0 - 2806.2) | 381.5 (387.0 - 376.4) | 1138.6 (1148.5 - 1129.5) | 1880.8 (1888.3 - 1874) | 306.5 (300.9 - 311.8) | 889.5 (878.4 - 900.5) | 1348.0 (1340.0 - 1355.5) |
| Greece | 87.2 (85.2 - 89.4) | 367.5 (362.5 - 373) | 612.8 (607.4 - 618.2) | 65.2 (66.8 - 63.6) | 216.9 (220.3 - 213.5) | 386.1 (389.7 - 382.1) | 41.7 (39.5 - 44.1) | 132.0 (127.2 - 136.6) | 225.1 (218.0 - 231.1) |
| Hungary | 130.3 (127.6 - 133.1) | 283.2 (279.2 - 287.4) | 527.9 (523.4 - 532.9) | 70.8 (72.6 - 69.1) | 203.3 (206.5 - 200.2) | 281.3 (284.3 - 278.4) | 38.2 (37.0 - 39.5) | 149.7 (146.4 - 153.3) | 257.4 (254.2 - 260.6) |
| Iceland | 2.1 (2.0 - 2.2) | 10.4 (10.0 - 10.9) | 15.2 (14.6 - 15.7) | 2.5 (2.7 - 2.4) | 7.1 (7.5 - 6.8) | 9.6 (10.1 - 9.2) | 3.4 (3.3 - 3.5) | 5.1 (4.6 - 5.6) | 7.8 (7.4 - 8.3) |
| Ireland | 50.3 (48.9 - 51.5) | 191.4 (187.7 - 194.8) | 256.1 (252.8 - 259.7) | 28.9 (29.8 – 28.0) | 109.7 (111.9 - 107.5) | 172.5 (174.8 - 169.9) | 17.5 (16.6 - 18.3) | 60.2 (58.2 - 62.4) | 81.6 (80 - 83.5) |
| Israel | 91.0 (89.2 - 92.9) | 270.0 (266.3 - 273.3) | 325.3 (321.9 - 328.7) | 79.4 (81.3 - 77.6) | 169.8 (172.2 - 167.2) | 245.0 (247.8 - 242.1) | 71.5 (68.7 - 74.5) | 227.9 (223.7 - 232.5) | 286.3 (281.5 - 291.8) |
| Italy | 419.3 (413.7 - 424.6) | 1273.5 (1264.1 - 1283.2) | 2192.9 (2183.9 - 2202.2) | 292.6 (297.0 - 288.1) | 832.0 (839.1 - 824.6) | 1217.7 (1223.1 – 1212.0) | 227.0 (222.7 - 231.5) | 565.2 (558.7 – 572.0) | 768.7 (763.5 - 773.9) |
| Japan | 765.9 (757.2 - 773.6) | 2491.5 (2475.4 - 2506.7) | 4087.6 (4071.6 - 4104.2) | 540.9 (546.7 - 534.4) | 1461.0 (1471.0 - 1450.5) | 2853.5 (2864.6 - 2843.1) | 442.5 (434.6 - 450.2) | 1205.9 (1192.9 - 1218.4) | 2180.8 (2168.6 - 2192.6) |
| Latvia | 55.7 (54.3 - 57.1) | 147.4 (144.6 - 150.4) | 248.7 (244.4 - 253.1) | 22.2 (22.9 - 21.6) | 70.1 (71.8 - 68.4) | 133.7 (136.4 - 130.9) | 8.5 (8.1 - 8.9) | 28.5 (27.6 - 29.4) | 67.3 (65.6 – 69.0) |
| Lithuania | 77.4 (75.7 - 79.3) | 232.5 (228.4 - 236.5) | 364.5 (358.7 - 370.1) | 37.2 (38.3 - 36.1) | 149.9 (153.2 - 146.8) | 253.1 (257.3 – 249.0) | 21.4 (20.4 - 22.3) | 44.3 (42.9 - 45.7) | 103.0 (100.8 - 105.2) |
| Luxembourg | 2.6 (2.5 - 2.8) | 10.2 (9.8 - 10.6) | 18.0 (17.4 - 18.6) | 2.0 (2.2 - 1.9) | 7.3 (7.7 - 6.9) | 13.0 (13.5 - 12.5) | 2.0 (1.9 - 2.2) | 8.2 (7.6 - 8.8) | 11.2 (10.6 - 11.9) |
| Mexico | 4320.1 (4146.0 - 4516.7) | 7601.4 (7430.3 - 7778.7) | 10053.5 (10011.5 - 10094.6) | 3962.3 (4192.3 - 3740.4) | 9712.9 (10006.6 - 9416.2) | 12866.1 (12912.8 - 12818.9) | 3492.2 (3268.0 - 3754.4) | 9399.9 (9073.4 - 9753.3) | 16095.8 (16031 - 16157.8) |
| Netherlands | 143.5 (140.5 - 146.2) | 346.7 (342.5 - 350.9) | 455.5 (452.2 - 458.9) | 98.7 (100.9 - 96.5) | 221.0 (224.1 - 218.1) | 315.5 (318.5 - 312.4) | 79.3 (77.0 - 81.9) | 166.0 (162.3 - 169.7) | 343.5 (339.5 - 347.7) |
| New Zealand | 66.2 (64.4 – 68.0) | 187.8 (184.8 - 191.2) | 208.6 (206.1 - 211.2) | 46.2 (47.6 – 45.0) | 175.7 (179.1 - 172.6) | 204.1 (206.6 - 201.8) | 38.4 (37.0 - 39.5) | 124.1 (121.3 - 126.7) | 178.9 (176.4 - 181.4) |
| Norway | 32.9 (31.9 - 33.9) | 141.7 (139.1 - 144.2) | 201.7 (199.2 – 204.0) | 31.7 (32.8 - 30.7) | 119.9 (122.2 - 117.7) | 165.4 (167.4 - 163.4) | 34.2 (32.9 - 35.7) | 84.9 (82.7 - 87.3) | 114.4 (112.6 - 116.3) |
| Poland | 567.4 (559.8 - 574.5) | 1749.9 (1735.3 - 1763.3) | 2431.6 (2419.3 - 2444.2) | 310.6 (315.0 - 306.3) | 1123.3 (1132.8 - 1113.1) | 1918.6 (1930.9 - 1907.6) | 220.1 (216.1 – 224.0) | 668.3 (660.8 - 675.8) | 1150.7 (1142.9 - 1158.8) |
| Portugal | 142.6 (139.3 - 145.7) | 443.7 (437.5 - 449.8) | 767.1 (761.5 - 773.2) | 69.7 (71.6 - 67.8) | 167.3 (170.1 - 164.6) | 294.1 (297.2 – 291.0) | 49.6 (47.3 - 51.8) | 121.9 (118.3 - 125.6) | 184.1 (181.7 - 186.5) |
| Republic of Korea | 616.0 (609.4 - 622.7) | 1751.0 (1739.9 - 1763.3) | 2469.5 (2457.6 - 2481.1) | 421.2 (426.7 - 416.2) | 1043.6 (1052.4 - 1035.5) | 1402.8 (1410.5 – 1395.0) | 199.6 (188.9 – 210.0) | 506.9 (487.1 - 527.7) | 1018.6 (995.5 - 1042.6) |
| Slovakia | 94.5 (91.6 - 97.5) | 196.7 (192.6 - 201.2) | 321.1 (316.7 - 325.7) | 52.7 (54.1 - 51.3) | 128.3 (130.9 - 125.9) | 231.9 (235.5 - 228.4) | 34.9 (33.5 - 36.2) | 88.7 (86.2 – 91.0) | 135.7 (133.4 – 138.0) |
| Slovenia | 19.1 (18.5 - 19.8) | 79.3 (77.0 - 81.5) | 118.0 (115.2 - 120.8) | 8.8 (9.2 - 8.4) | 32.1 (33.3 - 30.9) | 64.9 (66.7 - 63.3) | 7.9 (7.4 - 8.4) | 26.4 (25.0 - 27.9) | 27.0 (25.9 - 28.1) |
| Spain | 365.6 (359.6 - 371.5) | 1195.1 (1183.3 - 1206.7) | 1962.8 (1952.5 – 1974.0) | 229.0 (232.6 - 225.1) | 535.3 (540.2 - 529.8) | 834.6 (839.1 - 830.4) | 212.1 (207.4 – 217.0) | 420.6 (414.1 – 427.0) | 669.2 (663.7 - 674.6) |
| Sweden | 71.3 (69.5 - 73.2) | 164.5 (161.9 - 167.1) | 258.1 (255.7 - 260.5) | 46.8 (48.1 - 45.5) | 173.5 (176.4 - 170.8) | 279.5 (282.2 – 277.0) | 47.5 (44.5 - 50.6) | 120.7 (110.5 - 130.3) | 226.4 (207.7 - 244.2) |
| Switzerland | 68.4 (66.6 - 70.1) | 170.4 (167.6 - 173.2) | 274.5 (272.0 - 277.3) | 37.3 (38.5 - 36.3) | 126.1 (128.3 - 123.9) | 161.8 (163.7 - 160.1) | 33.4 (32.2 - 34.7) | 89.2 (86.8 - 91.8) | 128.0 (126.1 - 130.1) |
| Türkiye | 2913.1 (2692.1 - 3142.5) | 5070.3 (4573.4 - 5616.2) | 5327.6 (4805.7 - 5936.2) | 2008.5 (2176.2 - 1850.2) | 3550.1 (3884.1 - 3235.9) | 3803.4 (4138.8 - 3481.4) | 1464.1 (1318.4 - 1615.9) | 3066.8 (2632.4 - 3526.6) | 3919.7 (3285.4 - 4616.8) |
| United Kingdom | 548.2 (535.2 – 561.0) | 1512.3 (1492.0 - 1529.9) | 2051.9 (2043.2 - 2060.3) | 369.2 (373.5 – 365.0) | 1189.1 (1198.1 - 1180.6) | 1808.7 (1817.4 - 1800.9) | 363.3 (357.9 - 368.7) | 979.7 (970.2 - 988.7) | 1682.3 (1668.3 - 1694.7) |
| United States of America | 4121.0 (4102.1 - 4142.2) | 13663.5 (13619.6 – 13709.0) | 17758.2 (17721.2 - 17793.6) | 2986.8 (3001.8 - 2972.5) | 11157.8 (11198.6 - 11119.5) | 18938.0 (18984.8 - 18890.8) | 3376.3 (3296.0 - 3471.1) | 12616.2 (12421.8 - 12832.5) | 24154.6 (23897.2 - 24415.6) |
| *UI: uncertainty intervals | | | | | | | | | |

#### Table S2: Rate of mortality among adolescents and young adults stratified by age in years 2000, 2010, and 2021

| Country | Mortality Rate per 100,000 population (95% UI) | | | | | | | | |  |
| --- | --- | --- | --- | --- | --- | --- | --- | --- | --- | --- |
|  | 2000 | | | 2010 | | | 2021 | | |  |
|  | 10-14 years | 15-19 years | 20-24 years | 10-14 years | 15-19 years | 20-24 years | 10-14 years | 15-19 years | 20-24 years |  |
| *All OECD countries average* | *23.4 (23.0 - 23.8)* | *61.9 (61.2 - 62.7)* | *84.2 (83.5 – 85.0)* | *18.0 (17.5 - 18.5)* | *48.6 (47.9 - 49.4)* | *72.4 (72.1 - 72.9)* | *15.7 (15.1 - 16.3)* | *46.0 (44.9 - 47.3)* | *77.2 (76.2 - 78.3)* |  |
| Australia | 14.4 (14.1 - 14.6) | 52.2 (51.6 - 52.8) | 76.6 (76.0 - 77.3) | 9.9 (9.8 - 10.1) | 36.1 (35.7 - 36.5) | 50.0 (49.6 - 50.4) | 8.5 (8.1 - 8.9) | 30.1 (29.2 - 30.9) | 45.5 (45.3 - 45.8) |  |
| Austria | 14.5 (14.1 - 14.9) | 57.1 (56.2 – 58.0) | 68.8 (68.3 - 69.4) | 10.1 (9.8 - 10.4) | 37.1 (36.4 - 37.7) | 54.5 (53.9 – 55.0) | 6.4 (6.2 - 6.7) | 27.0 (26.2 - 27.8) | 35.9 (35.4 - 36.4) |  |
| Belgium | 15.8 (15.5 - 16.2) | 52.4 (51.7 - 53.1) | 82.0 (81.3 - 82.7) | 11.1 (10.8 - 11.3) | 30.5 (30.1 – 31.0) | 52.5 (52.0 - 52.9) | 8.9 (8.5 - 9.4) | 25.1 (24.3 – 26.0) | 35.2 (34.8 - 35.7) |  |
| Canada | 15.3 (15.1 - 15.5) | 50.0 (49.6 - 50.5) | 65.6 (65.2 – 66.0) | 11.1 (10.9 - 11.3) | 38.7 (38.3 - 39.1) | 55.7 (55.4 - 56) | 10.1 (9.7 - 10.5) | 34.6 (33.7 - 35.5) | 61.7 (60.2 - 63.6) |  |
| Chile | 22.0 (21.7 - 22.3) | 52.3 (51.7 - 52.8) | 82.0 (81.5 - 82.6) | 20.1 (19.7 - 20.5) | 51.0 (50.5 - 51.6) | 75.3 (74.7 - 75.9) | 14.4 (14.1 - 14.8) | 36.7 (36.1 - 37.4) | 66.7 (66.1 - 67.4) |  |
| Colombia | 49.9 (47.5 - 52.3) | 188.3 (182.7 - 194.5) | 277.8 (272.1 - 284.7) | 37.4 (35 - 39.8) | 102.7 (99.3 - 106.1) | 176.2 (174.9 - 177.6) | 30.8 (27.6 - 34.4) | 87.6 (77.6 – 98.0) | 133.8 (119.8 - 148.4) |  |
| Costa Rica | 24.4 (23.9 – 25.0) | 58.9 (57.5 - 60.2) | 83.6 (81.7 - 85.4) | 24.3 (23.7 - 24.9) | 58.6 (57.3 – 60.0) | 86.6 (85.0 - 88.4) | 25.6 (24.7 - 26.4) | 54.2 (52.5 - 55.8) | 109.9 (107.2 - 112.7) |  |
| Czechia | 18.5 (18.0 – 19.0) | 50.2 (49.2 - 51.2) | 71.6 (70.8 - 72.4) | 13.2 (12.9 - 13.6) | 37.0 (36.3 - 37.8) | 56.1 (55.2 - 57.1) | 10.3 (9.9 - 10.7) | 24.9 (24.2 - 25.6) | 43.8 (42.9 - 44.7) |  |
| Denmark | 15.2 (14.8 - 15.6) | 43.8 (43.1 - 44.5) | 53.1 (52.5 - 53.6) | 7.3 (7.0 - 7.6) | 24.5 (23.9 – 25.0) | 37.3 (36.9 - 37.8) | 6.5 (6.2 - 6.8) | 17.3 (16.8 - 17.9) | 31.4 (30.8 – 32.0) |  |
| Estonia | 26.9 (26.1 - 27.9) | 79.4 (77.4 - 81.7) | 148.3 (145.1 - 151.6) | 16.1 (15.6 - 16.8) | 43.9 (42.5 - 45.2) | 83.3 (81.0 - 85.6) | 12.7 (12.0 - 13.5) | 47.3 (45.4 - 49.4) | 57.7 (55.8 – 60.0) |  |
| Finland | 14.1 (13.7 - 14.6) | 49.5 (48.4 - 50.5) | 76.4 (75.5 - 77.1) | 10.0 (9.6 - 10.4) | 37.2 (36.3 - 38.1) | 68.6 (67.6 - 69.5) | 9.5 (9.0 – 10.0) | 36.0 (34.6 - 37.4) | 54.2 (53.1 - 55.2) |  |
| France | 15.4 (15.2 - 15.6) | 47.8 (47.5 - 48.2) | 69.3 (69.0 - 69.6) | 9.7 (9.6 - 9.9) | 30.6 (30.3 - 30.8) | 48.5 (48.3 - 48.7) | 7.5 (7.4 - 7.6) | 19.2 (18.9 - 19.4) | 38.0 (37.7 - 38.3) |  |
| Germany | 13.2 (13.1 - 13.4) | 45.5 (45.2 - 45.8) | 60.1 (59.9 - 60.3) | 9.6 (9.4 - 9.7) | 27.5 (27.2 - 27.7) | 37.7 (37.6 - 37.8) | 7.9 (7.8 – 8.0) | 22.3 (22.1 - 22.6) | 28.3 (28.1 - 28.5) |  |
| Greece | 13.8 (13.4 - 14.1) | 48.2 (47.5 - 48.9) | 73.2 (72.6 - 73.9) | 12.2 (11.9 - 12.5) | 37.0 (36.4 - 37.6) | 58.4 (57.8 – 59.0) | 8.0 (7.5 - 8.4) | 25.9 (24.9 - 26.8) | 44.2 (42.8 - 45.4) |  |
| Hungary | 20.7 (20.3 - 21.2) | 41.5 (40.9 - 42.1) | 62.8 (62.2 - 63.3) | 14.0 (13.7 - 14.4) | 33.4 (32.9 - 33.9) | 45.4 (45.0 - 45.9) | 8.0 (7.8 - 8.3) | 30.7 (30.1 - 31.5) | 52.6 (51.9 - 53.2) |  |
| Iceland | 10.0 (9.4 - 10.6) | 47.9 (45.8 - 50.1) | 71.9 (69.4 - 74.5) | 11.6 (11.0 - 12.4) | 29.9 (28.5 - 31.6) | 41.8 (39.7 - 43.8) | 14.4 (14.0 - 14.7) | 23.7 (21.5 - 26.1) | 34.0 (32.0 - 36.3) |  |
| Ireland | 16.7 (16.2 - 17.1) | 56.9 (55.8 - 57.9) | 80.6 (79.5 - 81.7) | 9.6 (9.3 - 9.9) | 37.8 (37.1 - 38.6) | 55.8 (54.9 - 56.5) | 4.9 (4.6 - 5.1) | 18.6 (17.9 - 19.2) | 27.2 (26.7 - 27.8) |  |
| Israel | 15.8 (15.5 - 16.1) | 47.5 (46.8 - 48.1) | 60.5 (59.9 - 61.2) | 11.9 (11.6 - 12.1) | 27.5 (27.1 - 27.9) | 41.3 (40.8 - 41.7) | 8.8 (8.4 - 9.1) | 30.6 (30.0 - 31.2) | 41.4 (40.7 - 42.2) |  |
| Italy | 15.0 (14.8 - 15.2) | 42.1 (41.8 - 42.4) | 61.4 (61.1 - 61.6) | 10.5 (10.3 - 10.6) | 28.1 (27.8 - 28.3) | 39.1 (38.9 - 39.3) | 8.0 (7.8 - 8.1) | 19.6 (19.4 - 19.9) | 25.9 (25.7 - 26.1) |  |
| Japan | 11.4 (11.3 - 11.5) | 32.4 (32.2 - 32.6) | 47.4 (47.2 - 47.6) | 8.8 (8.7 - 8.9) | 23.4 (23.2 - 23.6) | 43.0 (42.9 - 43.2) | 7.9 (7.8 – 8.0) | 20.8 (20.6 – 21.0) | 35.3 (35.1 - 35.4) |  |
| Latvia | 29.8 (29.1 - 30.6) | 82.8 (81.2 - 84.5) | 154.0 (151.3 - 156.7) | 24.2 (23.6 – 25.0) | 51.5 (50.2 - 52.7) | 81.5 (79.8 - 83.2) | 8.5 (8.1 - 8.8) | 31.7 (30.6 - 32.7) | 82.9 (80.8 – 85.0) |  |
| Lithuania | 28.0 (27.3 - 28.6) | 88.6 (87.0 - 90.1) | 152.6 (150.2 - 154.9) | 21.1 (20.5 - 21.8) | 66.2 (64.8 - 67.6) | 113.9 (112.0 - 115.7) | 15.9 (15.2 - 16.6) | 34.6 (33.5 - 35.7) | 68.5 (67.0 – 70.0) |  |
| Luxembourg | 10.3 (9.8 - 10.8) | 41.5 (39.8 - 43.3) | 70.1 (67.6 - 72.6) | 6.7 (6.2 - 7.1) | 24.5 (23.2 - 25.8) | 43.7 (42.0 - 45.4) | 5.9 (5.4 - 6.4) | 24.2 (22.4 - 26.1) | 28.7 (27.1 - 30.4) |  |
| Mexico | 38.3 (36.8 – 40.0) | 72.1 (70.5 - 73.8) | 103.7 (103.3 - 104.1) | 34.8 (32.8 - 36.8) | 87.6 (84.9 - 90.2) | 125.2 (124.8 - 125.7) | 31 (29.0 - 33.3) | 84.8 (81.9 – 88.0) | 148.7 (148.1 - 149.3) |  |
| Netherlands | 14.9 (14.6 - 15.2) | 37.3 (36.9 - 37.8) | 47.5 (47.2 - 47.9) | 10.0 (9.8 - 10.2) | 21.7 (21.4 – 22.0) | 31.1 (30.8 - 31.4) | 8.5 (8.2 - 8.7) | 16.4 (16.1 - 16.8) | 32.3 (31.9 - 32.7) |  |
| New Zealand | 22.6 (21.9 - 23.2) | 68.0 (66.9 - 69.2) | 81.4 (80.4 - 82.4) | 15.4 (15 - 15.9) | 54.7 (53.7 - 55.7) | 65.0 (64.3 - 65.8) | 11.4 (11.0 - 11.7) | 38.6 (37.7 - 39.4) | 50.6 (49.9 - 51.3) |  |
| Norway | 11.6 (11.3 – 12.0) | 53.5 (52.5 - 54.4) | 72.5 (71.6 - 73.3) | 10.1 (9.7 - 10.4) | 37.2 (36.5 - 37.9) | 54.0 (53.3 - 54.6) | 10.4 (10 - 10.9) | 26.7 (26.0 - 27.5) | 33.6 (33.0 - 34.1) |  |
| Poland | 19.4 (19.1 - 19.6) | 52.1 (51.7 - 52.5) | 77.7 (77.3 - 78.1) | 15.5 (15.3 - 15.7) | 45.5 (45.1 - 45.9) | 66.3 (66.0 - 66.8) | 10.7 (10.5 - 10.9) | 36.9 (36.4 - 37.3) | 59.1 (58.7 - 59.5) |  |
| Portugal | 23.5 (23.0 - 24.1) | 60.5 (59.6 - 61.3) | 91.5 (90.8 - 92.2) | 12.1 (11.8 - 12.5) | 28.4 (28.0 - 28.9) | 48.4 (47.9 - 48.9) | 9.8 (9.4 - 10.2) | 22.0 (21.3 - 22.6) | 31.7 (31.3 - 32.1) |  |
| Republic of Korea | 19.7 (19.4 - 19.9) | 46.4 (46.1 - 46.8) | 62.9 (62.6 - 63.2) | 13.0 (12.8 - 13.1) | 29.6 (29.4 - 29.9) | 43.6 (43.4 - 43.8) | 8.6 (8.1 - 9.1) | 22.0 (21.1 - 22.9) | 33.1 (32.3 - 33.8) |  |
| Slovakia | 23.1 (22.4 - 23.8) | 44.0 (43.1 – 45.0) | 68.0 (67.1 – 69.0) | 18.2 (17.8 - 18.7) | 35.8 (35.2 - 36.6) | 56.4 (55.5 - 57.3) | 12.0 (11.5 - 12.5) | 33.5 (32.6 - 34.4) | 48.3 (47.5 - 49.1) |  |
| Slovenia | 15.3 (14.7 - 15.8) | 56.3 (54.7 - 57.9) | 78.0 (76.1 - 79.8) | 9.3 (8.9 - 9.8) | 30.4 (29.3 - 31.5) | 49.8 (48.6 - 51.2) | 7.3 (6.9 - 7.8) | 28.6 (27.0 - 30.1) | 28.1 (27.0 - 29.3) |  |
| Spain | 16.6 (16.4 - 16.9) | 44.4 (44.0 - 44.8) | 58.8 (58.5 - 59.2) | 10.6 (10.5 - 10.8) | 23.3 (23.1 - 23.5) | 31.1 (30.9 - 31.2) | 8.6 (8.5 - 8.8) | 18.1 (17.8 - 18.4) | 30.2 (29.9 - 30.4) |  |
| Sweden | 12.1 (11.8 - 12.5) | 32.3 (31.8 - 32.8) | 49.9 (49.4 - 50.4) | 9.6 (9.3 - 9.9) | 28.0 (27.6 - 28.5) | 44.4 (44.0 - 44.8) | 7.6 (7.1 - 8.1) | 20.3 (18.6 - 21.9) | 40.8 (37.4 – 44.0) |  |
| Switzerland | 15.9 (15.5 - 16.3) | 40.5 (39.8 - 41.1) | 63.6 (63.1 - 64.3) | 8.9 (8.7 - 9.2) | 27.7 (27.2 - 28.2) | 32.4 (32.1 - 32.8) | 7.6 (7.3 - 7.9) | 20.7 (20.2 - 21.3) | 26.1 (25.7 - 26.6) |  |
| Türkiye | 42.8 (39.6 - 46.2) | 73.3 (66.2 - 81.2) | 79.8 (72.0 - 88.9) | 30.3 (27.9 - 32.9) | 56 (51.1 - 61.3) | 59.9 (54.8 - 65.2) | 22.7 (20.4 – 25.0) | 49.5 (42.5 – 57.0) | 59.4 (49.8 - 69.9) |  |
| United Kingdom | 14.2 (13.8 - 14.5) | 41.4 (40.8 - 41.9) | 58.2 (57.9 - 58.4) | 9.9 (9.8 - 10.1) | 29.5 (29.3 - 29.8) | 42.6 (42.4 - 42.8) | 8.8 (8.7 – 9.0) | 25.3 (25.1 - 25.5) | 40.9 (40.6 - 41.2) |  |
| United States of America | 19.9 (19.9 - 20.1) | 67.4 (67.2 - 67.6) | 92.9 (92.7 - 93.1) | 14.4 (14.3 - 14.4) | 50.5 (50.3 - 50.7) | 86.7 (86.5 - 86.9) | 16.0 (15.6 - 16.5) | 57.7 (56.8 - 58.7) | 110.6 (109.4 - 111.7) |  |
| *UI: uncertainty intervals | | | | | | | | | | |

#### Table S3: Number of mortalities/deaths among adolescents and young adults aged 10-24 years in 2000, 2010, and 2021, stratified by gender

| Country | Number of deaths (95% UI) | | | | | | | |
| --- | --- | --- | --- | --- | --- | --- | --- | --- |
|  | Females | | | | Males | | | |
|  | 2000 | 2010 | 2021 | 2000 | | 2010 | 2021 |  |
| *All OECD countries average* | *40731.5 (40350.3 - 41152.1)* | *33508.9 (33890.9 - 33187.6)* | *33312.8 (32774.2 - 33852.6)* | *108704.1 (107609.4 - 109927.4)* | | *88628.2 (89711.7 - 87547.7)* | *85358.0 (83691.4 - 87247.4)* |  |
| Australia | 525.9 (520.2 - 531.7) | 424.0 (428.9 - 419.3) | 392.5 (387.0 - 398.2) | 1335.5 (1322.9 - 1349.6) | | 970.6 (980.3 – 961.0) | 927.8 (915.3 - 939.5) |  |
| Austria | 178.4 (176.0 – 181.0) | 133.9 (135.8 – 132.0) | 77.6 (76.0 - 79.4) | 487.2 (481.4 - 493.1) | | 378.0 (383.2 - 373.2) | 261.8 (256.5 - 267.4) |  |
| Belgium | 259.7 (256.3 – 263.0) | 181.9 (184.5 - 179.5) | 149.8 (146.4 - 153.5) | 674.3 (666.1 - 681.5) | | 432.1 (437.2 - 427.3) | 301.7 (293.6 - 309.8) |  |
| Canada | 801.7 (795.6 – 808.0) | 717.2 (724.7 - 710.7) | 746.1 (733.9 - 758.4) | 1856.8 (1841.1 - 1871.5) | | 1595.9 (1608.4 - 1583.1) | 1564.1 (1525.0 - 1602.1) |  |
| Chile | 523.4 (518.1 - 529.4) | 601.7 (610.8 - 593.2) | 468.8 (462.8 - 475.8) | 1433.5 (1420.0 - 1447.8) | | 1477.6 (1493.2 – 1462.0) | 1105.8 (1089.8 - 1120.6) |  |
| Colombia | 3808.9 (3728.2 - 3900.8) | 2874.6 (2944.1 - 2808.6) | 2480.9 (2223.9 - 2763.1) | 15432.3 (15068.5 - 15859.7) | | 9975.3 (10167.0 - 9768.1) | 7932.7 (7011.6 - 8937.4) |  |
| Costa Rica | 190.0 (184.8 - 195.3) | 198.3 (204.1 - 192.9) | 183.5 (177.9 - 189.1) | 448.9 (436.4 - 460.5) | | 514.5 (527.8 - 501.4) | 519.8 (505.8 - 533.1) |  |
| Czechia | 293.7 (286.7 - 301.2) | 178.8 (182.9 - 175.1) | 116.1 (113.1 - 119.4) | 781.2 (770.5 - 792.8) | | 475.9 (483.4 - 468.6) | 274.2 (268.4 - 280.3) |  |
| Denmark | 95.6 (94.1 - 97.2) | 75.7 (77.3 – 74.0) | 60.4 (58.8 – 62.0) | 253.2 (249.3 – 257.0) | | 157.0 (159.7 - 154.4) | 137.7 (134.4 - 141.2) |  |
| Estonia | 57.0 (54.8 - 59.2) | 31.2 (32.7 - 29.7) | 28.7 (27.2 - 30.4) | 196.5 (191.5 - 201.9) | | 97.3 (100.5 - 94.2) | 47.2 (45.1 - 49.3) |  |
| Finland | 127.0 (124.1 - 130.1) | 102.3 (104.6 - 99.9) | 83.5 (80.9 – 86.0) | 332.0 (326.9 - 337.1) | | 275.9 (280.5 - 271.3) | 218.5 (212.4 - 224.9) |  |
| France | 1444.4 (1434.2 - 1455.3) | 957.3 (965.0 – 950.0) | 763.8 (756.4 - 770.8) | 3738.6 (3716.7 - 3762.2) | | 2549.4 (2564.8 – 2534.0) | 1832.0 (1815.5 - 1849.7) |  |
| Germany | 1570.4 (1560.1 – 1581.0) | 1054.5 (1061 - 1048.2) | 797.3 (788.9 - 806.2) | 3957.0 (3936.1 - 3977.5) | | 2346.4 (2362.4 - 2331.3) | 1746.7 (1730.6 - 1762.4) |  |
| Greece | 247.8 (244.4 - 251.7) | 175.7 (178.3 - 173.1) | 99.6 (96.0 - 102.6) | 819.7 (811.0 - 829.8) | | 492.5 (498.1 - 486.6) | 299.1 (288.2 - 307.9) |  |
| Hungary | 265.8 (261.3 - 270.2) | 161.2 (164.3 - 158.2) | 144.7 (141.3 - 147.7) | 675.7 (667.6 - 683.6) | | 394.3 (400.1 – 389.0) | 300.6 (295.6 - 306.6) |  |
| Iceland | 8.3 (7.9 - 8.6) | 6.8 (7.1 - 6.4) | 4.8 (4.6 - 4.9) | 19.4 (18.6 - 20.4) | | 12.5 (13.1 - 11.9) | 11.6 (10.7 - 12.5) |  |
| Ireland | 135.1 (132.7 - 137.4) | 80.9 (82.6 - 79.4) | 46.3 (45.0 - 47.7) | 362.7 (356.7 - 368.7) | | 230.1 (234.4 - 225.9) | 113.0 (109.6 - 116.6) |  |
| Israel | 168.7 (166.4 - 171.2) | 128.0 (130.0 - 125.8) | 137.2 (133.7 - 140.9) | 517.6 (511.0 - 524.1) | | 366.2 (371.5 - 361.2) | 448.6 (441.5 - 456.5) |  |
| Italy | 1005.3 (997.8 - 1012.9) | 654.2 (660.1 - 648.9) | 485.4 (480.6 - 490.4) | 2880.3 (2864.2 - 2897.6) | | 1688.2 (1698.6 - 1676.5) | 1075.5 (1065.3 - 1086.5) |  |
| Japan | 2171.2 (2158.0 - 2184.4) | 1540.3 (1549.9 - 1529.8) | 1392.5 (1381.7 – 1405.0) | 5173.8 (5146.9 - 5199.9) | | 3315.1 (3333.5 - 3296.7) | 2436.7 (2416.5 - 2458.3) |  |
| Latvia | 111.2 (108.1 - 114.5) | 56.5 (58.3 - 54.5) | 31.2 (29.9 - 32.5) | 340.5 (334.3 - 347.5) | | 169.6 (173.4 - 165.6) | 73.1 (71.0 - 75.3) |  |
| Lithuania | 150.9 (146.7 - 154.9) | 85.0 (87.6 - 82.5) | 43.1 (41.6 - 44.7) | 523.5 (514.7 - 532.9) | | 355.2 (362.3 - 348.4) | 125.6 (122.3 - 128.7) |  |
| Luxembourg | 8.8 (8.5 - 9.1) | 6.5 (6.8 - 6.2) | 7.9 (7.4 - 8.4) | 22.0 (21.1 – 23.0) | | 15.9 (16.6 - 15.1) | 13.5 (12.5 - 14.4) |  |
| Mexico | 6679.3 (6554.4 - 6807.4) | 7323.0 (7489.0 - 7160.5) | 7845.7 (7682.8 - 8025.3) | 15295.7 (15056.3 – 15549.0) | | 19218.2 (19570.9 - 18864.5) | 21142.2 (20767.4 - 21548.9) |  |
| Netherlands | 298.7 (294.7 - 302.4) | 196.7 (199.1 - 194.3) | 199.4 (196.2 - 202.6) | 647.0 (639.9 - 654.1) | | 438.5 (444.1 – 433.0) | 389.3 (382.2 - 396.5) |  |
| New Zealand | 140.0 (137.8 - 142.3) | 131.2 (133.5 - 129) | 128.7 (126.1 - 131.2) | 322.6 (317.7 - 328.1) | | 294.9 (300.2 - 289.7) | 212.7 (208.1 - 217.3) |  |
| Norway | 104.3 (102.5 - 106.2) | 102.0 (103.9 - 99.9) | 86.3 (84.2 - 88.4) | 272.0 (267.7 - 276.6) | | 215.0 (218.5 - 211.3) | 147.2 (144.1 - 150.8) |  |
| Poland | 1164.2 (1153.7 - 1176.7) | 734 (742.1 - 726.1) | 578.1 (571.5 - 584.8) | 3584.6 (3558.2 - 3609.7) | | 2618.5 (2637.9 - 2599.4) | 1461.0 (1447.0 - 1473.9) |  |
| Portugal | 331.8 (328.0 - 335.9) | 171.5 (174.1 - 169.1) | 98.3 (96.2 - 100.4) | 1021.6 (1010.0 - 1032.1) | | 359.7 (364.2 - 355.1) | 257.3 (251.3 - 263.6) |  |
| Republic of Korea | 1544.6 (1530.3 - 1558.8) | 1003.5 (1013.5 - 993.9) | 661.3 (641.7 - 682.9) | 3291.9 (3270.8 - 3312.7) | | 1864.1 (1878.8 - 1850.6) | 1063.9 (1030.1 - 1098.8) |  |
| Slovakia | 161.8 (157.2 - 166.6) | 108.3 (111.2 - 105.6) | 80.3 (78.1 - 82.7) | 450.5 (443.1 - 458.4) | | 304.6 (310.0 - 299.7) | 178.9 (174.7 - 182.8) |  |
| Slovenia | 52.1 (50.0 – 54.0) | 24.5 (25.6 - 23.5) | 17.9 (16.8 - 19.1) | 164.4 (159.8 - 169.3) | | 81.2 (84.2 - 78.5) | 43.3 (41.3 - 45.4) |  |
| Spain | 921.2 (912.7 - 930.4) | 486.4 (491.4 - 481.8) | 409.5 (404.4 - 414.8) | 2602.3 (2584.2 - 2621.8) | | 1112.5 (1121.0 - 1103.5) | 892.4 (881.1 - 904.5) |  |
| Sweden | 144.0 (141.9 - 146.3) | 152.8 (155.0 - 150.4) | 115.6 (105.1 – 127.0) | 350.0 (345.4 – 354.0) | | 347.1 (351.7 - 342.7) | 278.9 (252.4 - 307.1) |  |
| Switzerland | 145.5 (143.5 - 147.5) | 97.9 (99.5 – 96.0) | 78.9 (77.2 - 80.7) | 367.9 (363.0 - 372.8) | | 227.3 (231.1 – 224.0) | 171.7 (167.6 - 175.9) |  |
| Türkiye | 3808.1 (3475.5 - 4165.5) | 2417.9 (2673.0 - 2193.2) | 2548.1 (2230.5 - 2887.1) | 9503.0 (8560.4 - 10562) | | 6944.0 (7621.8 - 6226.9) | 5902.5 (4989.0 - 6868.6) |  |
| United Kingdom | 1229.4 (1216.2 - 1242.7) | 1049.6 (1057.5 - 1041.6) | 942.6 (933.4 - 952.8) | 2883.0 (2857.1 - 2904.4) | | 2317.4 (2332.0 - 2303.4) | 2082.7 (2063.2 - 2101.1) |  |
| United States of America | 9857.2 (9823.8 - 9890.9) | 9083.2 (9116.8 - 9053.1) | 10780.6 (10614.6 - 10934.1) | 25685.4 (25608.1 - 25766.1) | | 23999.4 (24080.4 - 23917.9) | 29366.6 (28952.8 - 29819.3) |  |
| *UI: uncertainty intervals | | | | | | | | |

#### Table S4: Rate of mortality among adolescents and young adults aged 10-24 years in 2000, 2010, and 2021, stratified by gender

| Country | Mortality Rate per 100,000 population (95% UI) | | | | | |
| --- | --- | --- | --- | --- | --- | --- |
|  | Females | | | Males | | |
|  | 2000 | 2010 | 2021 | 2000 | 2010 | 2021 |
| *All OECD countries average* | *31.5 (31.2 - 31.8)* | *26.2 (26.0 - 26.5)* | *26.8 (26.4 - 27.3)* | *80.8 (80.0 - 81.8)* | *66.6 (65.8 - 67.5)* | *65.6 (64.3 - 67.1)* |
| Australia | 27.2 (26.9 - 27.5) | 20.1 (19.9 - 20.3) | 17.1 (16.8 - 17.3) | 66.1 (65.5 - 66.8) | 44.0 (43.5 - 44.4) | 38.3 (37.8 - 38.7) |
| Austria | 25.7 (25.3 - 26.1) | 18.8 (18.5 – 19.0) | 11.4 (11.2 - 11.7) | 67.1 (66.3 – 68.0) | 50.9 (50.2 - 51.6) | 35.6 (34.9 - 36.4) |
| Belgium | 28.5 (28.2 - 28.9) | 19.2 (18.9 - 19.5) | 15.6 (15.2 – 16.0) | 71.4 (70.5 - 72.1) | 44.3 (43.8 - 44.8) | 30.1 (29.3 - 30.9) |
| Canada | 26.6 (26.4 - 26.8) | 22.8 (22.6 – 23.0) | 23.8 (23.4 - 24.2) | 59.4 (58.9 - 59.8) | 48.6 (48.2 – 49.0) | 46.9 (45.8 - 48.1) |
| Chile | 27.3 (27.1 - 27.6) | 29.3 (28.9 - 29.8) | 24.5 (24.2 - 24.9) | 72.5 (71.8 - 73.2) | 69.8 (69.1 - 70.6) | 54.9 (54.1 - 55.7) |
| Colombia | 65.3 (63.9 - 66.9) | 46.4 (45.3 - 47.5) | 42.5 (38.1 - 47.3) | 267.8 (261.5 - 275.2) | 157.3 (154.0 - 160.3) | 129.4 (114.3 - 145.7) |
| Costa Rica | 32.2 (31.3 - 33.1) | 31.7 (30.9 - 32.7) | 33.6 (32.5 - 34.6) | 74.1 (72.0 – 76.0) | 82.2 (80.1 - 84.3) | 95.0 (92.5 - 97.5) |
| Czechia | 27.4 (26.8 - 28.1) | 21.5 (21.0 – 22.0) | 15.3 (14.9 - 15.7) | 69.6 (68.7 - 70.7) | 54.3 (53.5 - 55.1) | 34.2 (33.5 – 35.0) |
| Denmark | 21.2 (20.9 - 21.6) | 15.2 (14.8 - 15.5) | 11.8 (11.5 - 12.1) | 54.0 (53.2 - 54.8) | 30.0 (29.5 - 30.5) | 25.6 (25.0 - 26.2) |
| Estonia | 38.6 (37.1 – 40.0) | 26.7 (25.4 – 28.0) | 29.1 (27.6 - 30.9) | 125.4 (122.2 - 128.9) | 78.4 (75.9 – 81.0) | 45.5 (43.5 - 47.5) |
| Finland | 26.6 (26.0 - 27.2) | 21.8 (21.3 - 22.3) | 18.7 (18.1 - 19.2) | 66.5 (65.4 - 67.5) | 56.5 (55.5 - 57.4) | 46.5 (45.2 - 47.8) |
| France | 24.9 (24.8 - 25.1) | 16.5 (16.4 - 16.7) | 12.8 (12.7 - 12.9) | 62.3 (61.9 - 62.7) | 42.4 (42.2 - 42.7) | 29.4 (29.1 - 29.6) |
| Germany | 22.9 (22.8 - 23.1) | 16.5 (16.4 - 16.6) | 13.3 (13.2 - 13.5) | 55.1 (54.8 - 55.4) | 34.9 (34.7 - 35.2) | 26.3 (26.0 - 26.5) |
| Greece | 23.2 (22.9 - 23.5) | 20.4 (20.1 - 20.7) | 13.1 (12.7 - 13.5) | 70.4 (69.6 - 71.2) | 53.3 (52.7 - 53.9) | 38.1 (36.7 - 39.2) |
| Hungary | 25.3 (24.8 - 25.7) | 19.1 (18.7 - 19.5) | 20.5 (20.0 - 20.9) | 61.4 (60.7 - 62.1) | 44.4 (43.8 - 45.1) | 40.3 (39.6 - 41.1) |
| Iceland | 26.4 (25.2 - 27.5) | 20.1 (19.1 - 21.1) | 14.3 (13.9 - 14.7) | 59.9 (57.3 - 62.7) | 35.8 (33.9 - 37.5) | 33.1 (30.7 - 35.9) |
| Ireland | 28.9 (28.3 - 29.3) | 18.2 (17.8 - 18.5) | 9.6 (9.3 - 9.9) | 74.4 (73.2 - 75.6) | 50.5 (49.6 - 51.5) | 22.6 (21.9 - 23.3) |
| Israel | 20.6 (20.3 - 20.9) | 13.9 (13.7 - 14.1) | 12.5 (12.1 - 12.8) | 60.1 (59.3 - 60.8) | 38.1 (37.6 - 38.7) | 38.9 (38.3 - 39.6) |
| Italy | 21.9 (21.7 – 22.0) | 15.1 (15.0 - 15.3) | 11.6 (11.5 - 11.7) | 60.1 (59.8 - 60.5) | 37.1 (36.8 - 37.3) | 23.9 (23.6 - 24.1) |
| Japan | 19.3 (19.2 - 19.5) | 16.6 (16.5 - 16.7) | 16.3 (16.1 - 16.4) | 43.9 (43.7 - 44.1) | 34.1 (33.9 - 34.3) | 27.0 (26.8 - 27.3) |
| Latvia | 43.1 (41.9 - 44.4) | 29.5 (28.5 - 30.5) | 23.6 (22.7 - 24.6) | 126.9 (124.6 - 129.5) | 84.6 (82.5 - 86.5) | 52.5 (51.0 - 54.1) |
| Lithuania | 39.4 (38.3 - 40.5) | 27.9 (27.1 - 28.8) | 21.5 (20.7 - 22.2) | 132.3 (130.1 - 134.6) | 110.9 (108.8 - 113.2) | 59.3 (57.8 - 60.8) |
| Luxembourg | 23.5 (22.7 - 24.4) | 14.7 (14.0 - 15.4) | 15.3 (14.4 - 16.3) | 57.1 (54.6 - 59.7) | 34.5 (32.8 - 36.1) | 24.3 (22.7 - 26.1) |
| Mexico | 41.8 (41.0 - 42.6) | 44.6 (43.6 - 45.6) | 47.5 (46.6 - 48.6) | 98.4 (96.9 - 100.1) | 117.7 (115.6 - 119.9) | 126.7 (124.5 - 129.2) |
| Netherlands | 21.4 (21.1 - 21.6) | 13.3 (13.1 - 13.4) | 13.6 (13.3 - 13.8) | 44.6 (44.1 - 45.1) | 28.5 (28.2 - 28.9) | 25.3 (24.8 - 25.8) |
| New Zealand | 34.5 (33.9 – 35.0) | 28.5 (28.0 – 29.0) | 26.4 (25.8 - 26.9) | 76.9 (75.7 - 78.2) | 62.1 (61.0 - 63.2) | 40.6 (39.7 - 41.4) |
| Norway | 25.8 (25.3 - 26.3) | 22.2 (21.7 - 22.6) | 18.0 (17.6 - 18.5) | 64.4 (63.4 - 65.5) | 44.4 (43.7 - 45.2) | 28.9 (28.3 - 29.6) |
| Poland | 25.2 (25.0 - 25.5) | 20.4 (20.1 - 20.6) | 20.4 (20.1 - 20.6) | 74.6 (74.1 - 75.1) | 69.6 (69.1 - 70.1) | 49.0 (48.5 - 49.4) |
| Portugal | 31.0 (30.7 - 31.4) | 19.7 (19.4 – 20.0) | 12.2 (12.0 - 12.5) | 92.1 (91.0 – 93.0) | 40.0 (39.4 - 40.5) | 30.7 (30.0 - 31.5) |
| Republic of Korea | 30.0 (29.7 - 30.2) | 21.3 (21.1 - 21.5) | 17.7 (17.2 - 18.3) | 58.0 (57.6 - 58.4) | 35.4 (35.1 - 35.6) | 26.7 (25.9 - 27.6) |
| Slovakia | 24.9 (24.2 - 25.6) | 20.9 (20.4 - 21.5) | 19.7 (19.2 - 20.3) | 66.4 (65.4 - 67.6) | 56.3 (55.4 - 57.3) | 41.7 (40.7 - 42.6) |
| Slovenia | 25.6 (24.6 - 26.6) | 15.4 (14.7 – 16.0) | 12.5 (11.7 - 13.3) | 76.7 (74.6 – 79.0) | 47.7 (46.1 - 49.5) | 28.4 (27.1 - 29.8) |
| Spain | 23.0 (22.8 - 23.2) | 14.0 (13.8 - 14.1) | 12.0 (11.9 - 12.2) | 61.7 (61.3 - 62.2) | 30.4 (30.2 - 30.7) | 24.9 (24.6 - 25.2) |
| Sweden | 18.3 (18.0 - 18.6) | 18.1 (17.8 - 18.3) | 13.5 (12.3 - 14.9) | 42.3 (41.8 - 42.8) | 39.0 (38.5 - 39.5) | 30.3 (27.4 - 33.4) |
| Switzerland | 23.2 (22.9 - 23.5) | 14.6 (14.3 - 14.8) | 12.0 (11.8 - 12.3) | 56.1 (55.4 - 56.9) | 32.4 (32.0 – 33.0) | 24.4 (23.8 – 25.0) |
| Türkiye | 38.5 (35.2 - 42.2) | 25.7 (23.3 - 28.4) | 27.2 (23.8 - 30.8) | 90.3 (81.4 - 100.4) | 70.2 (62.9 – 77.0) | 59.8 (50.5 - 69.6) |
| United Kingdom | 22.6 (22.4 - 22.9) | 17.8 (17.7 – 18.0) | 15.8 (15.7 – 16.0) | 51.4 (50.9 - 51.8) | 38.0 (37.7 - 38.2) | 33.9 (33.6 - 34.2) |
| United States of America | 33.7 (33.6 - 33.8) | 28.7 (28.6 - 28.8) | 34.0 (33.5 - 34.5) | 83.4 (83.2 - 83.7) | 72.4 (72.2 - 72.6) | 88.8 (87.5 - 90.1) |
| *UI: uncertainty intervals | | | | | | |

#### Table S5: Number of DALYs among adolescents and young adults (both genders) in years 2000, 2010, and 2021, stratified by age

| Country | Number of DALYs (95% UI) | | | | | | | | |
| --- | --- | --- | --- | --- | --- | --- | --- | --- | --- |
|  | 2000 | | | 2010 | | | 2021 | | |
|  | 10-14 years | 15-19 years | 20-24 years | 10-14 years | 15-19 years | 20-24 years | 10-14 years | 15-19 years | 20-24 years |
| *All OECD countries average* | *6213768.8 (4940198.0 - 7803423.1)* | *10812510.7 (8938131.5 - 13050503.7)* | *13086584.0 (10870629.4 - 15523870.1)* | *5679674.5 (7212868.5 - 4420786.6)* | *9910860.3 (12119506.2 - 8051344.2)* | *12595208.6 (15079482.7 - 10310066.2)* | *5733944.3 (4436831.6 - 7291788.6)* | *9721886.8 (7841114.6 – 11982094.0)* | *13308460.7 (10894576.7 - 15918889.8)* |
| Australia | 90361.6 (70397.0 – 115160.0) | 169483.6 (138884.1 – 204824.0) | 208954.0 (170246.4 - 251836.9) | 88964.1 (115030.4 – 68410.0) | 164078.4 (201545.1 - 130383.8) | 213965.3 (261483.6 - 169485.8) | 100382.6 (76959.3 - 129413.7) | 161811.5 (128040.0 - 200294.7) | 227469.5 (179224.4 - 277355.6) |
| Austria | 30812.5 (23657.9 - 39848.2) | 58014.0 (47852.4 - 70648.3) | 66067.4 (53875.9 - 79529.2) | 27903.7 (36403.4 - 21078.8) | 52422.8 (65316.9 - 41811.3) | 68693.7 (83972.2 - 54879.7) | 27351.4 (19948.8 – 36424.0) | 46451.7 (35692.8 - 58866.2) | 63628.1 (49645.6 - 80059.6) |
| Belgium | 41764.9 (32012.2 - 54494.2) | 72293.1 (58805.7 - 89262.2) | 95648.6 (78423.9 - 115940.3) | 40138.1 (52940.5 - 29934.4) | 67777.3 (85509.6 - 53114.8) | 87622.5 (109857.3 - 69132.2) | 43896.9 (31981.9 - 58236.9) | 64041.7 (49190.0 – 81643.0) | 80216.3 (61622.3 - 101357.8) |
| Canada | 124403.4 (96639.0 - 158576.8) | 238450.2 (193435.7 - 292392.8) | 277764.0 (224679.1 - 336211.5) | 113755.4 (146955.8 - 86796.8) | 237463.8 (293105.2 - 189073.1) | 295932.7 (356953.9 - 236744.1) | 124556.8 (94833.7 - 161330.5) | 218700.4 (171737.5 - 270094.1) | 323707.4 (258079.0 - 390887.8) |
| Chile | 98889.2 (79266.7 - 123164.9) | 150986.1 (124433.3 - 181411.1) | 177788.6 (146369.8 - 210057.8) | 88405.7 (110723.9 - 70157.2) | 166495.1 (200286.8 - 135413.9) | 205949.0 (246141.2 - 168691.3) | 87059.0 (67358.0 - 109952.6) | 135829.0 (107748.2 - 167573.4) | 201845.0 (161508.5 - 243851.3) |
| Colombia | 384767.6 (322360.6 - 467822.8) | 820923.2 (742696.0 - 913433.5) | 971831.5 (886820.6 - 1064659.8) | 355316.2 (441350.2 - 289754.3) | 618051.2 (723535.4 - 536879.1) | 773654.4 (874713.1 - 686942.6) | 283334.6 (226912.0 - 355673.6) | 530891.1 (447542.1 – 624724.0) | 763597.1 (652971.0 - 888791.1) |
| Costa Rica | 31528.5 (24882.2 - 40216.8) | 44839.9 (37333.3 – 54419.0) | 48281.7 (40406.8 - 57061.1) | 29143.9 (37027.8 - 22943.6) | 47377.4 (57173.1 - 39282.5) | 59558.7 (70384.7 - 49982.1) | 26648.3 (20841.8 – 34035.0) | 39221.2 (32356.8 - 48223.2) | 61623.8 (52276.2 - 72687.3) |
| Czechia | 40480.2 (31555.5 - 51063.5) | 71553.3 (58883.8 - 87020.2) | 108361.9 (89034.5 - 129306.1) | 26302.4 (34023.2 - 20201.3) | 54887.3 (67281.2 - 44245.5) | 80100.7 (96564.6 - 64708.1) | 34357.5 (26524.4 - 44735.7) | 44649.5 (35234.2 – 56125.0) | 53056.3 (41808.2 – 65065.0) |
| Denmark | 20212.4 (15495.3 - 26151.7) | 31063.9 (25046.7 – 38173.0) | 43791.9 (34964.7 - 53590.3) | 21430.6 (28139.8 – 15924.0) | 34065.8 (43182.8 - 26429.1) | 38702.2 (48077.2 - 30035.3) | 21192.2 (15799.7 - 27894.1) | 31985.3 (24614.4 - 40829.1) | 43733.3 (34297.6 - 54860.4) |
| Estonia | 7225.8 (5817.2 - 8991.5) | 12727.9 (10834.3 - 14969.3) | 18196.0 (15829.3 - 20687.4) | 3548.6 (4546.1 - 2787.7) | 7668.5 (9315.7 - 6273.2) | 14023.6 (16511.8 - 11684.1) | 4450.4 (3465.6 - 5682.2) | 6674.1 (5485.7 - 8054.6) | 8055.7 (6518.6 - 9745.8) |
| Finland | 20878.5 (15896.9 - 27145.8) | 38184.9 (30963.9 - 47384.1) | 48937.2 (39949.4 - 59590.7) | 18479.5 (24256.0 - 13805.4) | 35449.9 (44347.6 - 28350.8) | 47834.1 (58348.2 - 38726) | 19887.7 (14832.0 - 26344.4) | 32636.1 (26097.9 - 40627.6) | 41615.9 (32802.2 - 51646.2) |
| France | 274270.7 (210878.1 - 352228.1) | 475072.2 (385252.9 - 585137.6) | 545276.3 (439474.4 - 665064.7) | 255920.0 (337488.2 - 192384.7) | 411126.7 (516782.1 – 322472.0) | 508093.2 (630381.8 - 400684.7) | 284233.2 (207024.5 - 377282.2) | 413533.9 (312888.0 - 528138.4) | 485430.7 (377378.3 - 607260.4) |
| Germany | 332418.5 (252451.0 - 432197.7) | 536450.3 (430315.8 - 662067.4) | 638409.8 (513100.7 - 786216.3) | 269592.4 (354640.2 - 202685.5) | 432448.9 (553747.4 - 333644.3) | 622459.4 (786564.3 - 484218.1) | 265943.4 (195280.0 - 354558.6) | 409700.3 (315593.4 - 524843.4) | 563296.5 (427235.1 – 717525.0) |
| Greece | 42722.1 (32547.6 - 55928.5) | 89624.3 (71768.3 - 110295.9) | 125331.0 (101145.3 - 152351.1) | 35904.0 (46781.9 – 27519.0) | 64271.5 (79902.0 – 50668.0) | 91679.2 (111934.1 - 72991.7) | 35908.0 (26456.4 - 47528.2) | 54438.3 (41435.6 - 68353.1) | 67379.5 (52080.6 – 83362.0) |
| Hungary | 41154.8 (32498.4 - 52353.2) | 67654.5 (55477.1 - 82699.1) | 102526.8 (84007.5 – 122786.0) | 30211.4 (38647.7 - 23500.3) | 56509.2 (69941.4 - 45703.8) | 67716.6 (82555.3 - 53990.5) | 27024.9 (20280.2 - 35189.4) | 44755.1 (35728.6 - 55568.5) | 57685.9 (46131.2 - 70403.7) |
| Iceland | 1369.2 (1031.3 - 1800.7) | 2509.0 (2030.9 - 3079.8) | 3000.8 (2454.0 – 3630.0) | 1448.0 (1890.8 - 1098.6) | 2424.3 (3055.1 - 1901.7) | 2805.6 (3503.8 - 2184.5) | 1626.5 (1245.9 - 2127.9) | 2117.9 (1637.7 - 2671.6) | 2721.5 (2115.9 - 3422.4) |
| Ireland | 21337.4 (16327.2 - 27400.5) | 41818.0 (34239.6 - 50809.2) | 49288.0 (40922.4 - 58836.2) | 20928.2 (27336.3 - 15697.1) | 33165.4 (41562.9 - 26252.2) | 43505.7 (53345.8 - 34714.5) | 23927.8 (17421.5 - 31582.6) | 33685.1 (25785.8 - 42979.3) | 38192.9 (29158.2 – 48267.0) |
| Israel | 37217.7 (28824.6 - 48154.1) | 61947.8 (50199.9 - 75661.3) | 68452.3 (54703.4 - 84286.3) | 41234.8 (53671.9 - 31335.6) | 58718.3 (73855.8 - 45738.5) | 68617.2 (85361.5 - 53781.9) | 49836.1 (36803.3 - 65724.8) | 74401.3 (58062.0 - 93737.9) | 81442.1 (63729.7 - 101901.3) |
| Italy | 188529.4 (144159.4 - 244995.8) | 337268.8 (268503.9 - 421137.4) | 492651.7 (395909.1 - 607533.7) | 177839.5 (233943.4 - 133791.5) | 298172.2 (378160.1 - 230861.1) | 377664.6 (477034.5 – 295733.0) | 187992.0 (139290.0 – 249221.0) | 284360.2 (216064.4 – 367841.0) | 340278.8 (259177.9 – 436520.0) |
| Japan | 387233.7 (298310.7 - 496851.2) | 685825.9 (552879.1 – 843035.0) | 931830.9 (752528.1 - 1133363.9) | 337985.2 (436511.5 - 256957.1) | 515794.7 (643312.8 - 408429.7) | 699357.2 (855757.9 - 561404.8) | 311700.2 (237912.6 - 403803.7) | 477474.9 (374369.1 - 600759.3) | 629731.0 (497251.4 - 782387.1) |
| Latvia | 12775.8 (10439.8 - 15878.1) | 22074.4 (18995.8 - 25812.6) | 29835.5 (26385.3 - 33943.3) | 5813.6 (7208.8 - 4706.6) | 13526.8 (16202.8 - 11240.3) | 21516.1 (25284.6 - 18067.3) | 5499.8 (4130.5 - 7141.6) | 8153.7 (6494.5 - 10191.3) | 11317.0 (9402.6 - 13490.9) |
| Lithuania | 18965.8 (15430.1 - 23602.4) | 33941.0 (29286.0 - 39389.7) | 43835.0 (38597.7 – 49730.0) | 11030.7 (13730.7 - 8798.8) | 25297.6 (29770.0 - 21447.3) | 34415.3 (39732.9 - 29610.4) | 8419.4 (6532.0 - 10799.6) | 12246.6 (9734.0 - 15126.9) | 20219.5 (16478.5 - 24328.3) |
| Luxembourg | 1653.1 (1244.1 - 2151.2) | 2731.5 (2193.1 - 3383.7) | 3737.2 (3031.9 - 4533.9) | 1856.9 (2453.7 - 1383.5) | 2906.8 (3700.1 - 2265.6) | 3715.9 (4610.0 - 2923.2) | 2146.7 (1595.1 - 2879.3) | 3383.7 (2619.2 - 4299.7) | 4571.2 (3459.1 - 5800.6) |
| Mexico | 907954.4 (745754.5 - 1118494.3) | 1260736.5 (1070779.1 - 1503722.7) | 1459564.5 (1245316.9 - 1703049.2) | 894632.9 (1102575.5 - 728681.2) | 1467242.4 (1724438.0 - 1263926.9) | 1719738.8 (1987404.4 - 1487269.7) | 843332.2 (678831.3 - 1048421.3) | 1455123.2 (1244831.1 - 1718005.6) | 2029705.5 (1759839.1 - 2326800.6) |
| Netherlands | 63197.3 (48198.9 - 81413.2) | 95725.1 (76049.1 - 117863.7) | 116197.3 (92833.2 - 142019.4) | 62795.8 (81930.3 - 46915.3) | 94824.7 (120694.2 - 72739.9) | 111203.5 (140571.2 – 86685.0) | 62238.9 (46192.8 - 81727.7) | 95688.6 (72262.0 - 123800.3) | 124668.1 (96709.3 - 158402.2) |
| New Zealand | 22785.6 (18019.8 - 28739.3) | 38417.4 (31859.1 - 45958.9) | 42816.1 (34972.1 – 51149.0) | 20994.5 (26387.7 - 16162.2) | 40744.7 (49138.4 - 33418.6) | 47714.1 (57513.5 - 38383.7) | 21998.3 (16816.7 - 28285.7) | 36955.2 (29319.4 - 45812.7) | 51055.7 (40124.8 - 62685.8) |
| Norway | 18526.6 (14039.9 - 23958.8) | 31213.7 (25454.7 - 37823.5) | 40392.9 (32887.6 - 48624.9) | 20684.3 (27234.8 - 15442.5) | 34754.4 (43643.1 - 27496.8) | 40605.9 (49883.2 - 32239.3) | 21728.5 (16279.8 - 28695.1) | 32108.0 (24948.3 - 40910.7) | 40752.1 (31429.6 - 50912.9) |
| Poland | 176007.7 (139146.5 - 223518.8) | 338723.0 (281926.9 - 408282.9) | 404775.9 (336069.7 - 481601.3) | 113674.9 (145818.0 – 88809.0) | 235871.6 (287488.9 - 193869.4) | 346745.4 (416654.5 - 285045.6) | 118363.7 (89594.1 - 154241.1) | 169281.6 (136048.9 - 208656.8) | 233900.5 (189748.1 - 283421.1) |
| Portugal | 47301.2 (36808.9 - 59875.3) | 95174.8 (77297.6 – 114852.0) | 135619.0 (112309.5 - 162383.7) | 40530.3 (52708.2 - 30719.7) | 63150.8 (79902.6 - 49182.4) | 80530.4 (100329.8 - 63549.4) | 36785.0 (27319.4 - 49266.9) | 59329.3 (45101.4 - 75403.7) | 72855.1 (55705.8 – 91276.0) |
| Republic of Korea | 200436.9 (158943.3 - 251975.7) | 379408.8 (312259.2 - 461224.7) | 474280.1 (393643.3 - 566518.7) | 189249.8 (242901.9 - 146429.3) | 308385.3 (382822.3 - 246576.9) | 339490.8 (414267.5 - 273658.9) | 124861.8 (94550.6 - 162120.5) | 190151.3 (148514.7 – 238539.0) | 307198.2 (240371.0 - 378985.2) |
| Slovakia | 27358.0 (21699.6 - 34209.9) | 44416.7 (36355.3 - 54137.8) | 59143.6 (48675.0 - 70397.8) | 17725.3 (22188.8 - 13866.3) | 32812 (40467.8 - 26438.5) | 47420.9 (57293.7 - 38580.8) | 17168.2 (13266.7 - 21981.9) | 24811.9 (19841.5 - 30732.6) | 32274.5 (25797.7 - 39583.8) |
| Slovenia | 7436.0 (5786.3 - 9470.2) | 15167.1 (12583.3 – 18281.0) | 20093.9 (16732.9 - 23758.6) | 5174.6 (6706.2 - 3928.6) | 9410.9 (11703.0 – 7522.0) | 14916.9 (18065.6 - 11973.7) | 5821.5 (4355.6 - 7668.9) | 8245.1 (6575.2 - 10292.1) | 9719.2 (7580.1 - 12097.1) |
| Spain | 145396.9 (112437.2 - 187330.7) | 291379.0 (234284.9 - 362707.2) | 427231.3 (343229.0 - 526701.3) | 138638.7 (180963.7 - 103590.9) | 218579.5 (279951.5 - 169693.2) | 298304.2 (380640.8 - 230398.2) | 164280.0 (122776.5 - 217518.3) | 225945.3 (173140.8 - 292719.9) | 258484.4 (195261.4 - 327699.1) |
| Sweden | 36968.7 (28087.4 - 47864.8) | 50696.6 (39978.0 - 64033.6) | 63887.5 (50722.7 - 79555.4) | 29622.3 (38781.5 - 22388.1) | 59952.6 (76499.9 - 47052.8) | 76360.8 (95054.0 - 60303.9) | 38911.2 (28946.9 - 50773.5) | 56476.7 (43481.9 - 72157.2) | 69122.3 (54331.2 - 86162.9) |
| Switzerland | 30968.6 (24055.7 – 39729.0) | 49214.3 (39146.5 - 60989.5) | 64931.0 (52082.5 - 79143.1) | 27621.0 (36232.4 - 20633.3) | 47758 (60256.1 - 37239.4) | 61192.9 (76590.2 - 47474.9) | 28999.9 (21533.6 - 38401.3) | 42926.7 (33054.6 - 54358.6) | 57165.4 (43703.1 - 72082.3) |
| Türkiye | 612478.0 (504834.1 - 750423.7) | 884125.1 (741126.6 - 1060864.9) | 927024.7 (779476.0 - 1107075.6) | 523731.2 (651980.8 - 417799.5) | 712821.5 (857718.7 - 588997.1) | 773676.8 (932042.2 - 632932.8) | 492285.6 (384356.2 - 627766.5) | 701039.9 (559225.7 - 854239.9) | 846120.2 (675493.2 - 1024503.5) |
| United Kingdom | 260252.0 (198501.2 - 334519.3) | 411556.2 (327937.9 - 510559.9) | 498267.3 (397806.1 - 609700.1) | 234190.7 (306221.8 - 175476.5) | 417774.2 (526234.7 - 326344.6) | 550490.7 (683821.3 - 430842.6) | 269969.1 (201613.1 - 354177.8) | 404090.7 (312922.4 - 512128.1) | 542850.5 (421584.0 - 675036.3) |
| United States of America | 1405728.1 (1111109.2 - 1765025.9) | 2761118.6 (2276470.8 - 3306646.6) | 3252560.7 (2672196.0 - 3864769.3) | 1357255.6 (1735342.8 - 1045388.3) | 2766677.9 (3368437.0 - 2230940.3) | 3659233.7 (4365674.9 - 2998191.4) | 1509824.9 (1167300.6 - 1919612.7) | 3088570.7 (2479864.8 - 3753000.7) | 4461774.1 (3683663.9 – 5290141.0) |
| *UI: uncertainty intervals | | | | | | | | | |

#### Table S6: Rate of DALYs among adolescents and young adults (both genders) in years 2000, 2010, and 2021, stratified by age

| Country | DALYs rate per 100,000 population  Rate/100k (95% UI) | | | | | | | | |
| --- | --- | --- | --- | --- | --- | --- | --- | --- | --- |
|  | 2000 | | | 2010 | | | 2021 | | |
|  | 10-14 years | 15-19 years | 20-24 years | 10-14 years | 15-19 years | 20-24 years | 10-14 years | 15-19 years | 20-24 years |
| *All OECD countries average* | *7116.1 (5657.6 - 8936.6)* | *12194.2 (10080.3 - 14718.1)* | *14878.2 (12358.8 - 17649.1)* | *6744.3 (5249.4 - 8564.9)* | *11286.4 (9168.8 - 13801.5)* | *14193.7 (11618.6 - 16993.3)* | *6842.1 (5294.3 – 8701.0)* | *11631.9 (9381.6 - 14336.1)* | *15319.0 (12,540.4 - 18,323.8)* |
| Australia | 6671.7 (5197.6 - 8502.6) | 12651.2 (10367.1 - 15289.2) | 16557.9 (13490.6 – 19956.0) | 6334.8 (4871.2 - 8190.9) | 11386.4 (9048.1 - 13986.4) | 14544.6 (11521.0 - 17774.7) | 6184.7 (4741.6 - 7973.4) | 10868.0 (8599.8 - 13452.7) | 14104.5 (11113.0 - 17197.7) |
| Austria | 6575.2 (5048.4 - 8503.3) | 11953.7 (9859.9 – 14557.0) | 14176.3 (11560.3 - 17064.8) | 6364.3 (4807.7 - 8302.9) | 10561.5 (8423.6 - 13159.2) | 13184.9 (10533.5 - 16117.5) | 6355.5 (4635.4 - 8463.6) | 10145.1 (7795.4 - 12856.5) | 12129.0 (9463.6 - 15261.2) |
| Belgium | 6803.9 (5215.1 - 8877.6) | 11864.0 (9650.6 - 14648.8) | 15149.4 (12421.2 - 18363.3) | 6599.8 (4922.1 - 8704.9) | 10402.2 (8151.9 - 13123.7) | 13215.2 (10426.5 - 16568.6) | 6535.3 (4761.4 - 8670.2) | 10130.9 (7781.5 - 12915.3) | 12143.2 (9328.4 - 15343.6) |
| Canada | 5981.5 (4646.5 - 7624.6) | 11413.9 (9259.2 - 13995.9) | 14059.6 (11372.6 – 17018.0) | 5698.8 (4348.3 – 7362.0) | 10613.7 (8450.8 - 13100.6) | 13456.6 (10765.2 - 16231.4) | 5733.5 (4365.3 - 7426.2) | 10631.7 (8348.7 - 13130.1) | 14479.5 (11543.9 - 17484.5) |
| Chile | 7009.8 (5618.9 - 8730.6) | 11568.1 (9533.7 - 13899.1) | 15114.2 (12443.3 - 17857.5) | 6917.4 (5489.5 - 8663.7) | 11415.1 (9284.1 - 13731.9) | 14386.9 (11784.2 - 17194.6) | 6778.0 (5244.2 - 8560.4) | 10937.3 (8676.1 - 13493.4) | 14438.0 (11552.7 - 17442.7) |
| Colombia | 9258.3 (7756.6 - 11256.8) | 21021.5 (19018.3 - 23390.4) | 27505.0 (25099.0 - 30132.3) | 8101.6 (6606.7 - 10063.3) | 14345.8 (12461.7 - 16794.2) | 20091.7 (17839.8 - 22716.2) | 7722.8 (6184.9 - 9694.5) | 13452.6 (11340.6 - 15830.3) | 17541.2 (14999.9 - 20417.1) |
| Costa Rica | 7138.4 (5633.6 - 9105.6) | 11127.7 (9264.8 - 13504.9) | 13736.9 (11496.4 - 16234.8) | 7185.9 (5657.1 - 9129.8) | 11217.3 (9300.7 - 13536.5) | 14069.5 (11807.2 - 16626.9) | 7421.5 (5804.4 - 9478.7) | 11152.5 (9200.6 - 13712.2) | 16095.8 (13654.3 - 18985.5) |
| Czechia | 6189.1 (4824.6 - 7807.2) | 10382.2 (8543.9 - 12626.4) | 12753.5 (10478.8 - 15218.5) | 5816.9 (4467.6 - 7524.4) | 9482.8 (7644.3 - 11624.1) | 11805.1 (9536.6 - 14231.6) | 5801.3 (4478.6 - 7553.6) | 8856.0 (6988.5 - 11132.1) | 11401.6 (8984.5 - 13982.3) |
| Denmark | 6759.0 (5181.6 - 8745.1) | 11044.1 (8904.9 - 13571.7) | 12901.2 (10300.7 - 15787.8) | 6204.0 (4609.9 - 8146.4) | 9706.2 (7530.3 - 12303.8) | 11884.8 (9223.3 - 14763.6) | 6288.1 (4688.0 - 8276.7) | 9406.2 (7238.6 – 12007.0) | 11722.9 (9193.6 - 14705.6) |
| Estonia | 6855.8 (5519.3 - 8531.1) | 12497.3 (10638.1 - 14698.2) | 18705.5 (16272.5 - 21266.7) | 5833.6 (4582.8 - 7473.4) | 9631.6 (7879.1 - 11700.5) | 13945.9 (11619.4 - 16420.3) | 5846.6 (4552.8 - 7464.8) | 10400.8 (8548.8 – 12552.0) | 13005.8 (10524.1 - 15734.4) |
| Finland | 6552.0 (4988.7 - 8518.8) | 11509.7 (9333.1 - 14282.5) | 14959.1 (12211.7 - 18215.6) | 6225.6 (4650.9 - 8171.6) | 10670.4 (8533.6 - 13348.6) | 14582.5 (11805.9 - 17787.8) | 6358.1 (4741.8 - 8422.3) | 10827.2 (8658.1 - 13478.4) | 13744.1 (10833.3 - 17056.7) |
| France | 6975.1 (5363.0 - 8957.7) | 11733.7 (9515.3 - 14452.2) | 14303.0 (11527.7 - 17445.1) | 6571.4 (4939.9 - 8665.8) | 10408.2 (8163.8 - 13083) | 12834.9 (10121.7 - 15924.0) | 6838.6 (4981.0 - 9077.4) | 9979.1 (7550.4 - 12744.6) | 12385.9 (9628.9 - 15494.4) |
| Germany | 6978.5 (5299.7 - 9073.2) | 11612.2 (9314.8 - 14331.3) | 13717.8 (11025.2 - 16893.8) | 6765.9 (5086.8 - 8900.4) | 10428.8 (8046.0 - 13354) | 12475.4 (9704.8 - 15764.4) | 6850.8 (5030.4 - 9133.5) | 10287.9 (7924.8 - 13179.3) | 11833.7 (8975.3 - 15073.7) |
| Greece | 6739.3 (5134.3 - 8822.6) | 11742.1 (9402.7 - 14450.4) | 14976.3 (12086.3 - 18205.1) | 6698.3 (5133.9 - 8727.6) | 10956.8 (8637.7 - 13621.4) | 13869.2 (11042.1 - 16933.3) | 6853.1 (5049.3 - 9070.9) | 10662.7 (8115.9 - 13388.2) | 13229.8 (10225.9 - 16367.9) |
| Hungary | 6545.0 (5168.4 - 8326) | 9908.0 (8124.6 - 12111.3) | 12187.4 (9986.0 - 14595.6) | 5990.2 (4659.5 - 7662.9) | 9280.9 (7506.3 – 11487.0) | 10936.2 (8719.5 - 13332.7) | 5679.8 (4262.3 - 7395.7) | 9192.4 (7338.4 - 11413.4) | 11777.3 (9418.3 - 14373.8) |
| Iceland | 6543.2 (4928.6 - 8605.5) | 11545.5 (9345.4 - 14172.1) | 14219.2 (11628.2 - 17200.7) | 6627.9 (5028.7 - 8654.9) | 10197.7 (7999.6 - 12851.3) | 12167.6 (9474.0 - 15195.7) | 6894.2 (5281.0 - 9019.6) | 9820.0 (7593.4 - 12387.1) | 11820.4 (9190.1 - 14864.8) |
| Ireland | 7069.6 (5409.6 - 9078.4) | 12436.0 (10182.3 - 15109.8) | 15507.6 (12875.5 - 18511.7) | 6924.6 (5193.8 - 9044.9) | 11445.1 (9059.4 - 14343) | 14067.1 (11224.5 - 17248.7) | 6667.1 (4854.3 – 8800.0) | 10383.9 (7948.8 - 13248.9) | 12732.5 (9720.5 - 16090.9) |
| Israel | 6454.9 (4999.2 - 8351.7) | 10900.2 (8833.1 - 13313.2) | 12738.7 (10180.1 - 15685.4) | 6157.9 (4679.6 - 8015.2) | 9510.3 (7408.0 – 11962.0) | 11555.1 (9056.8 - 14374.8) | 6101.4 (4505.8 - 8046.6) | 9977.6 (7786.4 - 12570.8) | 11782.7 (9220.2 - 14742.7) |
| Italy | 6756.5 (5166.4 - 8780.2) | 11153.4 (8879.3 - 13926.9) | 13789.2 (11081.4 - 17004.7) | 6356.8 (4782.3 - 8362.2) | 10057.9 (7787.4 - 12756.1) | 12120.7 (9491.2 - 15309.9) | 6590.6 (4883.2 - 8737.2) | 9874.6 (7503.0 - 12773.5) | 11470.9 (8736.9 - 14715.2) |
| Japan | 5768.3 (4443.7 - 7401.2) | 8930.5 (7199.3 - 10977.6) | 10810.6 (8730.4 - 13148.7) | 5520.7 (4197.2 – 7130.0) | 8260.8 (6541.3 - 10303.1) | 10550.8 (8469.6 - 12910.3) | 5573.2 (4253.9 – 7220.0) | 8248.2 (6467.1 - 10377.9) | 10181.3 (8039.4 - 12649.4) |
| Latvia | 6841.0 (5590.2 - 8502.2) | 12400.2 (10670.8 - 14500.2) | 18473.4 (16337.1 - 21016.8) | 6332.9 (5126.9 - 7852.7) | 9930.3 (8251.7 - 11894.8) | 13115.6 (11013.4 - 15412.9) | 5494.3 (4126.4 - 7134.5) | 9057.0 (7214.0 - 11320.3) | 13945.8 (11586.7 - 16624.7) |
| Lithuania | 6845.5 (5569.3 – 8519.0) | 12930.9 (11157.4 - 15006.7) | 18348.4 (16156.2 - 20815.9) | 6268.4 (5000.1 - 7802.8) | 11172.9 (9472.4 - 13148.2) | 15483.3 (13321.6 - 17875.6) | 6268.1 (4862.9 - 8040.1) | 9567.5 (7604.5 - 11817.6) | 13448.6 (10960.3 - 16181.5) |
| Luxembourg | 6431.3 (4840.1 - 8369.1) | 11139.6 (8944.0 - 13799.3) | 14563.6 (11814.8 - 17667.8) | 6065.4 (4519.3 - 8014.9) | 9749.6 (7598.9 - 12410.2) | 12489.4 (9825.0 - 15494.5) | 6278.3 (4665.0 - 8420.8) | 10043.7 (7774.6 - 12762.8) | 11688.1 (8844.5 - 14831.3) |
| Mexico | 8049.0 (6611.1 - 9915.5) | 11964.9 (10162.1 - 14270.9) | 15055.9 (12845.9 - 17567.6) | 7854.3 (6397.3 - 9679.9) | 13230.1 (11396.8 - 15549.2) | 16737.0 (14474.5 – 19342.0) | 7480.0 (6021.0 - 9299.1) | 13128.3 (11231.0 – 15500.0) | 18752.8 (16259.5 - 21497.7) |
| Netherlands | 6572.2 (5012.5 - 8466.6) | 10304.0 (8186.0 – 12687.0) | 12122.6 (9685.1 - 14816.6) | 6364.8 (4755.2 - 8304.3) | 9323.8 (7152.3 - 11867.4) | 10965.3 (8547.6 - 13861.1) | 6647.0 (4933.3 - 8728.3) | 9483.1 (7161.4 – 12269.0) | 11713.5 (9086.6 - 14883.1) |
| New Zealand | 7759.5 (6136.5 - 9786.9) | 13909.1 (11534.6 - 16639.5) | 16704.4 (13644.1 - 19955.4) | 6994.9 (5384.9 - 8791.8) | 12673.9 (10395.0 - 15284.8) | 15204.2 (12231.1 - 18326.8) | 6528.4 (4990.7 - 8394.3) | 11485.4 (9112.2 - 14238.2) | 14432.9 (11342.8 - 17720.6) |
| Norway | 6545.3 (4960.2 - 8464.5) | 11773.5 (9601.3 - 14266.6) | 14514.4 (11817.5 - 17472.4) | 6563.7 (4900.4 - 8642.4) | 10785.9 (8533.5 - 13544.4) | 13244.9 (10515.9 - 16271.0) | 6604.0 (4948.0 - 8721.4) | 10107.7 (7853.8 - 12878.8) | 11957.3 (9221.9 - 14938.6) |
| Poland | 6009.0 (4750.6 - 7631.1) | 10088.6 (8397.0 - 12160.4) | 12926.8 (10732.6 - 15380.3) | 5670.3 (4430.0 - 7273.7) | 9553.4 (7852.2 - 11644.1) | 11991.3 (9857.6 - 14409.0) | 5744.7 (4348.4 – 7486.0) | 9337.7 (7504.5 - 11509.6) | 12016.0 (9747.8 – 14560.0) |
| Portugal | 7808.1 (6076.1 - 9883.7) | 12969.1 (10533.1 - 15650.5) | 16173.2 (13393.5 – 19365.0) | 7049.9 (5343.4 - 9168.1) | 10730.9 (8357.3 - 13577.5) | 13259.9 (10463.8 – 16520.0) | 7277.1 (5404.5 - 9746.4) | 10699.7 (8133.8 - 13598.7) | 12552.1 (9597.5 - 15725.9) |
| Republic of Korea | 6396.0 (5071.9 - 8040.6) | 10059.7 (8279.3 - 12228.9) | 12075.9 (10022.7 - 14424.4) | 5828.6 (4509.8 – 7481.0) | 8750.6 (6996.7 - 10862.7) | 10551.6 (8505.5 - 12875.7) | 5384.1 (4077.1 - 6990.7) | 8236.1 (6432.6 - 10331.9) | 9973.6 (7804.0 - 12304.3) |
| Slovakia | 6682.3 (5300.2 - 8355.9) | 9938.2 (8134.4 - 12113.3) | 12527.6 (10310.2 - 14911.4) | 6133.3 (4798.0 - 7677.7) | 9164.7 (7384.5 – 11303.0) | 11535.1 (9384.7 - 13936.6) | 5907.3 (4564.9 - 7563.7) | 9379.0 (7500.2 - 11617.1) | 11492.6 (9186.3 - 14095.4) |
| Slovenia | 5932.3 (4616.2 - 7555.2) | 10773.1 (8937.8 - 12984.9) | 13279.5 (11058.2 - 15701.3) | 5499.8 (4175.5 - 7127.7) | 8918.5 (7128.4 - 11090.7) | 11448.5 (9189.6 - 13865.1) | 5415.0 (4051.5 - 7133.4) | 8908.3 (7104 - 11119.9) | 10148.5 (7914.9 - 12631.4) |
| Spain | 6612.9 (5113.9 - 8520.2) | 10829.1 (8707.2 – 13480.0) | 12807.5 (10289.3 - 15789.4) | 6437.6 (4810.1 - 8402.9) | 9519.7 (7390.6 - 12192.6) | 11099.1 (8572.5 - 14162.6) | 6695.0 (5003.6 - 8864.7) | 9711.5 (7441.9 - 12581.5) | 11663.6 (8810.8 - 14786.7) |
| Sweden | 6294.4 (4782.2 - 8149.6) | 9956.3 (7851.3 - 12575.5) | 12353.8 (9808.1 - 15383.4) | 6074.8 (4591.2 - 7953.1) | 9688.4 (7603.8 - 12362.5) | 12128.6 (9578.2 - 15097.6) | 6232.2 (4636.3 - 8132.1) | 9498.6 (7313.1 - 12135.8) | 12445.0 (9782.0 - 15513.1) |
| Switzerland | 7197.0 (5590.5 - 9232.9) | 11690.0 (9298.6 – 14487.0) | 15052.4 (12073.8 - 18347.1) | 6608.4 (4936.6 - 8668.7) | 10500.3 (8187.6 - 13248.2) | 12259.2 (9511.0 - 15343.9) | 6597.2 (4898.6 - 8735.9) | 9980.7 (7685.4 - 12638.8) | 11667.4 (8919.7 - 14711.9) |
| Türkiye | 8998.9 (7417.3 - 11025.7) | 12788.3 (10719.9 - 15344.7) | 13881.8 (11672.3 – 16578.0) | 7906.9 (6307.6 - 9843.1) | 11249.5 (9295.4 - 13536.2) | 12182.7 (9966.5 - 14676.4) | 7621.9 (5950.9 - 9719.5) | 11322.1 (9031.7 - 13796.4) | 12817.7 (10232.9 – 15520.0) |
| United Kingdom | 6732.2 (5134.9 - 8653.4) | 11261.0 (8973.1 – 13970.0) | 14126.9 (11278.6 - 17286.3) | 6307.5 (4726.1 - 8247.5) | 10374.0 (8103.7 - 13067.3) | 12961.0 (10144.0 - 16100.2) | 6564.1 (4902.1 - 8611.6) | 10437.2 (8082.4 - 13227.7) | 13199.2 (10250.6 - 16413.2) |
| United States of America | 6805.2 (5379.0 - 8544.6) | 13620.3 (11229.6 - 16311.3) | 17018.3 (13981.7 - 20221.5) | 6521.0 (5022.6 - 8337.5) | 12517.6 (10093.7 - 15240.2) | 16748.0 (13722.5 - 19981.4) | 7164.0 (5538.7 - 9108.4) | 14117.2 (11335.0 - 17154.2) | 20421.5 (16860.1 - 24212.9) |
| *UI: uncertainty intervals | | | | | | | | | |

#### Table S7: Number of DALYs among adolescents and young adults aged 10-24 years in 2000, 2010, and 2021, stratified by gender

| Country | Number of DALYs (95% UI) | | | | | |  |
| --- | --- | --- | --- | --- | --- | --- | --- |
|  | Females | | | Males | | |  |
|  | 2000 | 2010 | 2021 | 2000 | 2010 | 2021 |  |
| *All OECD countries average* | *13757429.9 (10775836.0 - 17361782.7)* | *13270556.9 (16889679.2 - 10250789.8)* | *13806607.9 (10614666.5 - 17640841.9)* | *16355433.6 (14023450.3 - 19062146.1)* | *14915186.5 (17593534.7 - 12582714.7)* | *14957683.8 (12600834.5 - 17836631.3)* |  |
| Australia | 223493.9 (173627.1 - 280224.7) | 235170.5 (297880.9 - 179296.8) | 247223.3 (186908.7 - 314429.3) | 245305.3 (207146.8 – 290496.0) | 231837.2 (279936.2 – 189419.0) | 242440.3 (197934.6 - 292875.3) |  |
| Austria | 71694.5 (55339.9 - 91510.4) | 71980.9 (93748.0 - 54408.4) | 68885.9 (50447.4 - 91034.3) | 83199.3 (70403.0 - 97987.6) | 77039.2 (92373.5 - 63393.2) | 68545.3 (54513.7 - 84299.8) |  |
| Belgium | 97545.0 (74645.5 - 126289.7) | 98266.6 (129391.1 – 74195.0) | 98684.5 (72627.6 - 129105.7) | 112161.6 (95292.3 - 133026.6) | 97271.1 (119741.8 - 78884.8) | 89470.4 (71086.4 - 112230.1) |  |
| Canada | 309735.6 (238037.4 - 395665.9) | 318790.7 (408856.5 - 243170.6) | 331904.1 (251376.5 – 424956.0) | 330882.0 (278864.3 - 391656.7) | 328361.1 (391520.8 - 270586.3) | 335060.4 (274504.5 - 400982.6) |  |
| Chile | 203835.8 (158773.2 - 257015.5) | 222218.0 (278686.4 - 172106.7) | 214760.9 (162266.6 - 272805.5) | 223828.1 (192972.8 - 261403.8) | 238631.8 (278824.9 - 203419.5) | 209972.0 (173617.7 – 248863.0) |  |
| Colombia | 725535.7 (600603.7 - 877414.6) | 662968.6 (828844.9 - 532225.1) | 634725.9 (505785.4 - 793203.3) | 1451986.6 (1352592.2 - 1566858.6) | 1084053.3 (1212045.6 - 981950.3) | 943097.0 (821362.6 - 1073770.7) |  |
| Costa Rica | 57389.2 (45386.9 - 72970.1) | 61702.4 (77797.0 - 48482.6) | 56870.4 (44424.9 - 72420.5) | 67260.8 (57620.1 - 78578.8) | 74377.7 (86599.9 - 63991.4) | 70622.8 (61484.2 - 82117.9) |  |
| Czechia | 98101.0 (76088.9 - 124209.5) | 73839.6 (93796.6 - 56242.8) | 64586.7 (48823.9 - 83324.1) | 122294.4 (103936.3 - 143419.5) | 87450.8 (104879.8 - 72881.2) | 67476.5 (55006.9 - 83037.3) |  |
| Denmark | 45938.6 (34906.9 - 59314.6) | 48479.8 (63061.8 - 36384.4) | 50913.6 (38101.3 – 66896.0) | 49129.6 (40928.6 – 58912.0) | 45718.7 (56236.9 – 36394.0) | 45997.2 (36596.5 - 57349.8) |  |
| Estonia | 14613.4 (11619.8 - 18154.7) | 10637.4 (13429.4 - 8267.9) | 9389.3 (7428.2 - 11746.1) | 23536.2 (20962.7 - 26446.1) | 14603.2 (16946.9 – 12499.0) | 9790.9 (8105.2 - 11733.3) |  |
| Finland | 50212.2 (38634.8 - 65041.8) | 48437.2 (62755.9 - 36631.4) | 45903.4 (34516.2 - 59554.5) | 57788.4 (48694.0 - 69142.4) | 53326.4 (63929.2 - 44203.3) | 48236.3 (39425.6 - 58605.6) |  |
| France | 636579.1 (487952.6 - 817769.1) | 599751.2 (792671.4 - 447791.1) | 635154.6 (464967.2 - 826306.2) | 658040.0 (550918.5 - 784511.9) | 575388.7 (698490.7 - 468685.4) | 548043.1 (433516.5 - 686766.8) |  |
| Germany | 742358.7 (565694.7 - 965215.9) | 687730.7 (905213.7 - 515931.7) | 649929.9 (477010.7 - 860043.7) | 764919.9 (634060.8 - 916086.8) | 636769.9 (787557.5 - 505921.3) | 589010.2 (463500.2 - 740190.2) |  |
| Greece | 117610.2 (87698.3 - 151903.1) | 92556.1 (119884.3 - 69959.2) | 81575.9 (59801.9 - 105722.9) | 140067.3 (117764.0 - 166218.1) | 99298.6 (119351.7 - 81914.4) | 76149.9 (61202.2 - 93412.8) |  |
| Hungary | 97661.2 (76084.8 - 123676.5) | 73868.3 (94948.5 - 56435.6) | 63501.3 (48100.5 - 81196.3) | 113675.0 (96669.9 - 134075.1) | 80568.9 (96467.1 - 66861.4) | 65964.6 (53699.0 - 80026.3) |  |
| Iceland | 3336.1 (2567.0 - 4326.3) | 3453.1 (4479.3 - 2599.3) | 3339.3 (2498.8 - 4364.8) | 3542.9 (2944.3 - 4229.5) | 3224.8 (3956.2 - 2581.6) | 3126.6 (2523.7 - 3880.3) |  |
| Ireland | 51904.6 (40302.6 - 66769.7) | 48148.8 (62264.5 - 36040.8) | 50698.0 (37459.8 – 66367.0) | 60538.8 (51343.9 - 71174.6) | 49450.5 (59355.3 - 40546.8) | 45107.8 (35328.2 - 56565.6) |  |
| Israel | 78040.0 (58905.1 – 101063.0) | 83715.0 (110662.8 – 62290.0) | 102666.0 (76148.8 - 135374.9) | 89577.8 (75418.4 - 106200.5) | 84855.3 (103040.8 - 68885.8) | 103013.4 (83509.4 - 126271.7) |  |
| Italy | 496368.3 (375227.8 - 647416.8) | 439529.1 (577535.3 - 326632.3) | 435506.5 (323041.3 - 577220.1) | 522081.5 (435238.9 - 625492.4) | 414147.2 (509377.1 - 333489.7) | 377124.5 (293002.6 – 475882.0) |  |
| Japan | 968448.9 (745553.7 - 1233448.2) | 775446.8 (987103.7 - 595802.3) | 729944.7 (559888.0 - 930475.5) | 1036441.6 (869018.9 - 1247784.5) | 777690.4 (946583.9 - 643759.4) | 688961.4 (560108.2 - 849954.8) |  |
| Latvia | 25455.4 (20567.1 – 31509.0) | 17540.1 (22000.8 - 13780.9) | 11838.5 (9073.4 - 15185.3) | 39230.3 (35142.6 - 43918.7) | 23316.4 (26833.3 - 20338.4) | 13131.9 (10983.5 - 15832.4) |  |
| Lithuania | 37382.2 (29711.5 - 46716.9) | 27620.4 (34649.5 - 21545) | 18845.7 (14392.4 - 24058.3) | 59359.7 (53426.7 - 66637.3) | 43123.2 (48514.8 - 38234.3) | 22039.9 (18453.9 - 26019.3) |  |
| Luxembourg | 3925.2 (3002.4 - 5008.9) | 4294.5 (5642.3 - 3215.4) | 5341.8 (3953.3 – 7038.0) | 4196.7 (3482.9 - 5019.3) | 4185.1 (5144.7 - 3358.9) | 4759.9 (3748.4 - 5964.7) |  |
| Mexico | 1659220.7 (1332923.9 - 2067853.9) | 1766669.3 (2190581.7 - 1425353.4) | 1864339.2 (1494621.1 – 2322748.0) | 1969034.7 (1732113.3 - 2246014.6) | 2314944.7 (2627238.8 - 2061877.7) | 2463821.7 (2208969.1 - 2788468.1) |  |
| Netherlands | 139896.0 (105811.2 - 179043.5) | 140626.7 (183355.5 - 105022.5) | 151448.2 (111830.3 - 198403.4) | 135223.6 (111293.6 - 161726.2) | 128197.3 (159237.5 - 102050.4) | 131147.4 (101985.0 - 164677.9) |  |
| New Zealand | 49347.8 (38555.1 - 61152.5) | 53845.5 (67131.1 - 41671.2) | 56858.7 (43915.6 - 71924.3) | 54671.3 (46684.5 - 64198.5) | 55608.0 (65766.6 - 46712.8) | 53150.4 (43195.1 - 64845.2) |  |
| Norway | 42328.9 (32504.4 - 54020.3) | 47922.2 (62666.4 - 36427.5) | 49893.1 (37691.4 - 65136.5) | 47804.4 (39998.7 - 56405.6) | 48122.4 (58696.3 – 38873.0) | 44695.5 (35387.0 - 55442.4) |  |
| Poland | 396640.4 (309097.7 – 504770.0) | 301639.9 (388066 - 231197.9) | 244758.4 (187895.6 - 313473.1) | 522866.1 (450887.5 - 612413.2) | 394652.0 (463750.9 - 337740.8) | 276787.4 (228598.1 - 333745.9) |  |
| Portugal | 127800.0 (98265.4 - 161959.6) | 96214.7 (125189.8 - 72339.9) | 89842.7 (65668.0 - 117051.1) | 150295.1 (128929.4 - 174512.6) | 87996.8 (107469.0 - 71130.9) | 79126.7 (62711.8 - 98254.1) |  |
| Republic of Korea | 487203.9 (385676.3 - 615737.4) | 400459.8 (509791.3 - 310858.5) | 316665.5 (242810.6 - 406654.9) | 566921.9 (482945.5 - 670336.1) | 436666.1 (528188.8 - 357939.8) | 305545.9 (243531.4 - 376648.5) |  |
| Slovakia | 58428.7 (45387.9 - 73671.2) | 44821.4 (57179.1 - 34222.5) | 36148.6 (27642.9 - 46011.8) | 72489.5 (61683.6 – 85570.0) | 53136.8 (63070.3 - 44695.8) | 38106.0 (31442.4 - 46252.3) |  |
| Slovenia | 18280.7 (14190.9 - 23074.2) | 13342.1 (17205.6 - 10132.9) | 11704.9 (8806.9 - 15196.1) | 24416.4 (21054.3 - 28546.2) | 16160.3 (19463.0 - 13432.3) | 12080.9 (9754.7 - 14782.6) |  |
| Spain | 406756.6 (307843.3 - 530030.9) | 344121.0 (452691.9 - 254778.7) | 353348.5 (262654.0 - 462883.7) | 457250.6 (384397.1 - 543851.7) | 311401.3 (384682.5 - 249317.2) | 295361.2 (232736.9 - 369847.7) |  |
| Sweden | 76192.3 (57686.4 – 99380.0) | 84988.0 (110404.1 - 63746.4) | 85399.2 (62973.8 - 112640.1) | 75360.5 (61671.9 - 91547.3) | 80947.6 (99120.4 - 65704.1) | 79111.0 (63491.7 - 97017.3) |  |
| Switzerland | 69631.1 (52965.9 - 89127.2) | 69812.6 (90651.0 - 52383.7) | 67543.9 (49959.6 - 88387.6) | 75482.8 (62493.5 - 90053.2) | 66759.1 (82296.8 – 53278.0) | 61548.1 (48159.0 - 76435.3) |  |
| Türkiye | 1097130.7 (877515.3 - 1366498.9) | 931124.4 (1172408.3 - 731399.9) | 1001073.3 (767582.6 - 1276119.7) | 1326497.0 (1136608.2 – 1538449.0) | 1079105.2 (1265407.3 - 904998.7) | 1038372.3 (853094.3 - 1236850.4) |  |
| United Kingdom | 573174.0 (438507.4 - 737559.4) | 615217.7 (797611.0 - 465541.1) | 634834.7 (474908.5 - 823628.4) | 596901.5 (490813.3 - 715708.6) | 587237.9 (718852.6 - 470955.7) | 582075.6 (461954.0 - 718176.4) |  |
| United States of America | 3398233.3 (2649227.9 - 4265352.3) | 3653605.9 (4611901.3 - 2804271.6) | 4230558.5 (3257160.4 - 5320693.4) | 4021174.2 (3435165.3 - 4673666.4) | 4129561.3 (4867918.1 - 3475833.9) | 4829611.2 (4112840.1 - 5652713.5) |  |
| *UI: uncertainty intervals | | | | | | | |

#### Table S8: Rate of DALYs among adolescents and young adults aged 10-24 years in 2000, 2010, and 2021, stratified by gender

| Country | DALYs rate per 100,000 population  Rate/100k (95% UI) | | | | | |
| --- | --- | --- | --- | --- | --- | --- |
|  | Females | | | Males | | |
|  | 2000 | 2010 | 2021 | 2000 | 2010 | 2021 |
| *All OECD countries average* | *10624.8 (8322.2 - 13408.5)* | *10386.9 (8023.3 - 13219.6)* | *11118.1 (8547.8 - 14205.8)* | *12163.5 (10429.2 - 14176.4)* | *11214.2 (9460.5 - 13228.0)* | *11499.0 (9687.1 - 13712.3)* |
| Australia | 11540.7 (8965.7 - 14470.1) | 11149.7 (8500.6 - 14122.8) | 10748.3 (8126.1 - 13670.1) | 12147.1 (10257.5 - 14384.8) | 10503.4 (8581.7 - 12682.6) | 9999.3 (8163.7 - 12079.4) |
| Austria | 10324.3 (7969.1 - 13177.8) | 10095.7 (7631.1 - 13148.7) | 10161.5 (7441.6 - 13428.6) | 11467.0 (9703.3 - 13505.2) | 10371.2 (8534.2 - 12435.5) | 9327.0 (7417.7 - 11470.7) |
| Belgium | 10720.5 (8203.8 - 13879.6) | 10375.2 (7833.7 - 13661.4) | 10257.5 (7549.1 - 13419.6) | 11873.2 (10087.4 - 14081.9) | 9969.9 (8085.4 - 12273.1) | 8926.1 (7092.0 - 11196.7) |
| Canada | 10268.3 (7891.4 - 13117.1) | 10120.5 (7719.8 - 12979.8) | 10595.2 (8024.6 - 13565.7) | 10577.6 (8914.7 - 12520.4) | 10002.8 (8242.8 - 11926.9) | 10054.1 (8237.0 - 12032.3) |
| Chile | 10643.1 (8290.2 - 13419.8) | 10830.4 (8388.1 - 13582.5) | 11239.7 (8492.4 - 14277.5) | 11321.5 (9760.8 - 13222.1) | 11276.1 (9612.2 - 13175.3) | 10427.7 (8622.2 - 12359.1) |
| Colombia | 12441.7 (10299.4 - 15046.2) | 10690 (8581.8 - 13364.6) | 10876.7 (8667.1 - 13592.3) | 25195.4 (23470.6 - 27188.7) | 17091.0 (15481.3 - 19108.9) | 15378.2 (13393.2 - 17508.9) |
| Costa Rica | 9726.7 (7692.5 - 12367.5) | 9871.3 (7756.4 - 12446.2) | 10404.6 (8127.7 - 13249.5) | 11097.5 (9506.9 - 12964.9) | 11877.9 (10219.3 - 13829.8) | 12910.6 (11239.9 – 15012.0) |
| Czechia | 9160.8 (7105.3 - 11598.8) | 8865.0 (6752.4 - 11261.0) | 8487.5 (6416.1 - 10949.8) | 10899.3 (9263.2 - 12782.1) | 9976.5 (8314.4 - 11964.8) | 8426.2 (6869.1 - 10369.4) |
| Denmark | 10187.7 (7741.2 - 13154.1) | 9713.4 (7289.9 - 12635.0) | 9945.4 (7442.7 - 13067.4) | 10479.1 (8729.9 - 12565.6) | 8742.6 (6959.5 - 10754.0) | 8546.6 (6799.9 – 10656.0) |
| Estonia | 9887.6 (7862.1 - 12283.6) | 9098.0 (7071.5 - 11486.0) | 9533.8 (7542.5 - 11926.9) | 15017.9 (13375.7 - 16874.6) | 11768.7 (10072.9 - 13657.5) | 9437.5 (7812.7 - 11309.8) |
| Finland | 10504.7 (8082.7 - 13607.2) | 10342.9 (7822.0 - 13400.4) | 10270.5 (7722.7 - 13324.9) | 11567.7 (9747.3 - 13840.5) | 10910.4 (9043.8 - 13079.6) | 10261.5 (8387.2 - 12467.4) |
| France | 10992.5 (8426.0 - 14121.3) | 10352.2 (7729.2 - 13682.1) | 10625.0 (7778.1 - 13822.7) | 10963.4 (9178.6 - 13070.5) | 9574.3 (7798.8 - 11622.7) | 8780.5 (6945.6 – 11003.0) |
| Germany | 10833.9 (8255.7 - 14086.2) | 10735.8 (8053.9 - 14130.8) | 10874.6 (7981.3 - 14390.2) | 10646.3 (8824.9 - 12750.2) | 9483.1 (7534.5 - 11728.8) | 8860.2 (6972.2 - 11134.3) |
| Greece | 10997.2 (8200.3 - 14203.7) | 10759.2 (8132.5 - 13936) | 10765.9 (7892.3 - 13952.6) | 12027.1 (10112.0 - 14272.6) | 10753.7 (8871.0 - 12925.3) | 9687.2 (7785.7 - 11883.2) |
| Hungary | 9281.7 (7231.1 - 11754.2) | 8745.9 (6681.9 - 11241.8) | 8997.2 (6815.2 - 11504.4) | 10327.6 (8782.7 – 12181.0) | 9075.0 (7531.0 - 10865.7) | 8834.2 (7191.5 - 10717.4) |
| Iceland | 10656.7 (8199.8 - 13819.7) | 10258.8 (7722.2 - 13307.6) | 10019.7 (7497.6 - 13096.5) | 10916.3 (9071.8 – 13032.0) | 9208.9 (7372.2 - 11297.5) | 8970.2 (7240.5 - 11132.7) |
| Ireland | 11081.8 (8604.7 - 14255.6) | 10800.7 (8084.6 - 13967.1) | 10512.5 (7767.5 - 13761.6) | 12417.2 (10531.2 - 14598.7) | 10856.6 (8901.9 - 13031.2) | 9003.7 (7051.6 - 11290.7) |
| Israel | 9509.9 (7178.1 - 12315.4) | 9100.7 (6771.6 - 12030.2) | 9330.0 (6920.2 - 12302.5) | 10396.3 (8753.0 - 12325.5) | 8829.9 (7168.1 - 10722.2) | 8932.1 (7241.0 - 10948.8) |
| Italy | 10803.4 (8166.8 - 14090.9) | 10170.0 (7557.7 - 13363.2) | 10386.1 (7704.0 - 13765.7) | 10893.9 (9081.8 - 13051.7) | 9089.7 (7319.5 - 11179.9) | 8370.5 (6503.3 - 10562.4) |
| Japan | 8629.2 (6643.1 - 10990.4) | 8370.7 (6431.5 - 10655.5) | 8530.1 (6542.8 - 10873.5) | 8791.3 (7371.2 – 10584.0) | 7992.2 (6615.8 - 9727.8) | 7647.0 (6216.8 - 9433.9) |
| Latvia | 9867.7 (7972.8 - 12214.4) | 9158.7 (7195.8 - 11487.9) | 8970.5 (6875.3 - 11506.5) | 14621.4 (13097.9 - 16368.8) | 11626.0 (10141.1 - 13379.6) | 9426.7 (7884.5 - 11365.3) |
| Lithuania | 9769.4 (7764.8 - 12208.9) | 9071.2 (7075.9 - 11379.7) | 9378.2 (7162.1 - 11972.2) | 14997.6 (13498.6 - 16836.4) | 13468.5 (11941.5 - 15152.4) | 10409.9 (8716.2 - 12289.5) |
| Luxembourg | 10524.7 (8050.3 - 13430.6) | 9737.7 (7290.8 - 12793.8) | 10337.5 (7650.4 – 13620.0) | 10874.7 (9025.1 - 13006.3) | 9082.4 (7289.5 - 11165.1) | 8604.4 (6776.1 - 10782.4) |
| Mexico | 10388.2 (8345.3 - 12946.6) | 10750.5 (8673.5 - 13330.1) | 11298.5 (9057.9 - 14076.6) | 12671.3 (11146.6 - 14453.7) | 14182.7 (12632.3 - 16096) | 14770.2 (13242.4 - 16716.4) |
| Netherlands | 10010.1 (7571.2 - 12811.2) | 9498.8 (7093.8 - 12384.9) | 10293.6 (7600.9 - 13485.1) | 9315.8 (7667.2 - 11141.6) | 8339.2 (6638.3 - 10358.3) | 8524.8 (6629.2 - 10704.3) |
| New Zealand | 12143.6 (9487.7 - 15048.5) | 11693.6 (9049.7 - 14578.8) | 11647.7 (8996.3 - 14733.9) | 13023.1 (11120.6 - 15292.6) | 11707.5 (9834.8 - 13846.3) | 10137.2 (8238.4 - 12367.6) |
| Norway | 10468.9 (8039.1 - 13360.5) | 10416.7 (7918.1 - 13621.6) | 10419.8 (7871.5 - 13603.2) | 11324.4 (9475.3 - 13361.9) | 9945.2 (8033.7 - 12130.4) | 8786.9 (6956.9 - 10899.7) |
| Poland | 8596.2 (6698.9 - 10939.7) | 8367.9 (6413.8 - 10765.5) | 8624.1 (6620.5 - 11045.2) | 10884.6 (9386.2 - 12748.8) | 10494.3 (8980.9 - 12331.7) | 9282.7 (7666.5 - 11192.9) |
| Portugal | 11957.7 (9194.3 - 15153.9) | 11053.2 (8310.5 - 14381.9) | 11180.1 (8171.8 – 14566.0) | 13547.1 (11621.2 - 15729.9) | 9774.6 (7901.2 - 11937.6) | 9455.7 (7494.1 - 11741.5) |
| Republic of Korea | 9447.8 (7479.0 - 11940.3) | 8487.3 (6588.3 - 10804.5) | 8499.1 (6516.9 - 10914.4) | 9987.9 (8508.5 - 11809.9) | 8285.6 (6791.8 - 10022.2) | 7673.0 (6115.7 - 9458.6) |
| Slovakia | 8981.9 (6977.2 – 11325.0) | 8664.6 (6615.7 - 11053.5) | 8881.1 (6791.4 - 11304.4) | 10692.7 (9098.8 - 12622.2) | 9824.9 (8264.2 - 11661.6) | 8883.1 (7329.7 - 10782.1) |
| Slovenia | 9000.4 (6986.8 - 11360.4) | 8356.7 (6346.7 - 10776.6) | 8164.8 (6143.3 - 10600.1) | 11391.4 (9822.8 - 13318.2) | 9492.3 (7889.9 - 11432.2) | 7923.2 (6397.6 - 9695.2) |
| Spain | 10144.1 (7677.3 - 13218.4) | 9883.8 (7317.7 - 13002.2) | 10366.4 (7705.6 - 13579.9) | 10847.2 (9118.9 - 12901.6) | 8518.4 (6820.1 - 10523.0) | 8232.1 (6486.7 - 10308.2) |
| Sweden | 9681.4 (7329.9 - 12627.7) | 10050.6 (7538.6 - 13056.3) | 9994.7 (7370.1 - 13182.8) | 9116.2 (7460.3 - 11074.3) | 9090.8 (7378.9 - 11131.7) | 8599.9 (6901.9 - 10546.4) |
| Switzerland | 11098.1 (8442.0 - 14205.5) | 10400.6 (7804.0 - 13505.1) | 10296.6 (7616.0 - 13474.1) | 11519.8 (9537.4 - 13743.4) | 9527.3 (7603.4 - 11744.7) | 8746.9 (6844.1 - 10862.6) |
| Türkiye | 11106.3 (8883.2 - 13833.2) | 9888.3 (7767.3 - 12450.6) | 10670.1 (8181.4 - 13601.7) | 12610.2 (10805.0 - 14625.1) | 10906.2 (9146.6 - 12789.2) | 10520.8 (8643.6 - 12531.8) |
| United Kingdom | 10542.2 (8065.4 - 13565.7) | 10460.9 (7915.8 - 13562.2) | 10668.4 (7980.9 - 13841.1) | 10638.8 (8748.0 - 12756.4) | 9617.1 (7712.8 - 11772.5) | 9469.9 (7515.6 - 11684.1) |
| United States of America | 11618.7 (9057.8 - 14583.4) | 11555.8 (8869.5 - 14586.7) | 13337.5 (10268.7 - 16774.4) | 13058.8 (11155.7 - 15177.8) | 12458.2 (10486.0 - 14685.6) | 14598.7 (12432.1 - 17086.7) |
| *UI: uncertainty intervals | | | | | | |

#### Figure S1: Top 15 risk factors of death for adolescent and young adults (10-24 years, both genders) in Australia in the years 2000, 2010, and 2021

1. 10-14 years


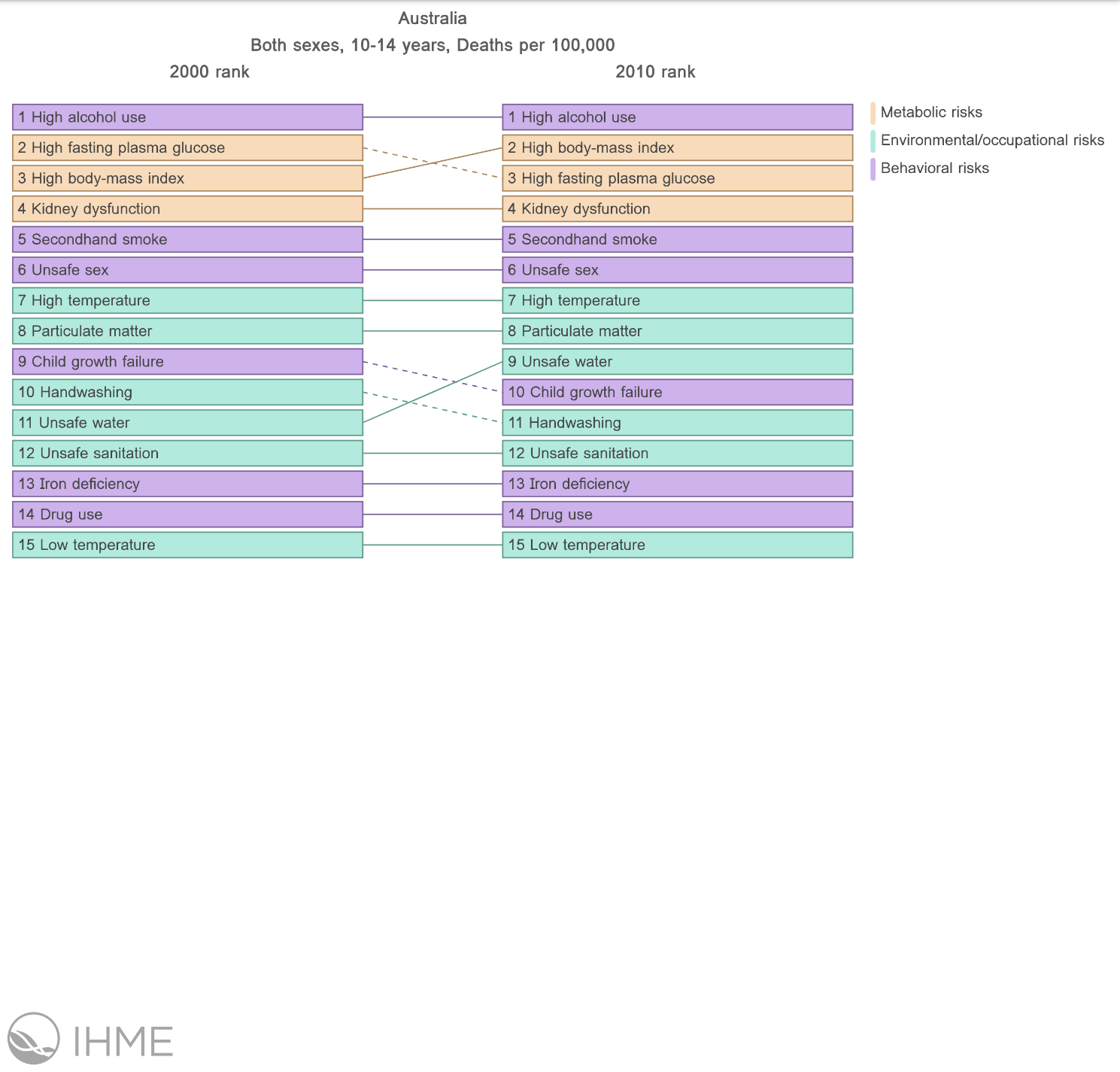

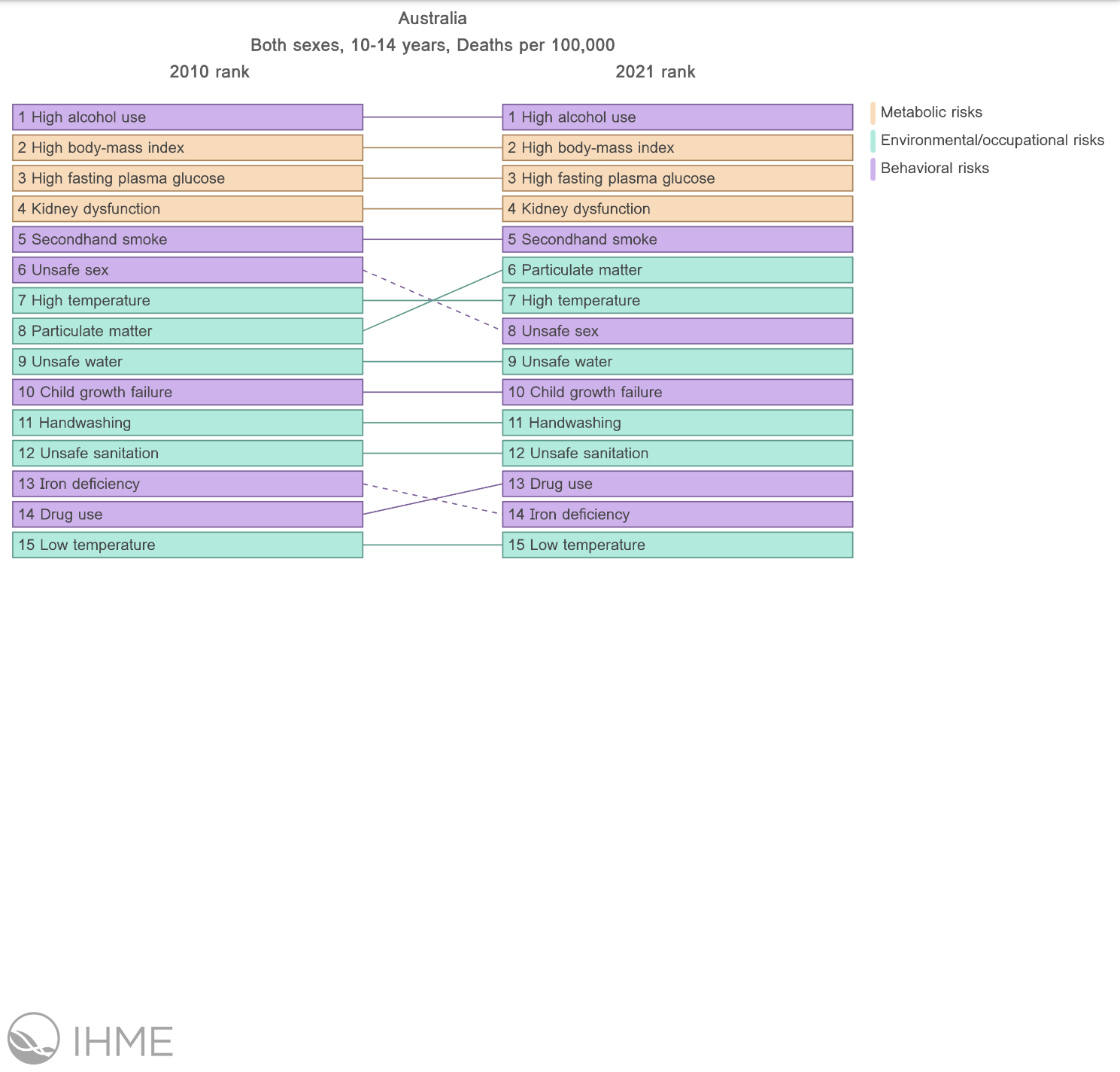


1. 15-19 years


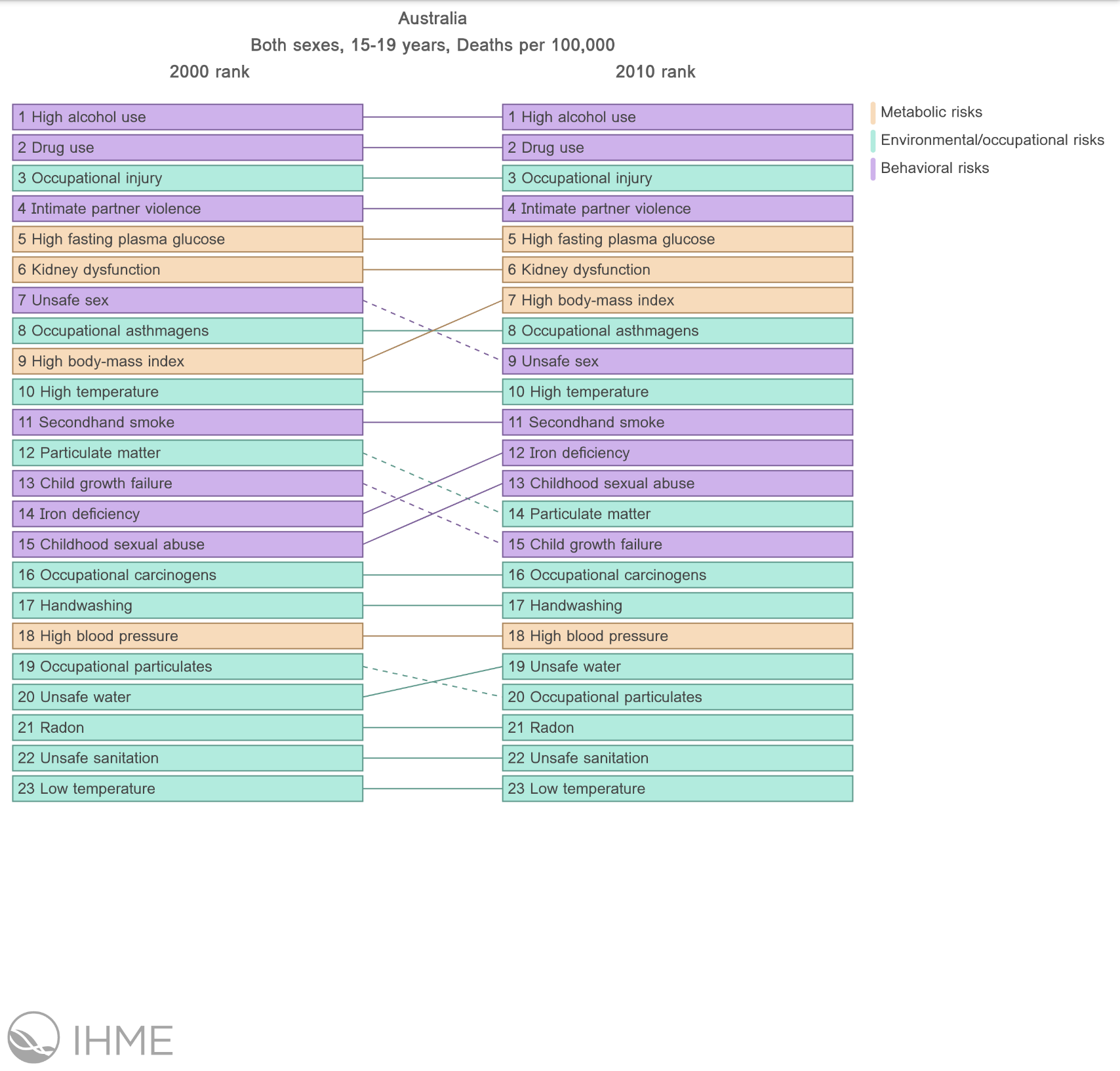

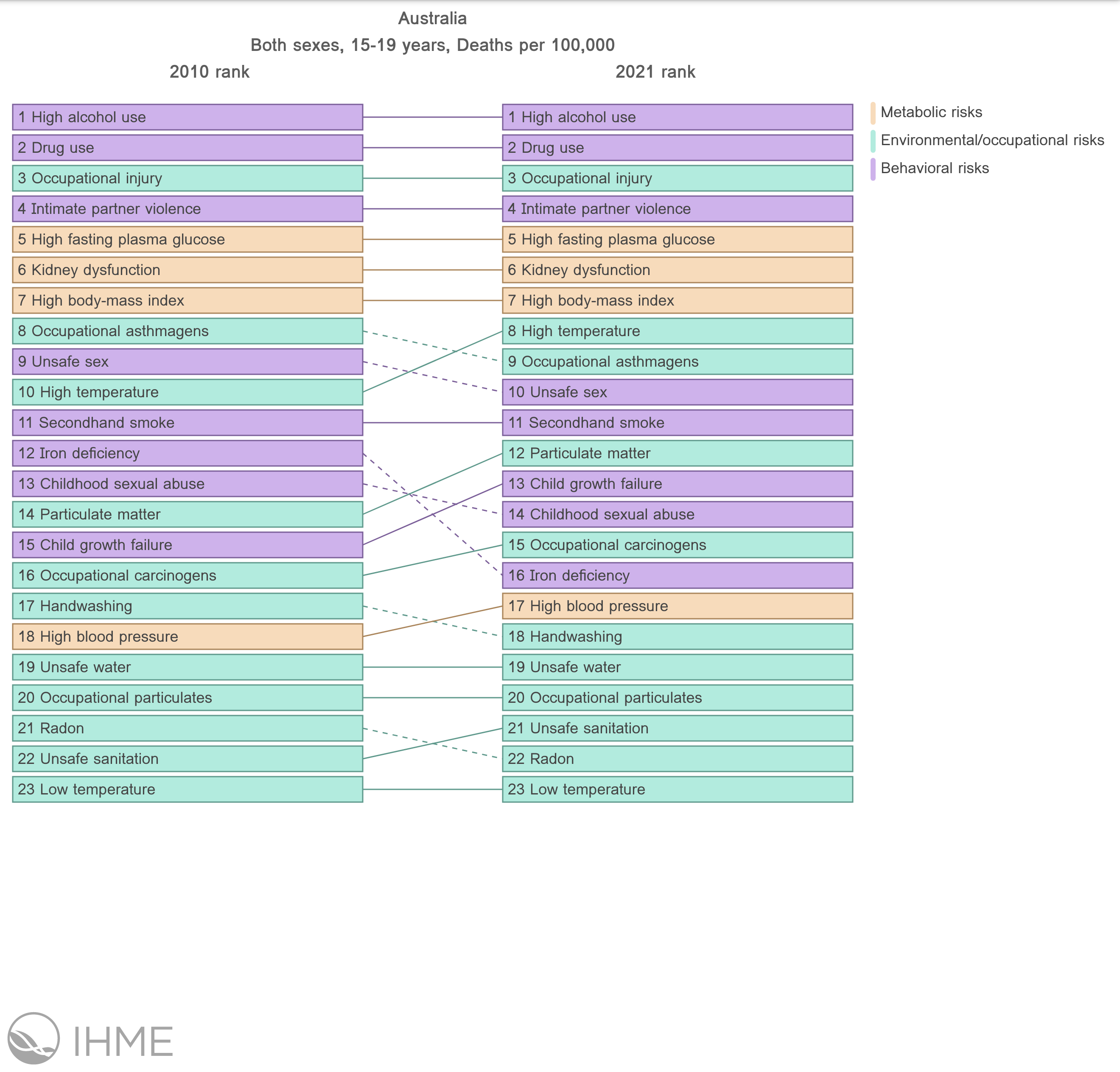


1. 20-24 years


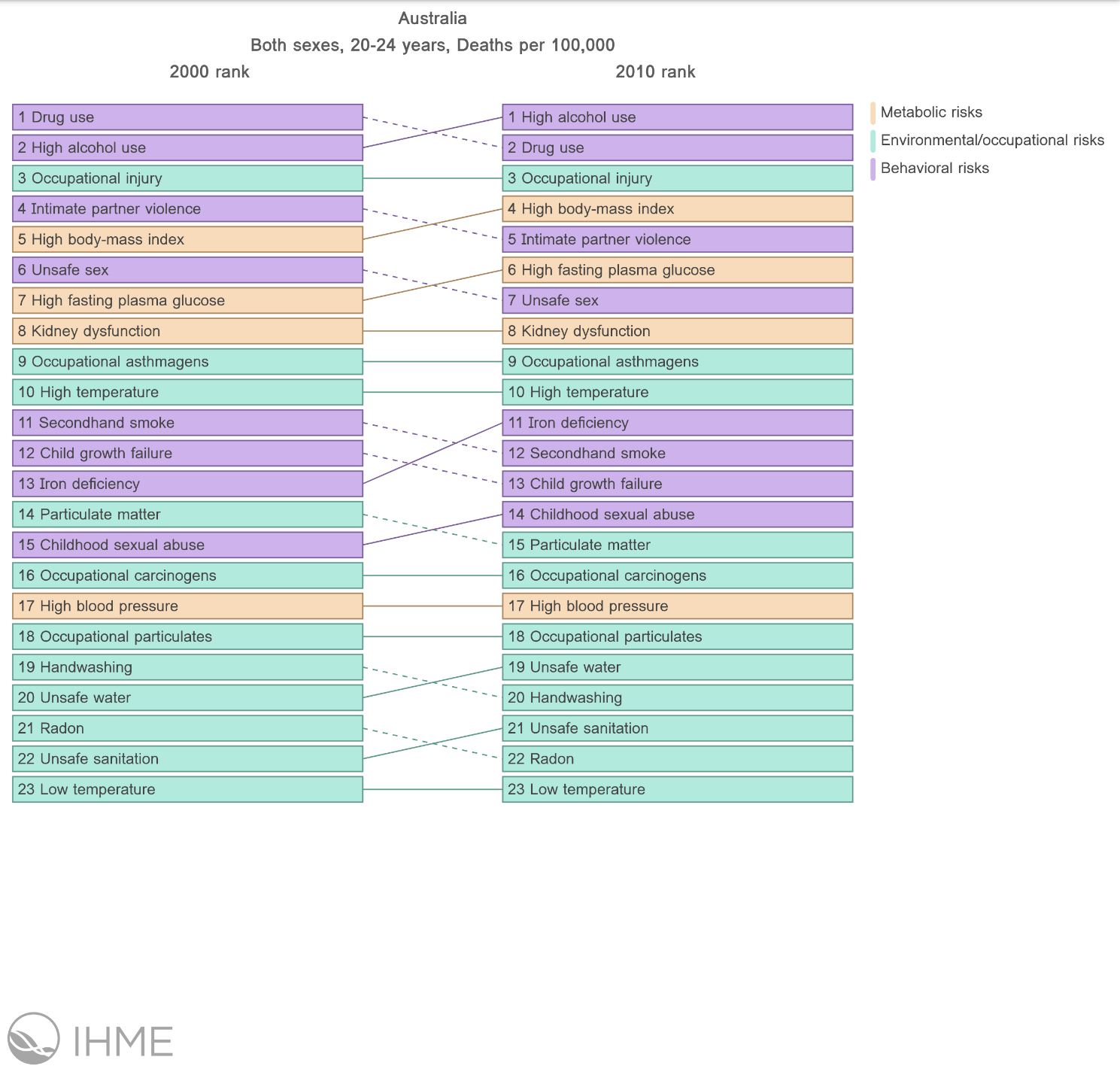

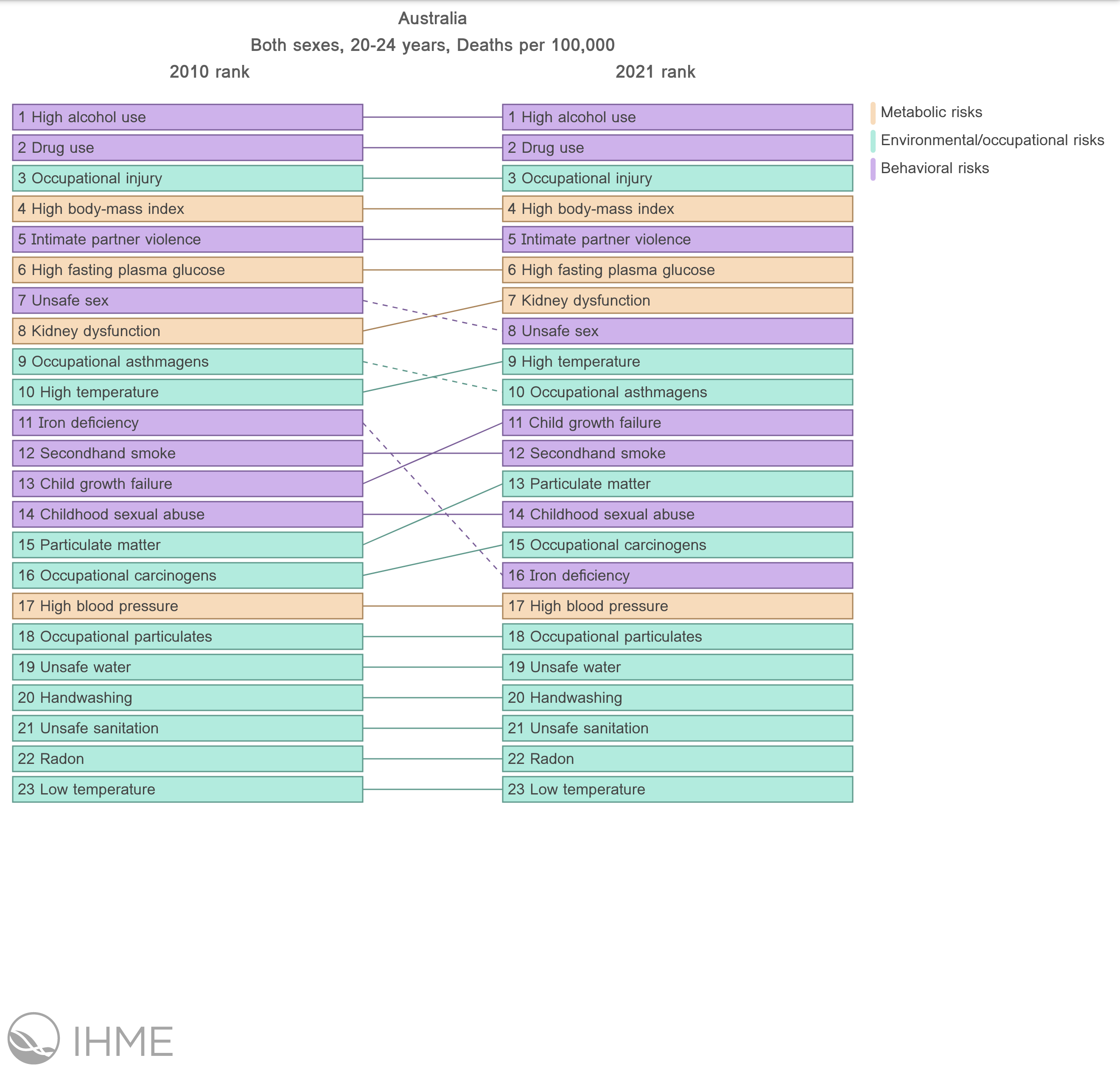


#### Figure S2: Top 15 risk factors of death for adolescents and young adults (10-24 years) in OECD Countries in the years 2000, 2010, and 2021

1. 10-24 years


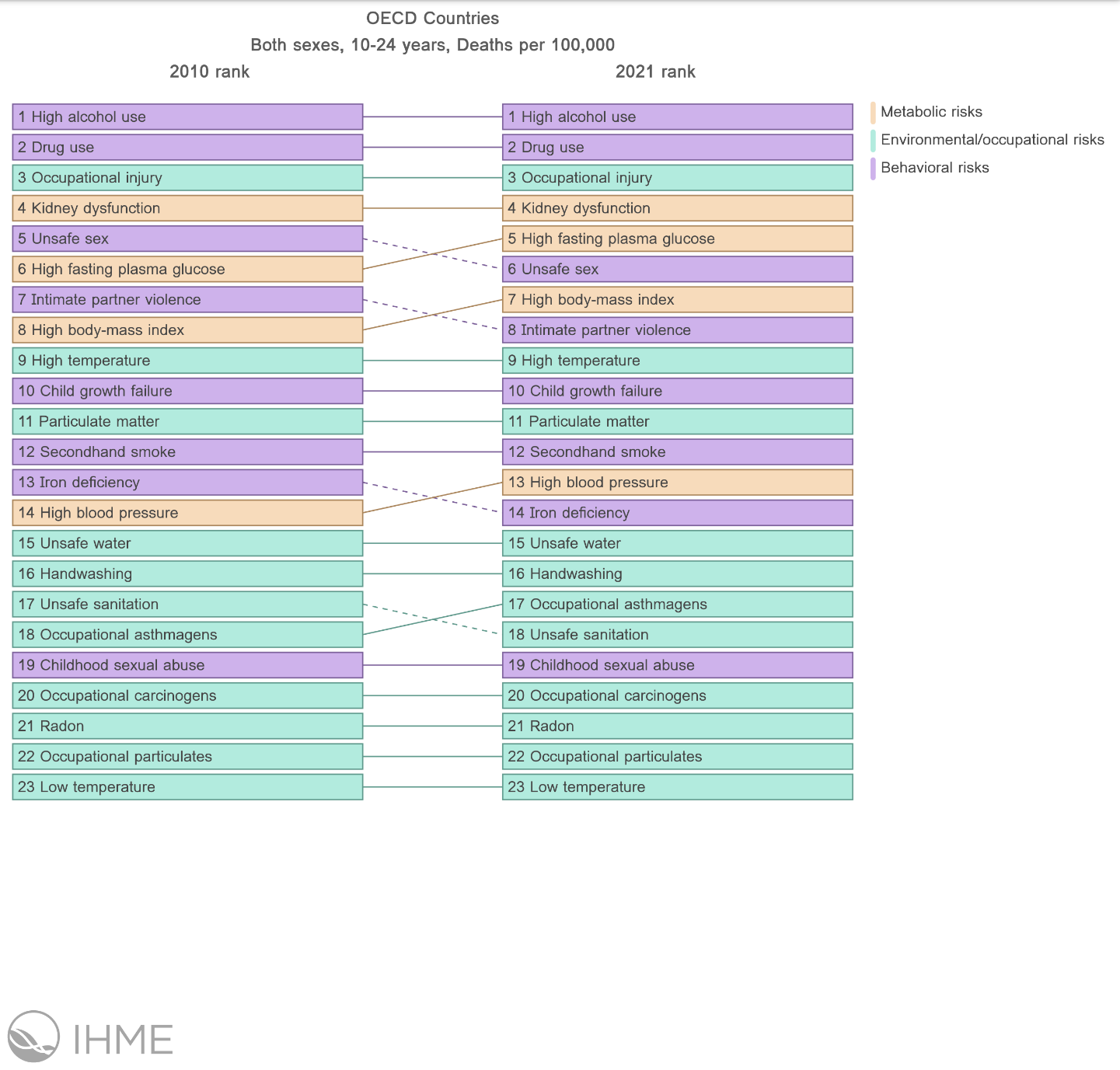

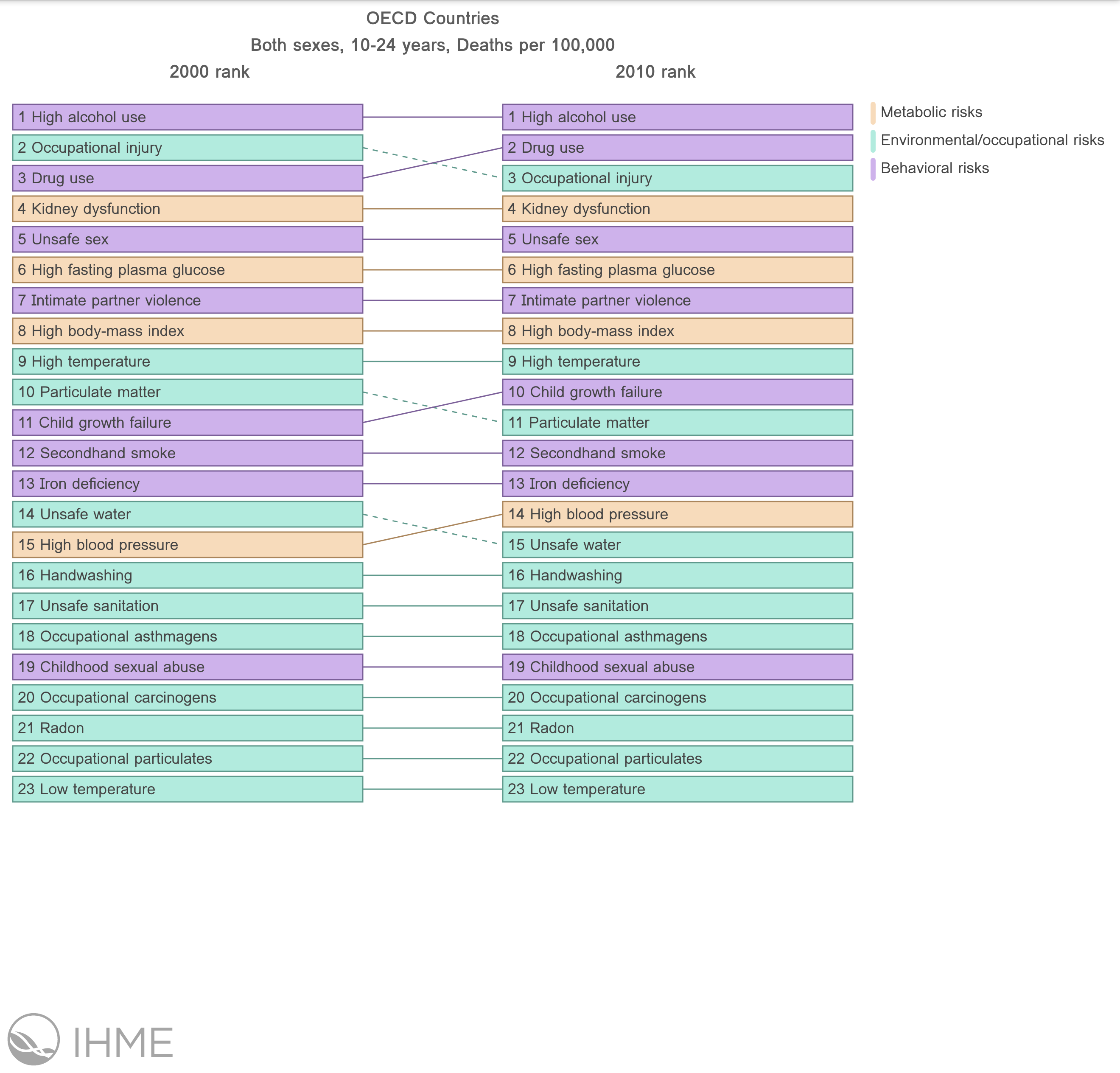


1. 10- 14 years


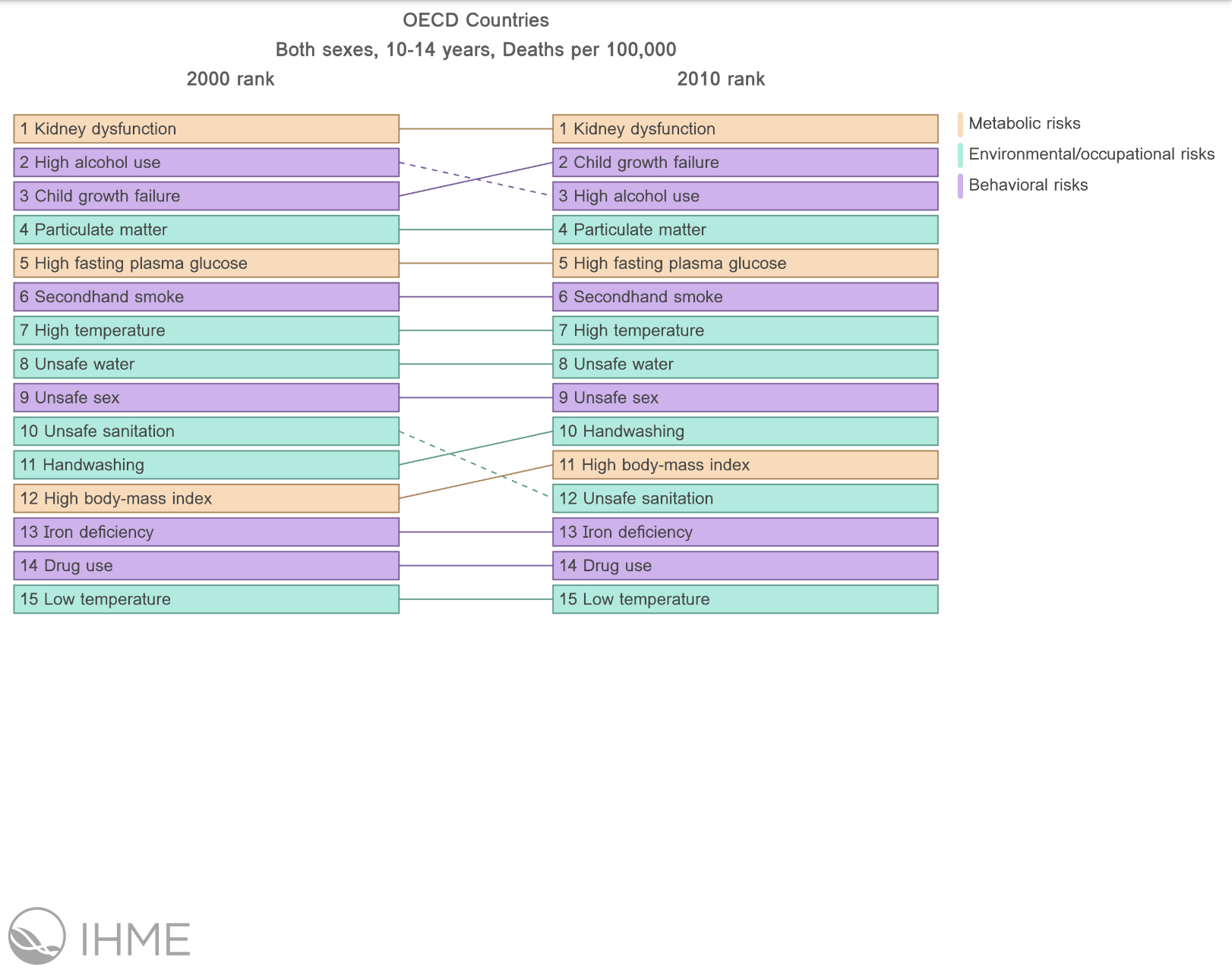

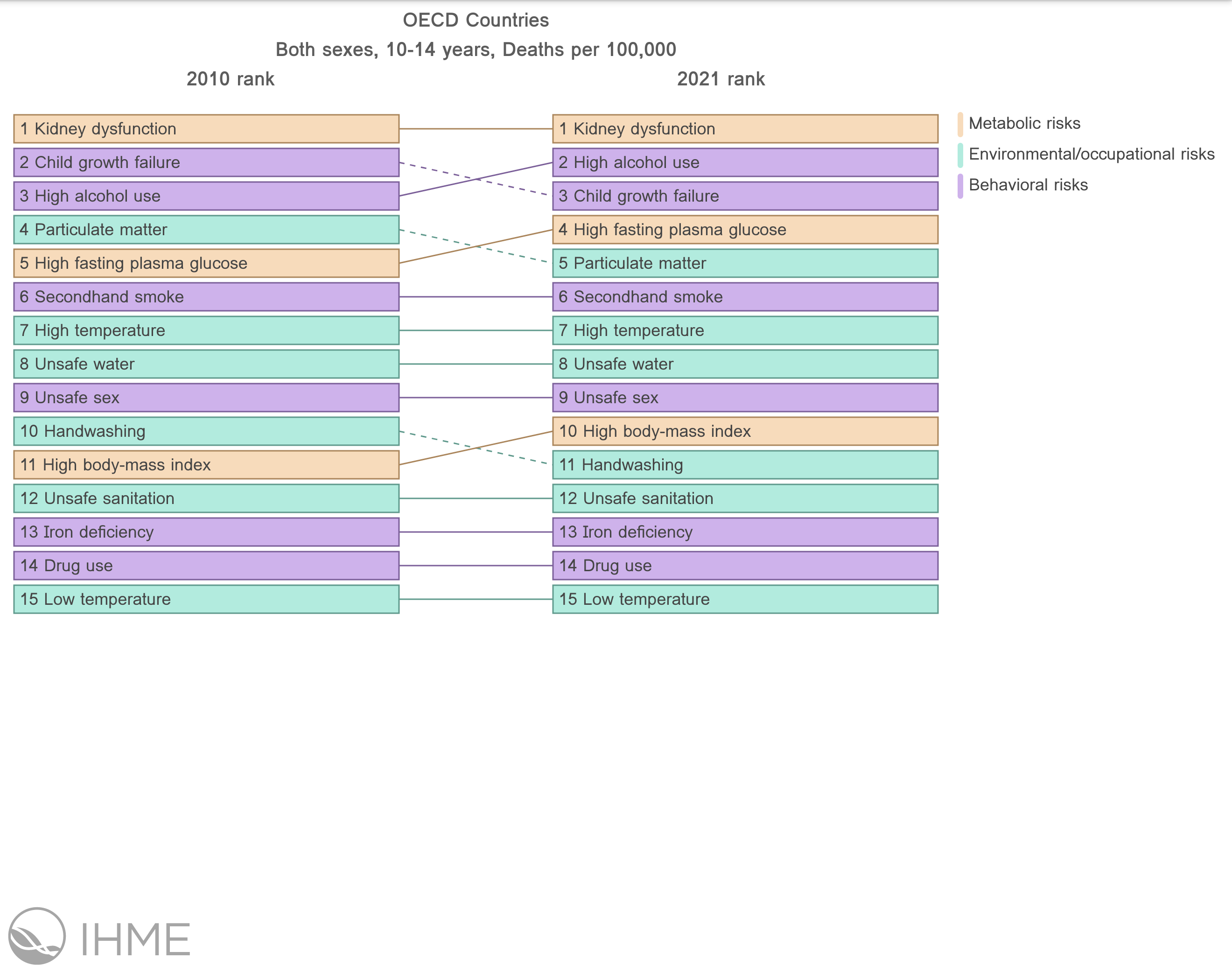


1. 15-19 years


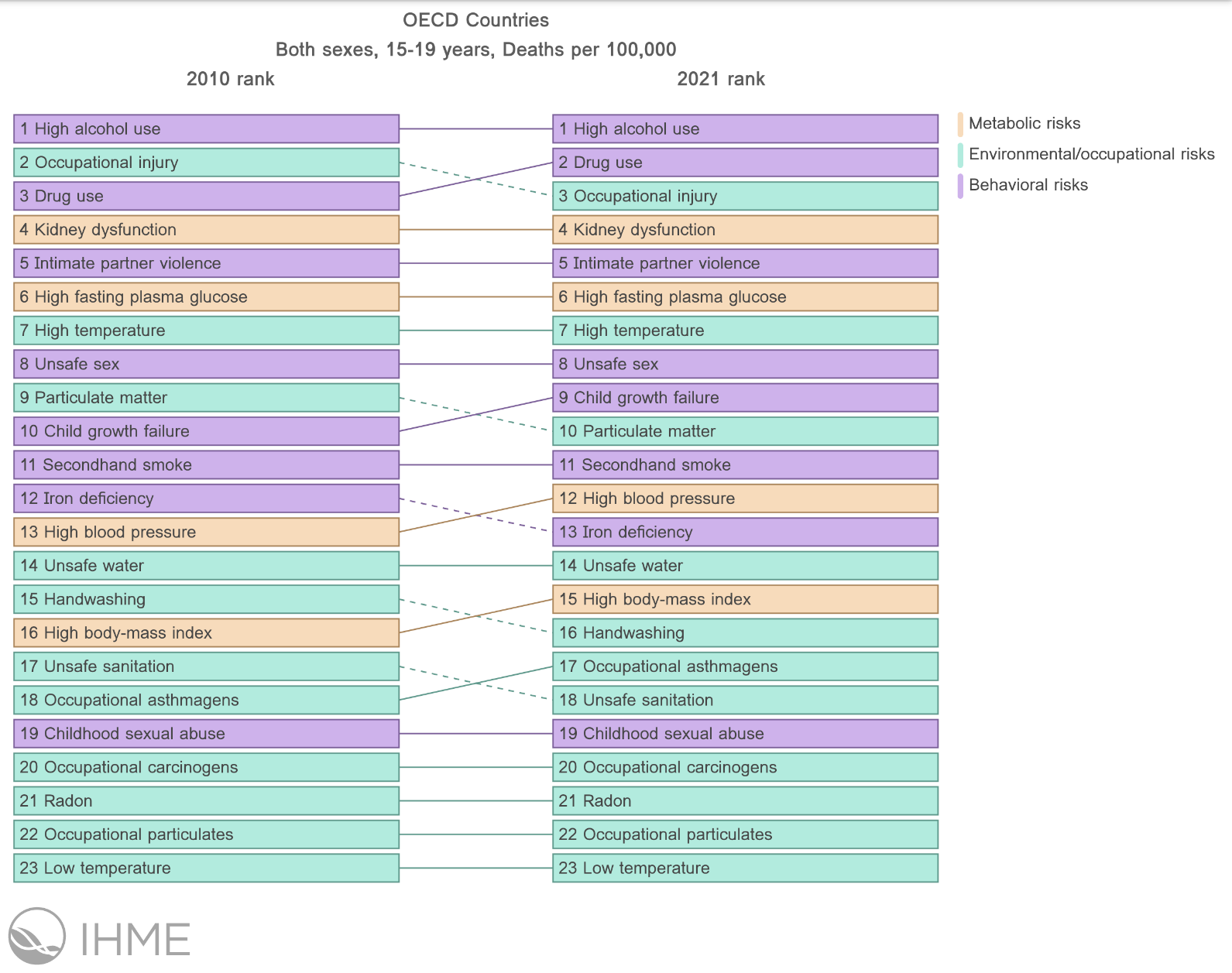

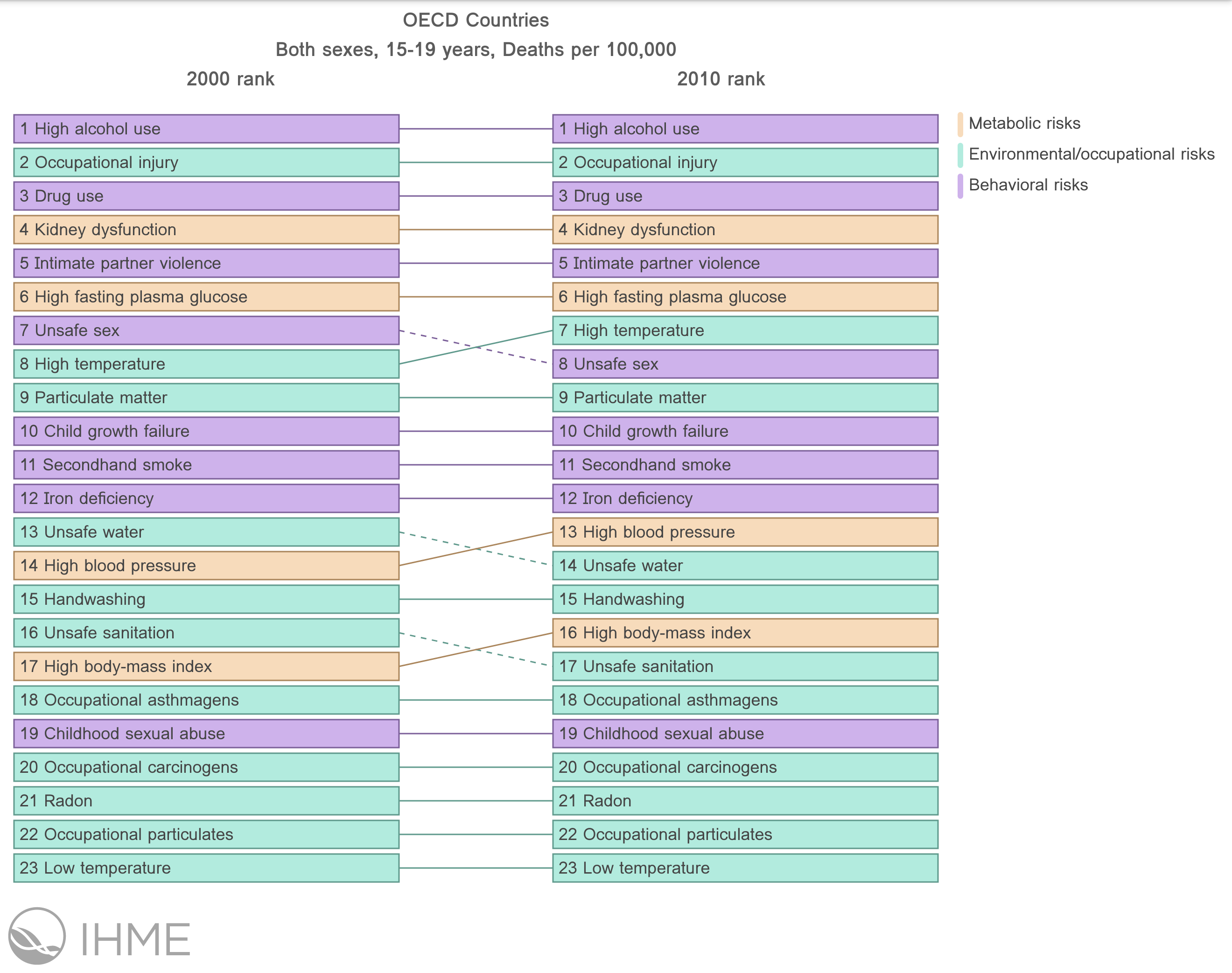


1. 20-24 years


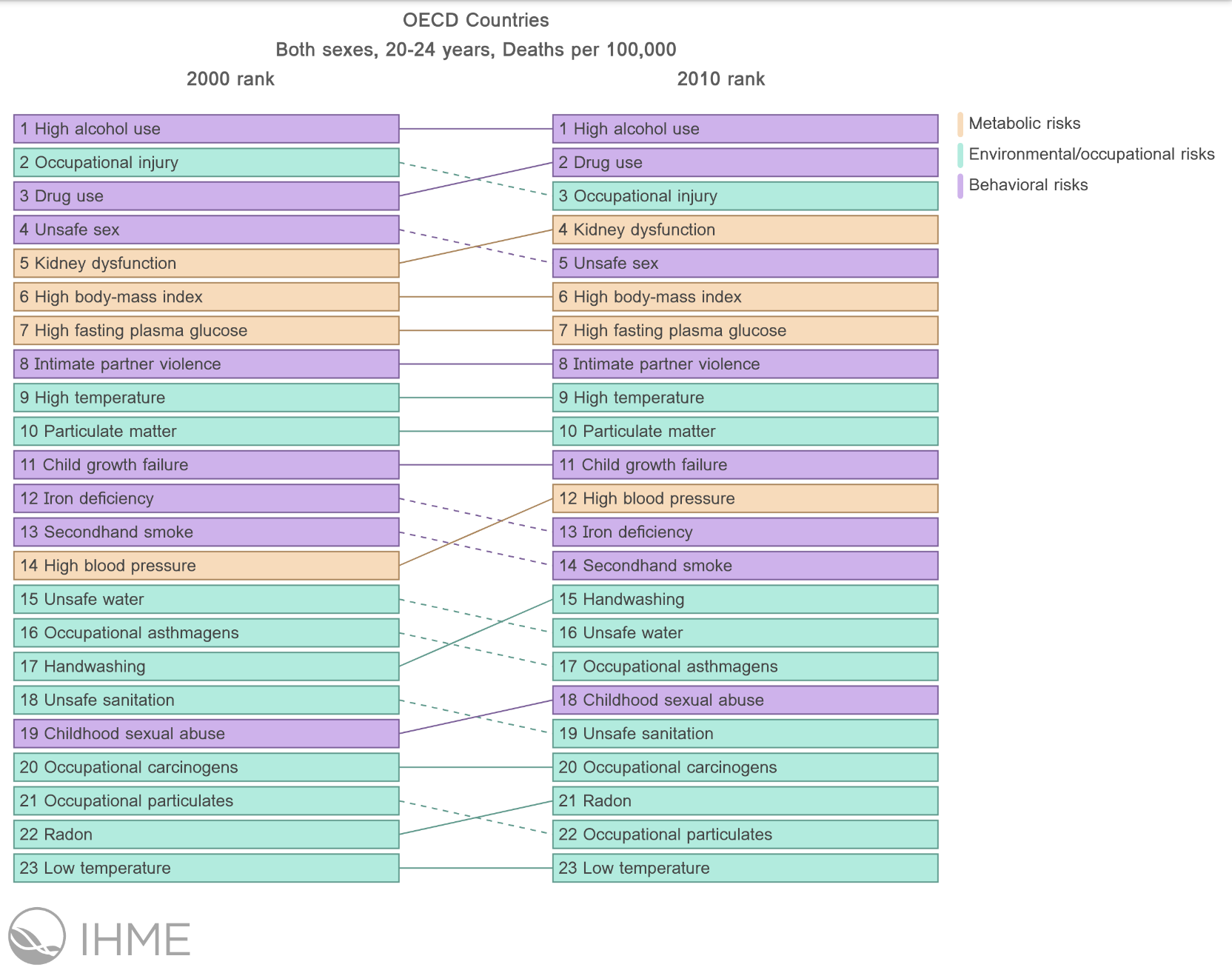

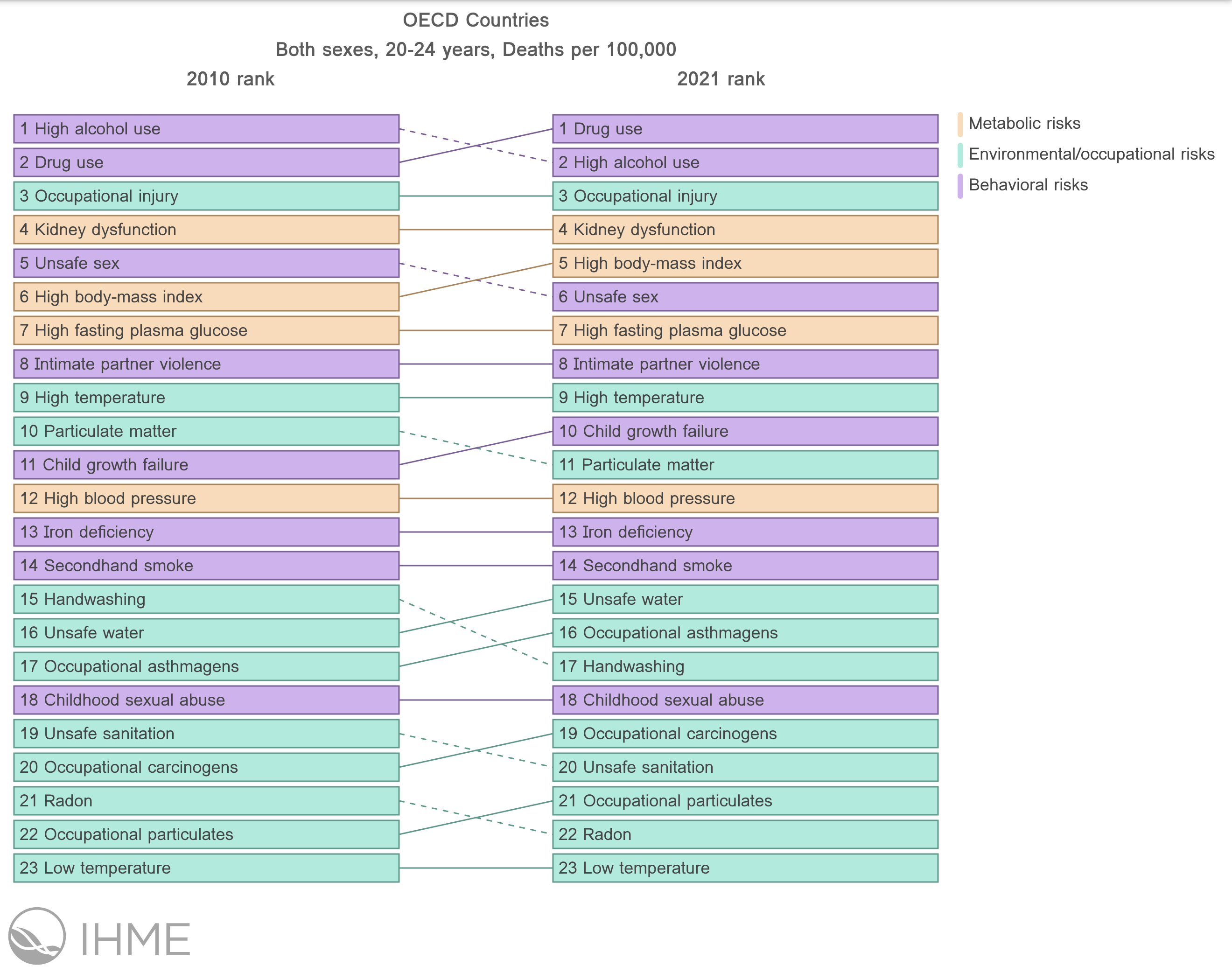


#### Figure S3: Top 15 risk factors of death for male adolescents and young adults (10-24 years) in OECD Countries in the years 2000, 2010, and 2021

1. 10-24 years


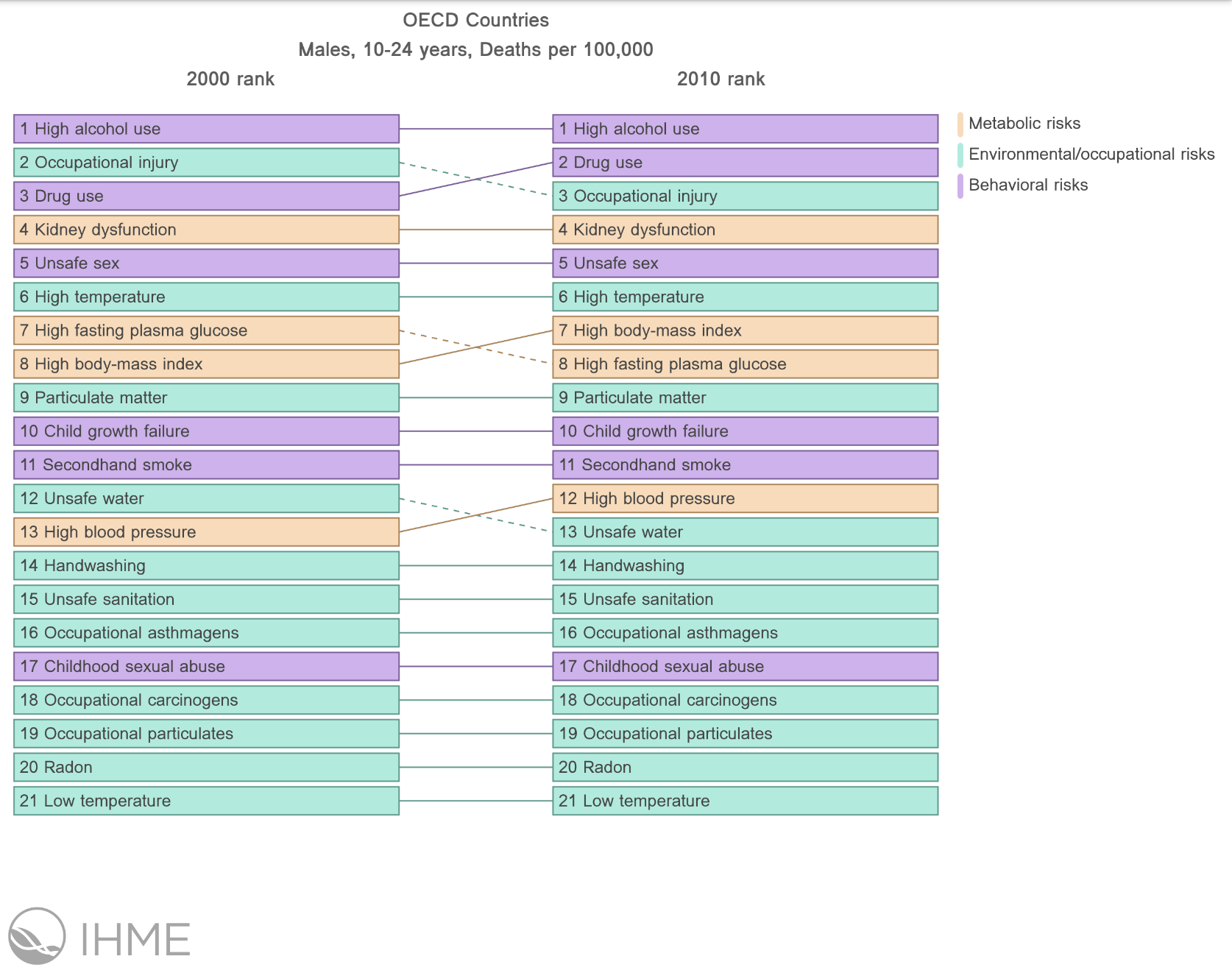

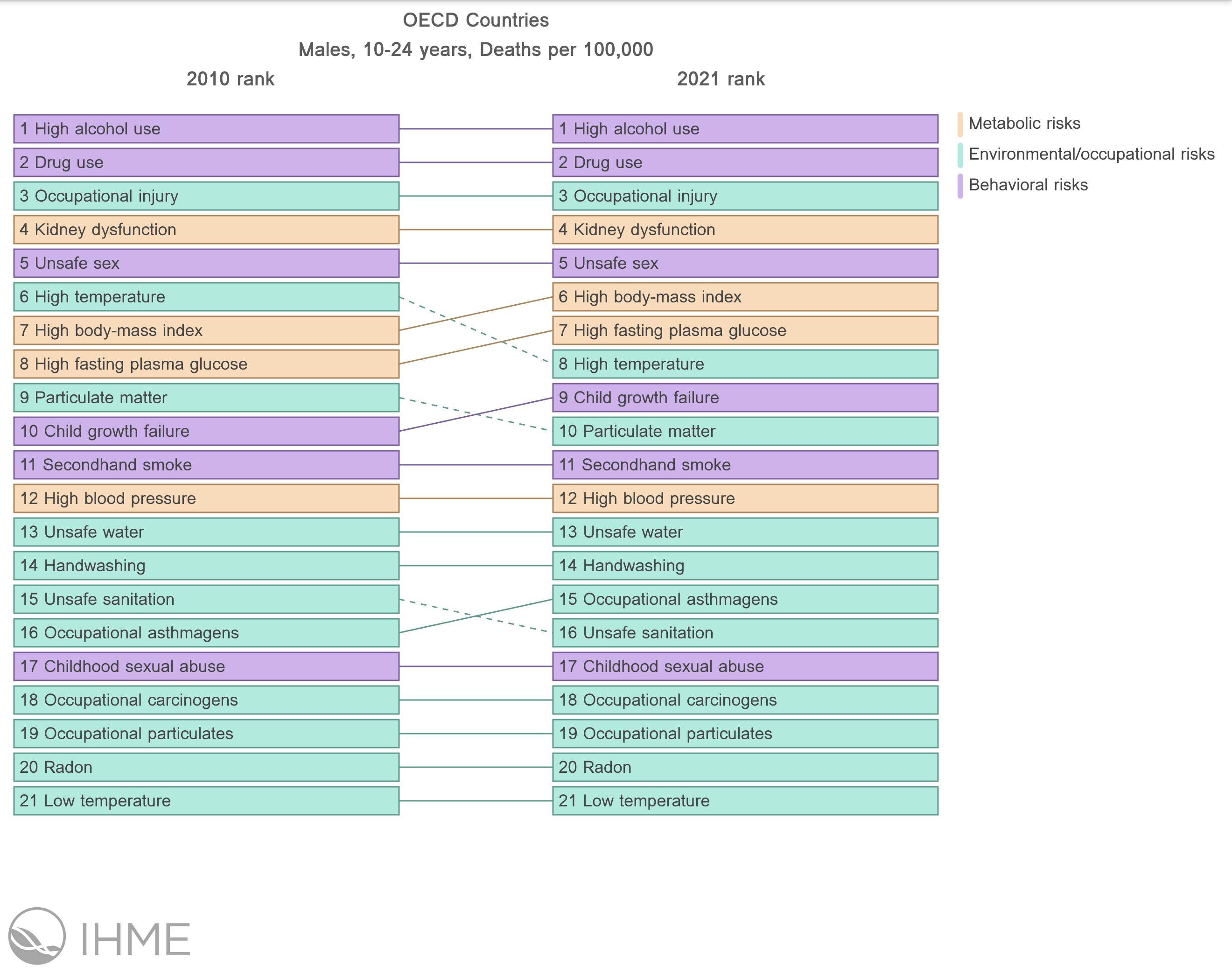


1. 10- 14 years


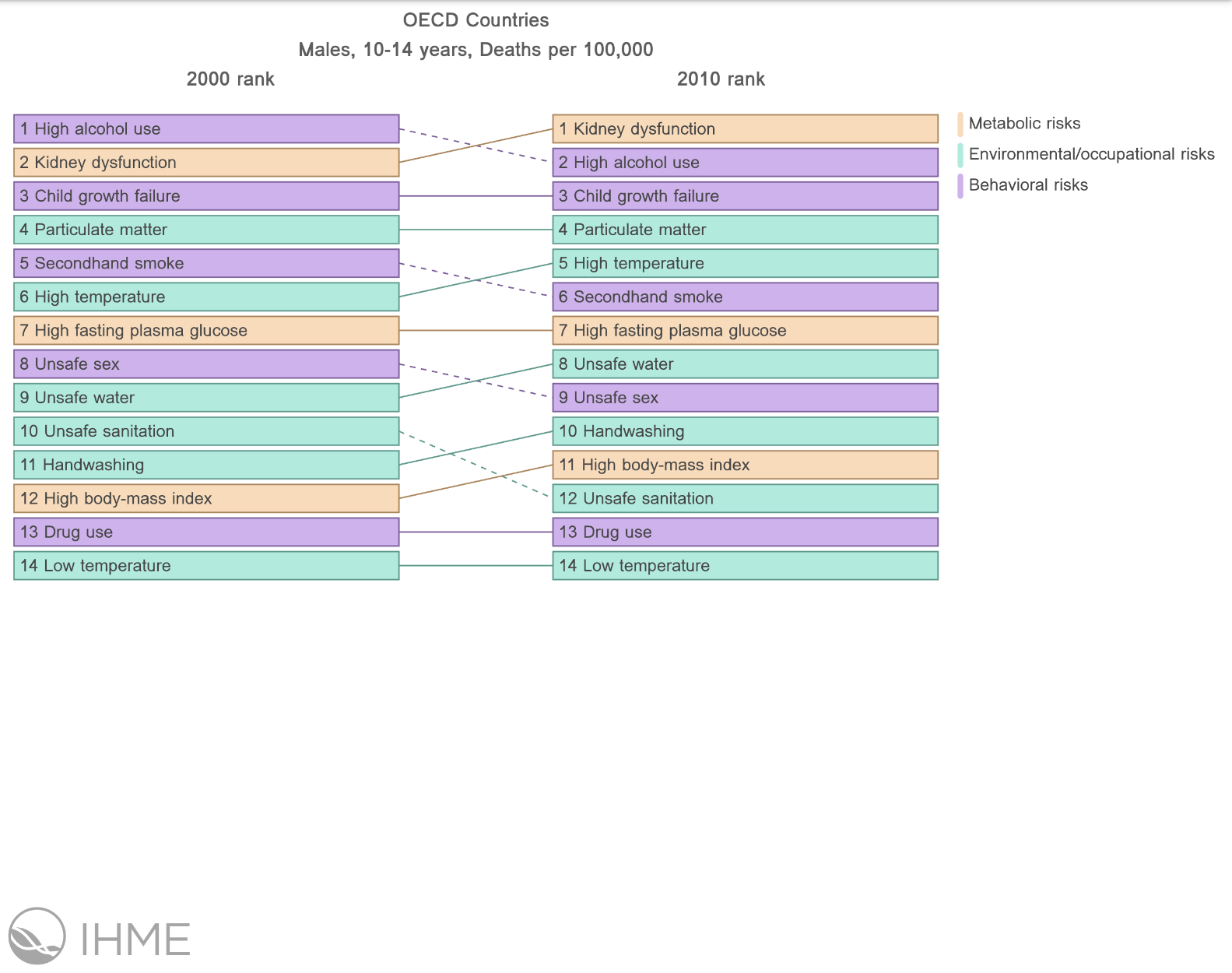

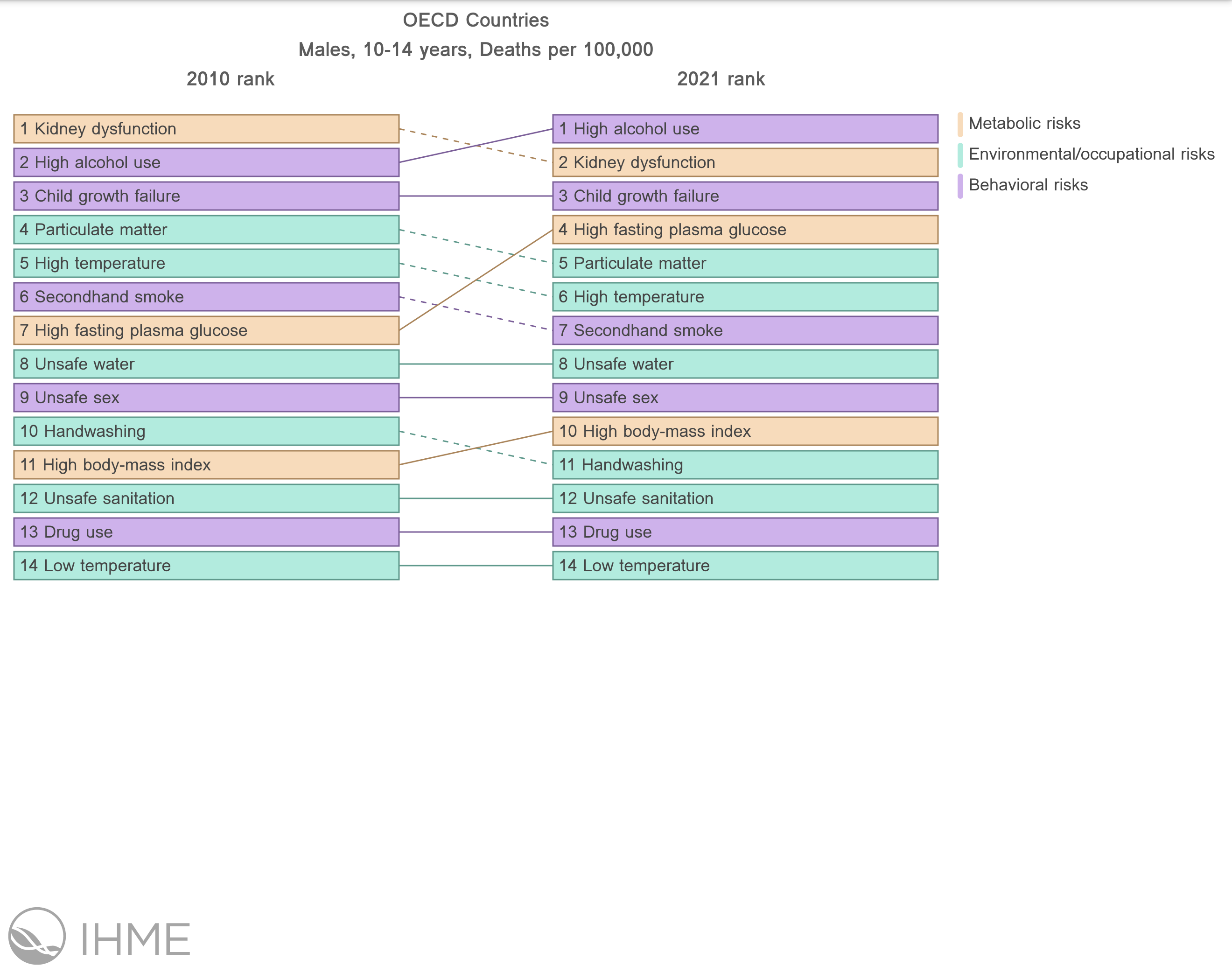


1. 15-19 years


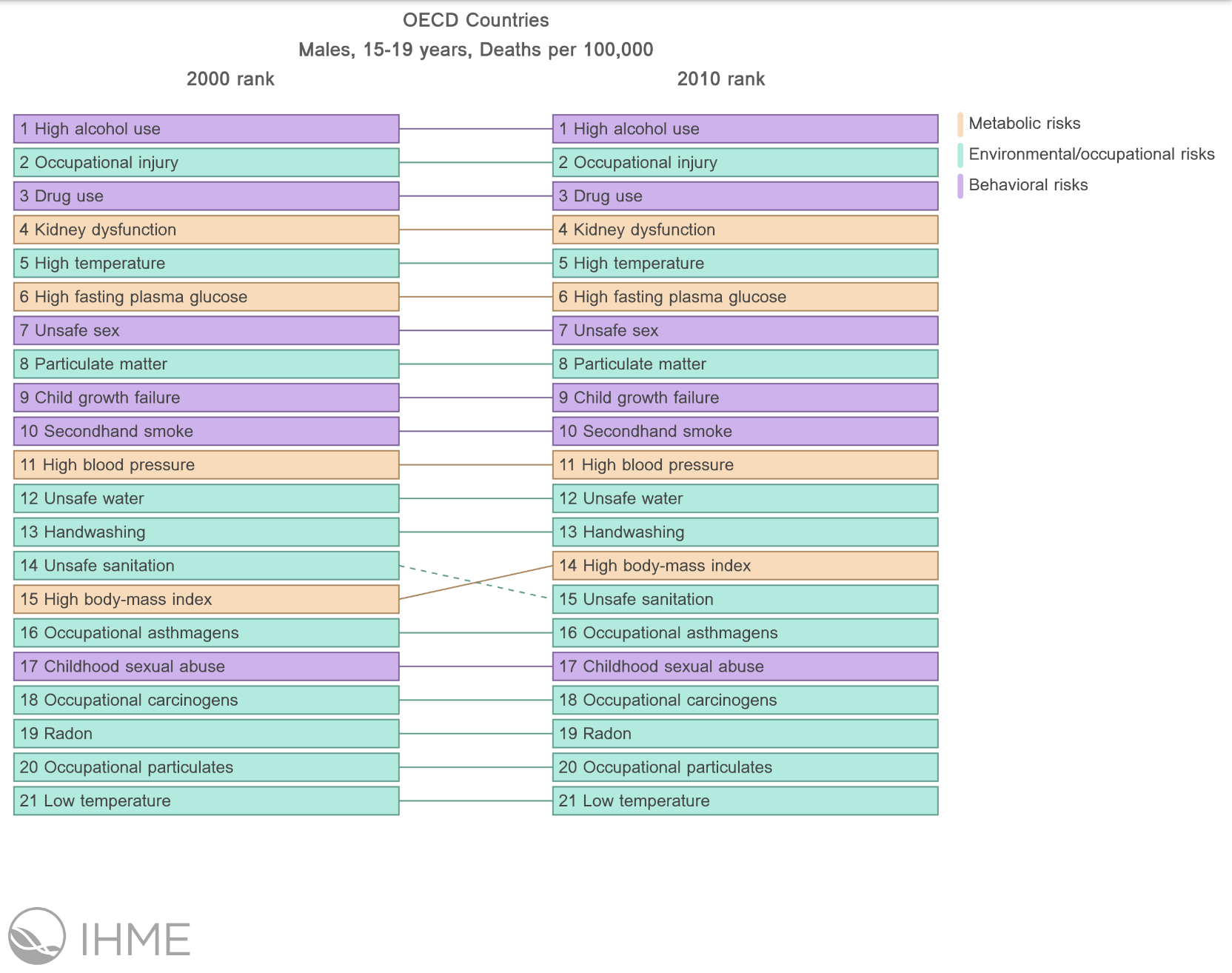

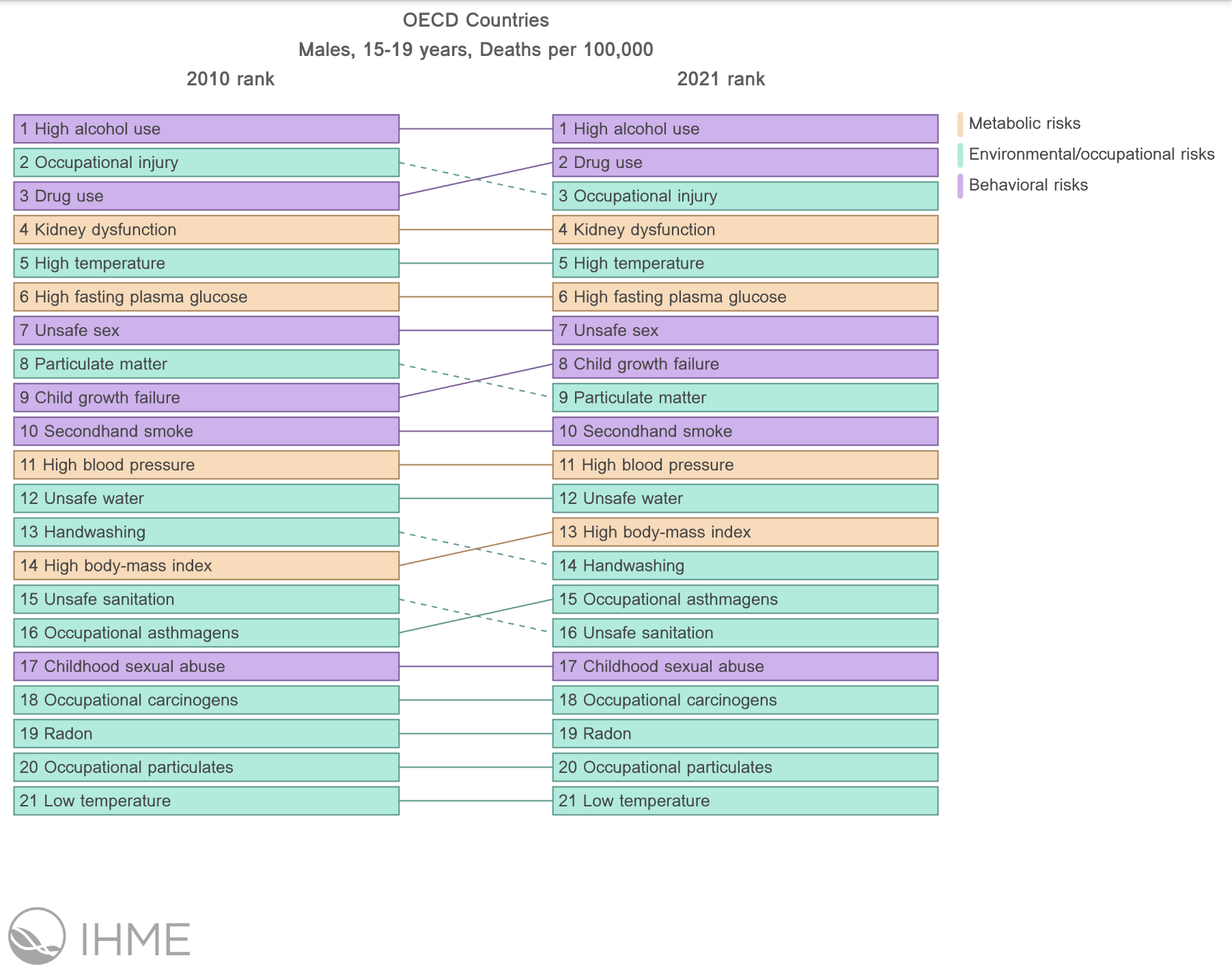


1. 20-24 years


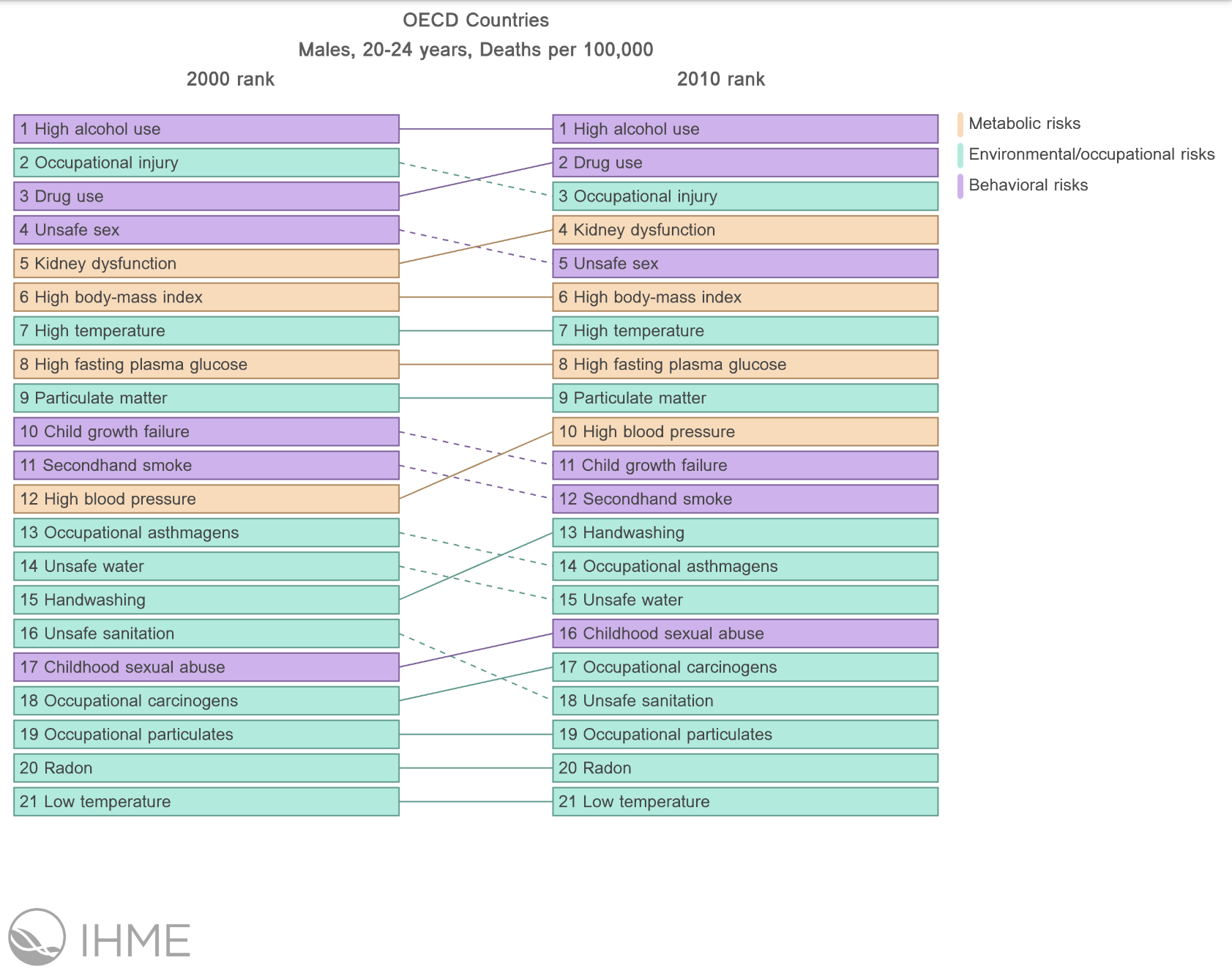

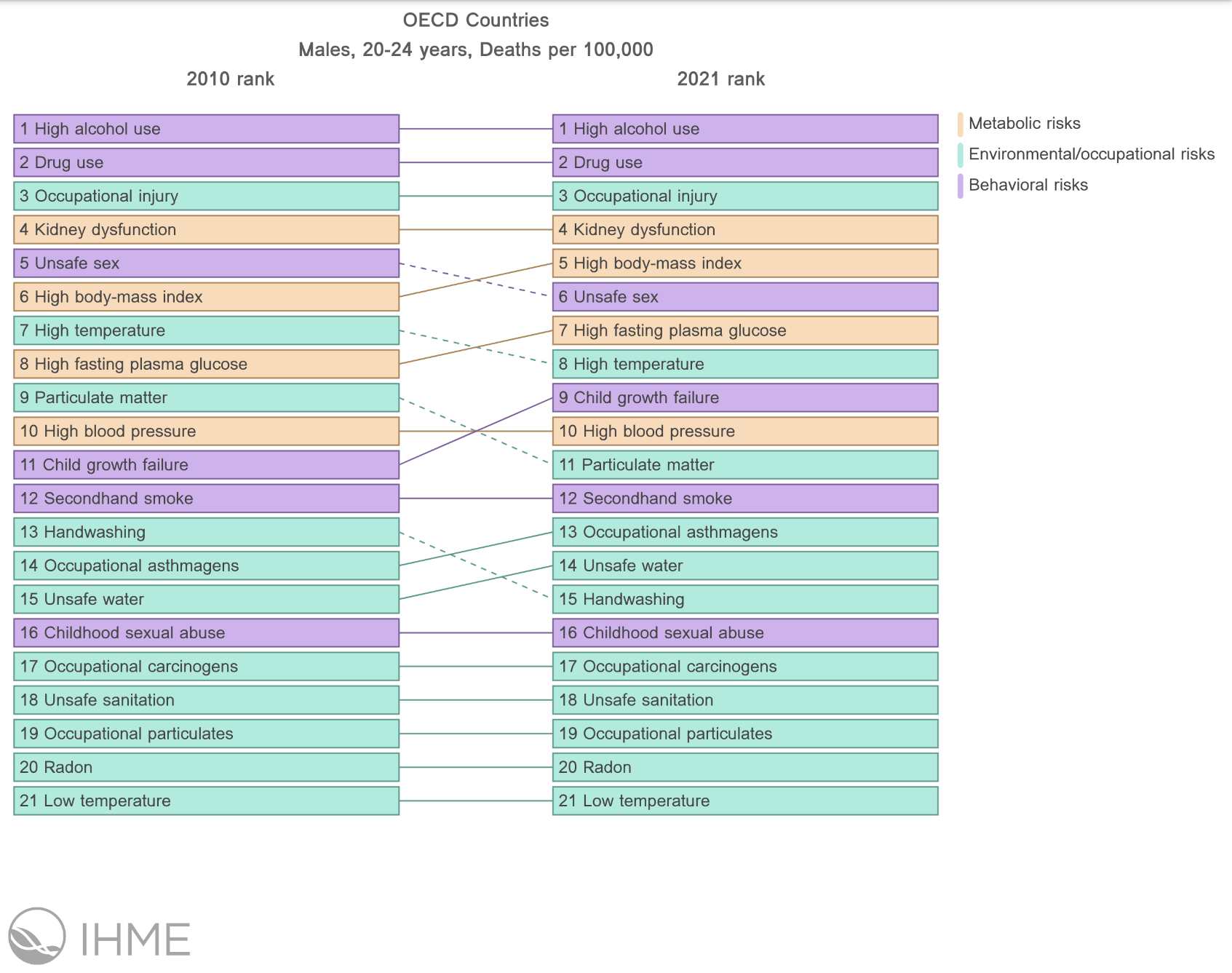


#### Figure S4: -Top 15 risk factors of death for male adolescents and young adults (10-24 years) in Australia in the years 2000, 2010, and 2021

1. 10-24 years


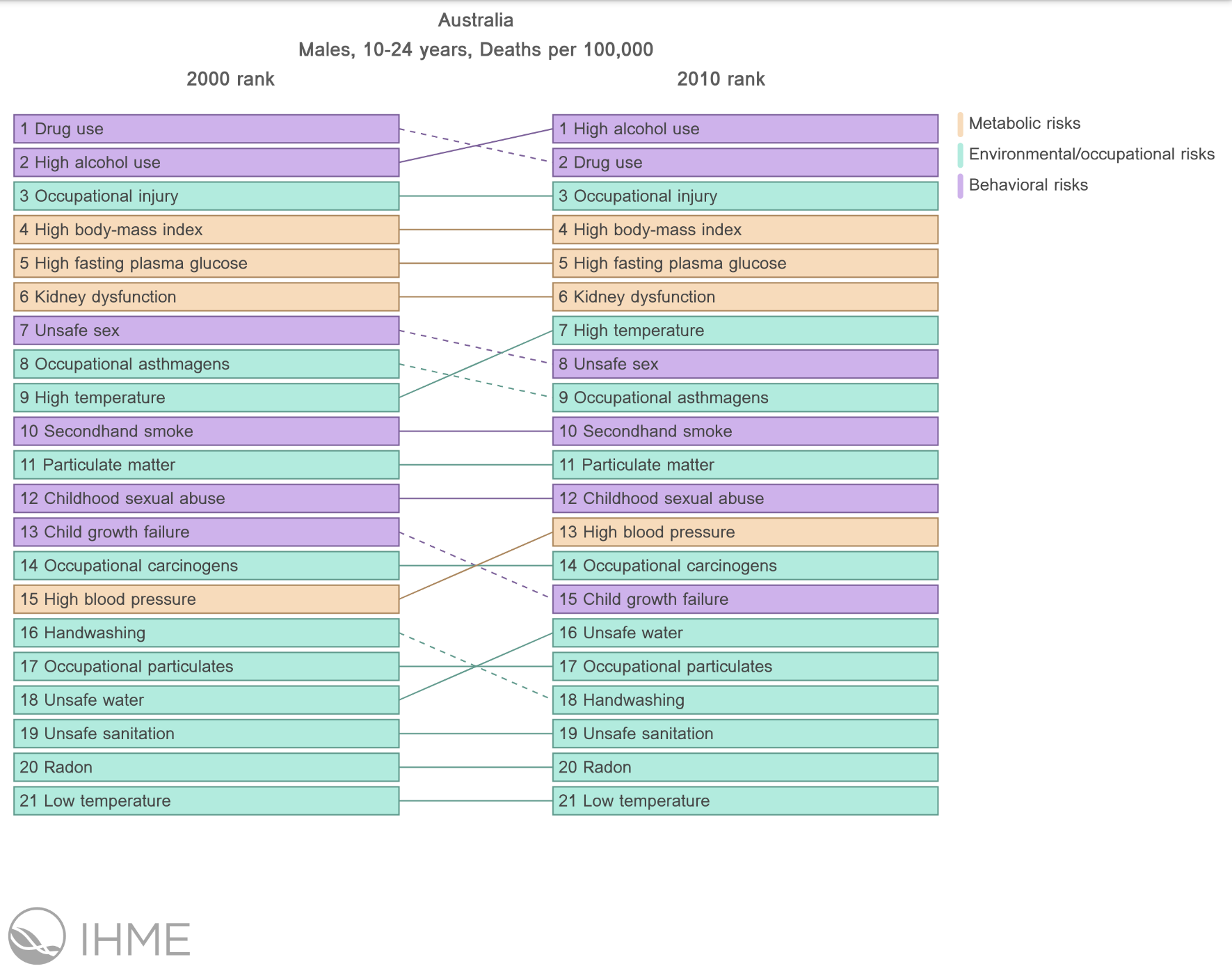

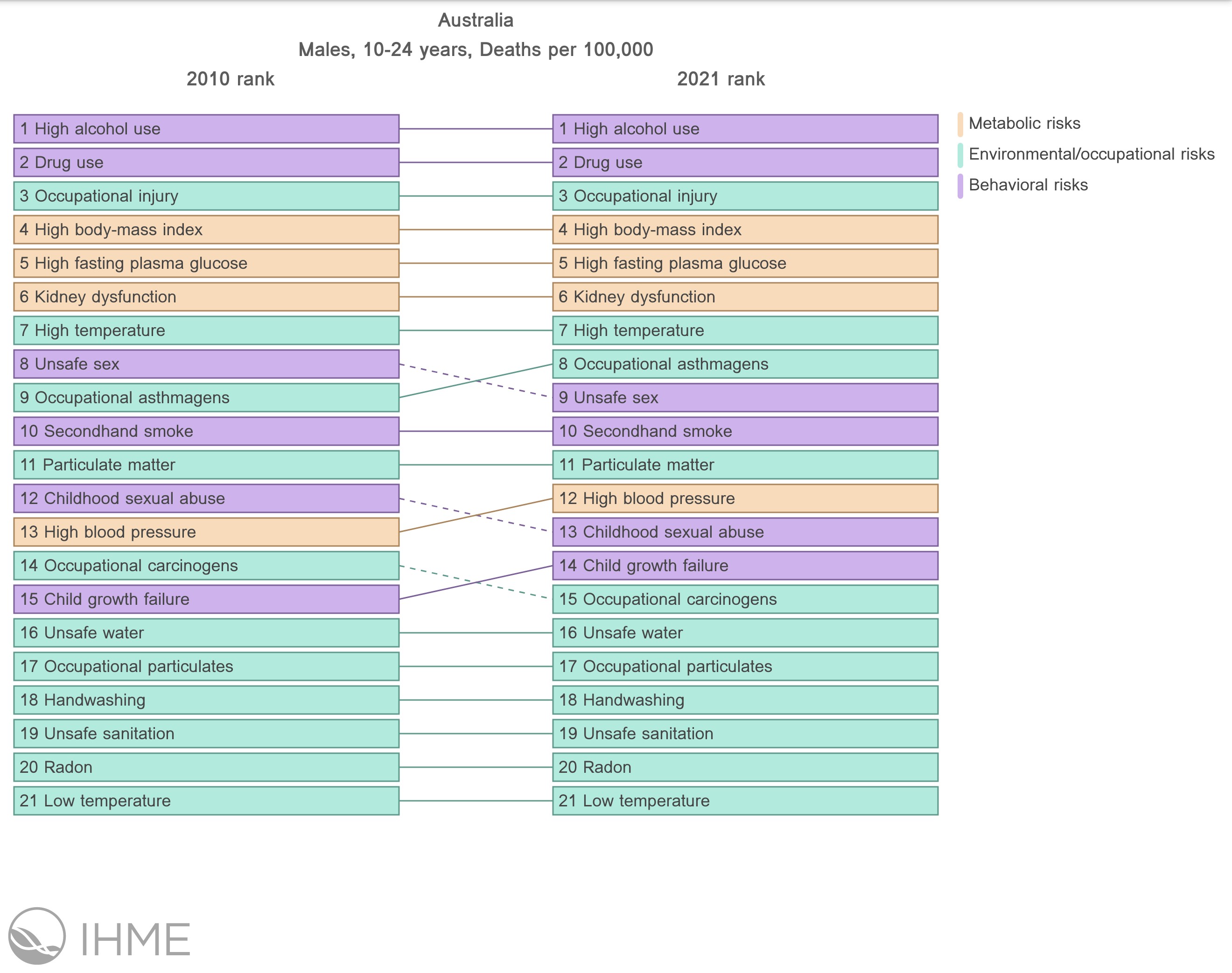


1. 10- 14 years


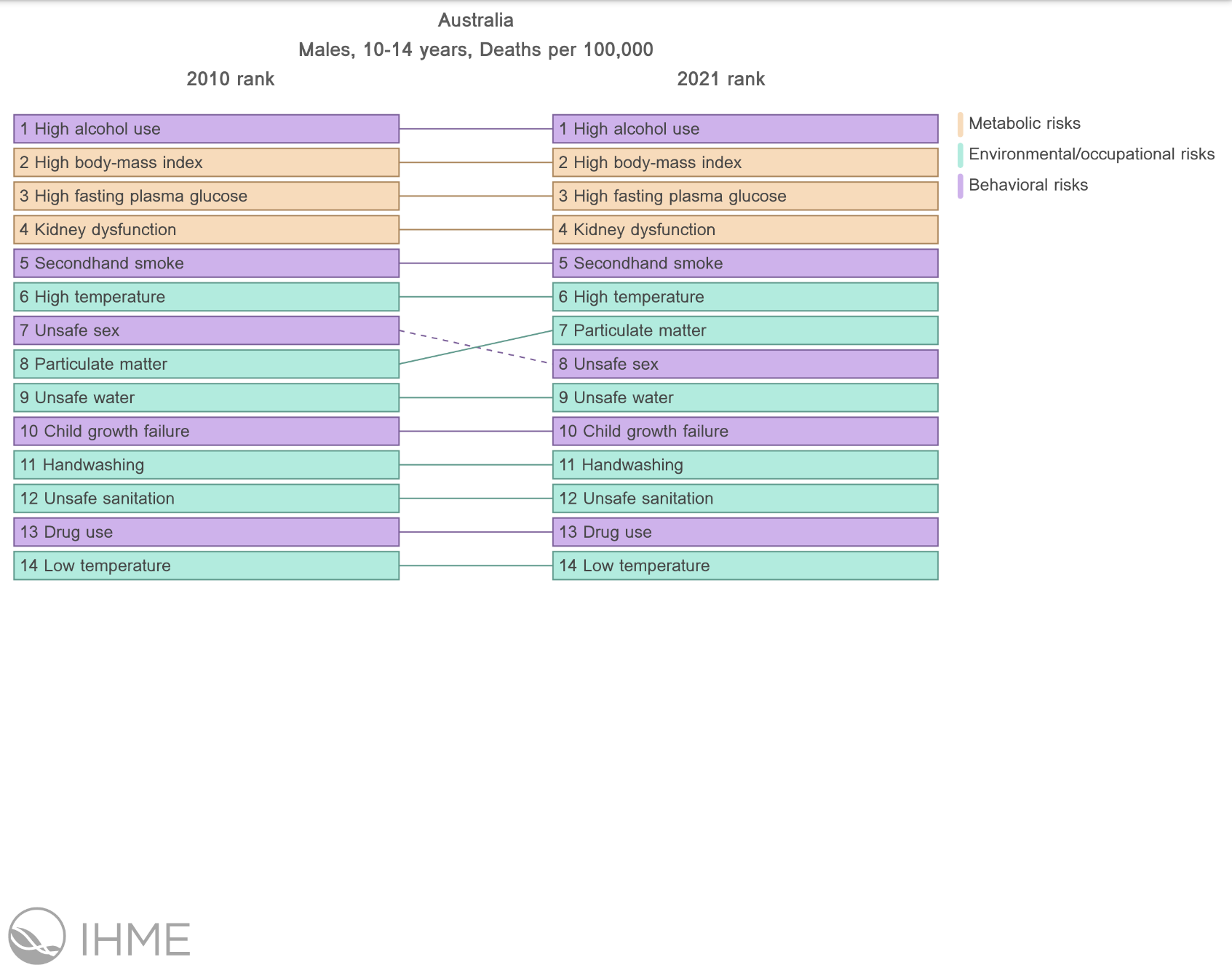

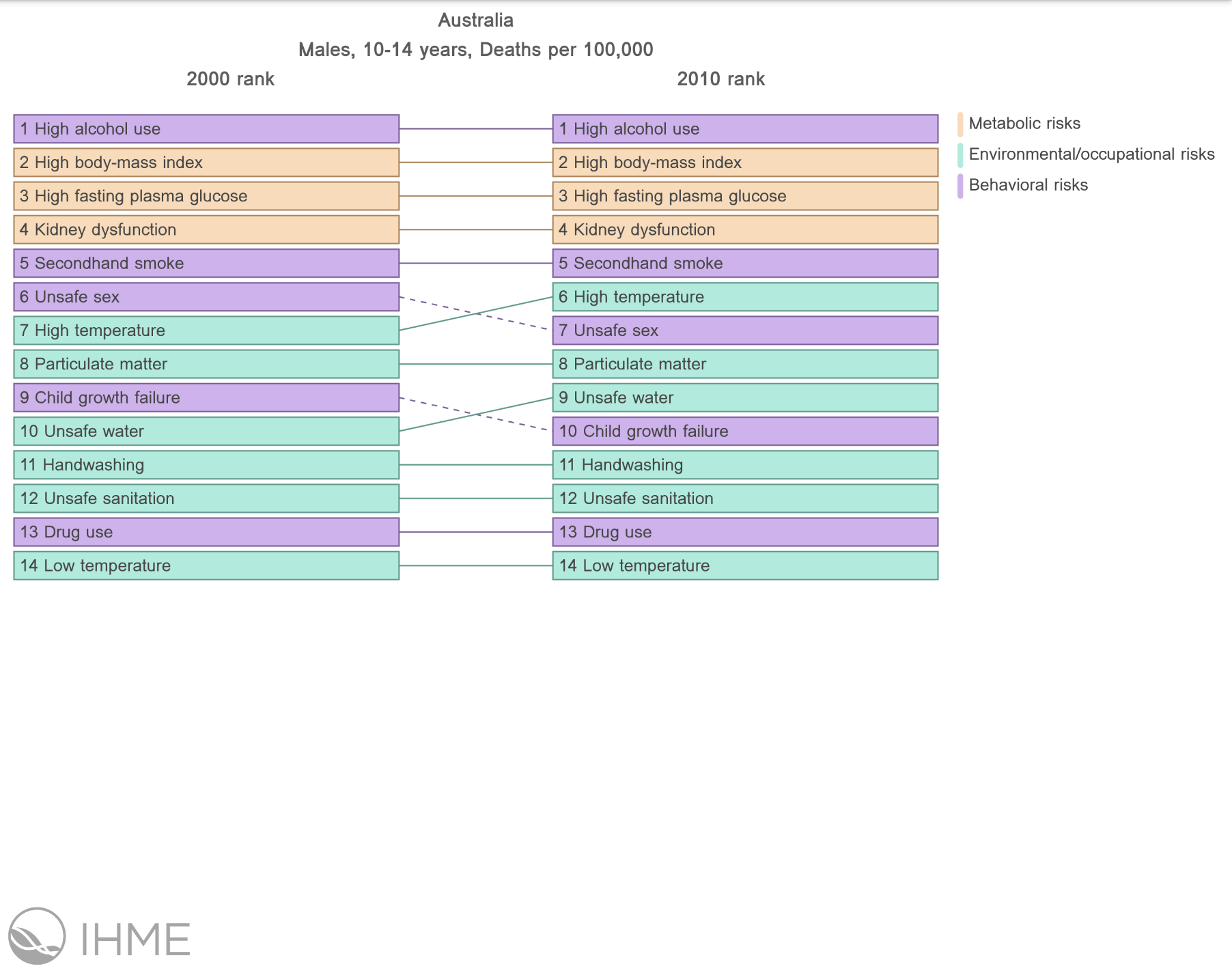


1. 15-19 years


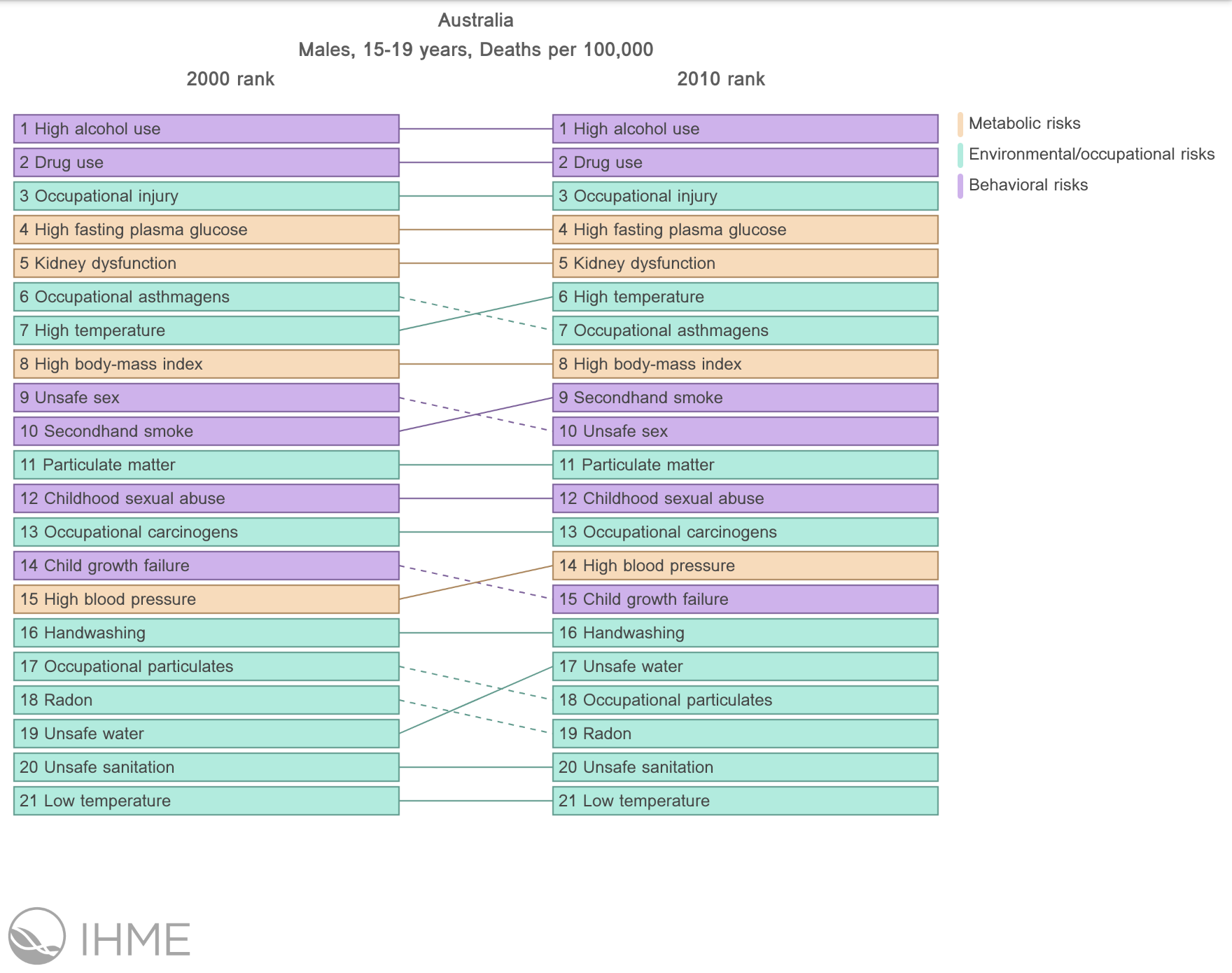

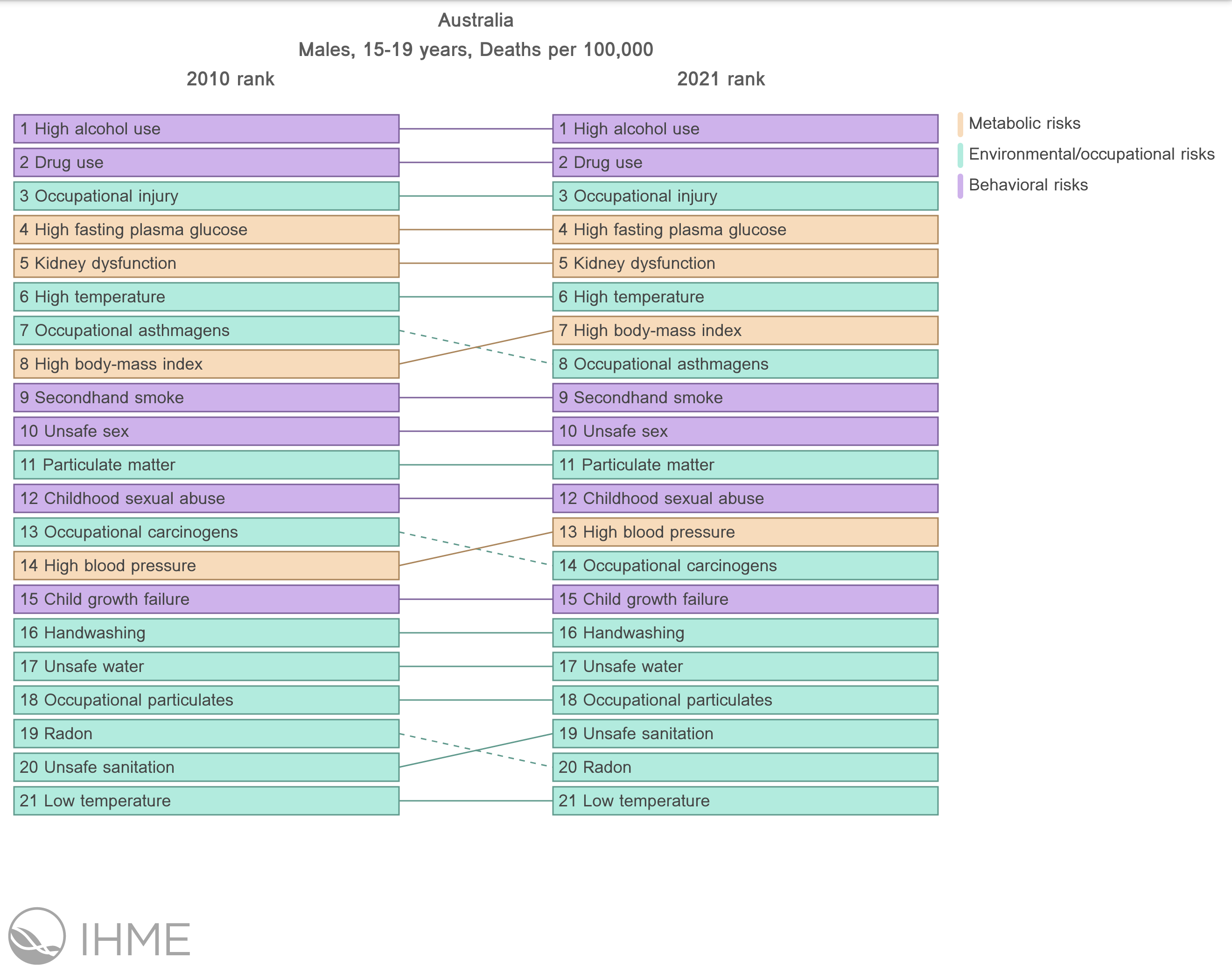


1. 20-24 years


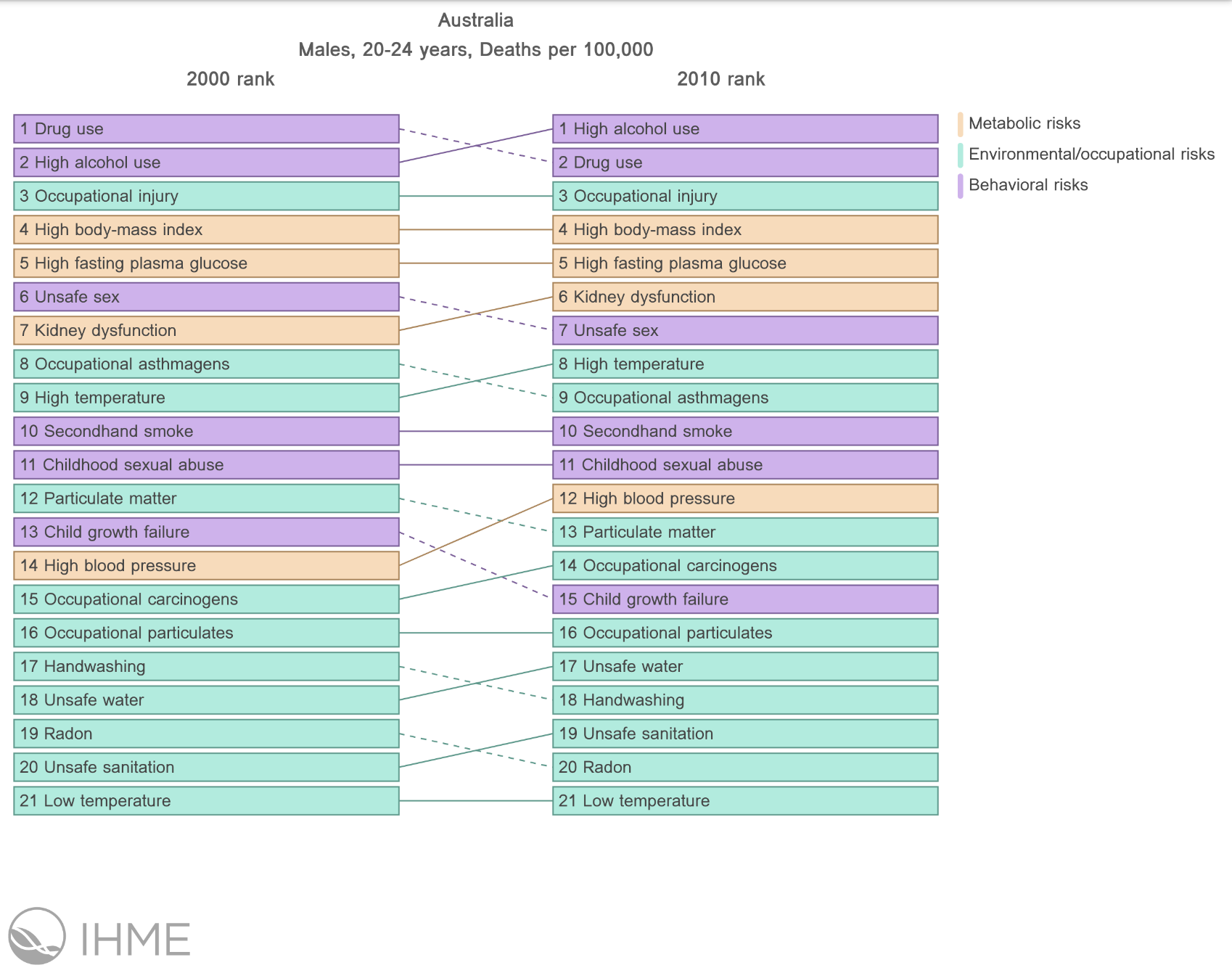

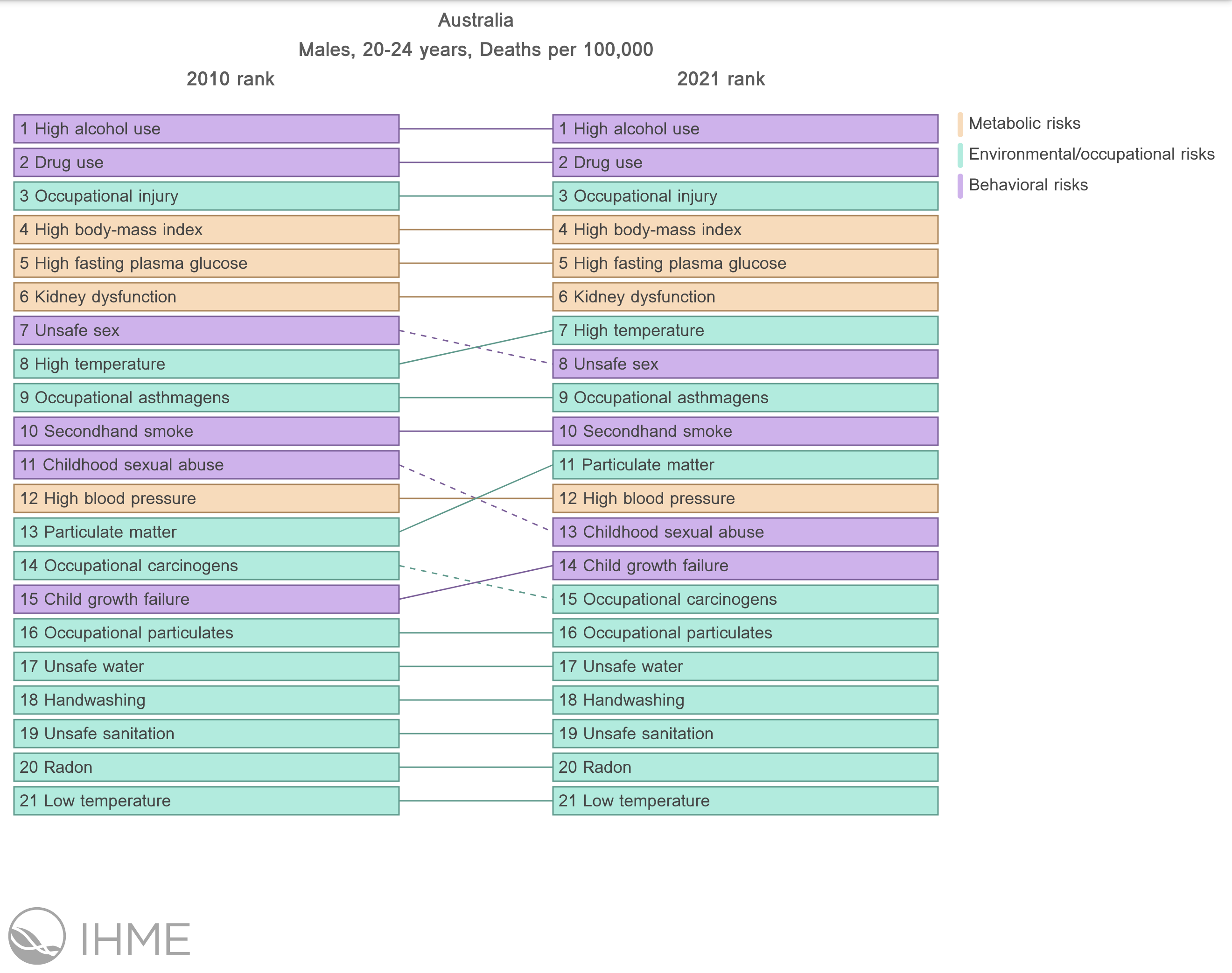


#### Figure S5: -Top 15 risk factors of death for female adolescents and young adults (10-24 years) in OECD Countries in the years 2000, 2010, and 2021

1. 10-24 years


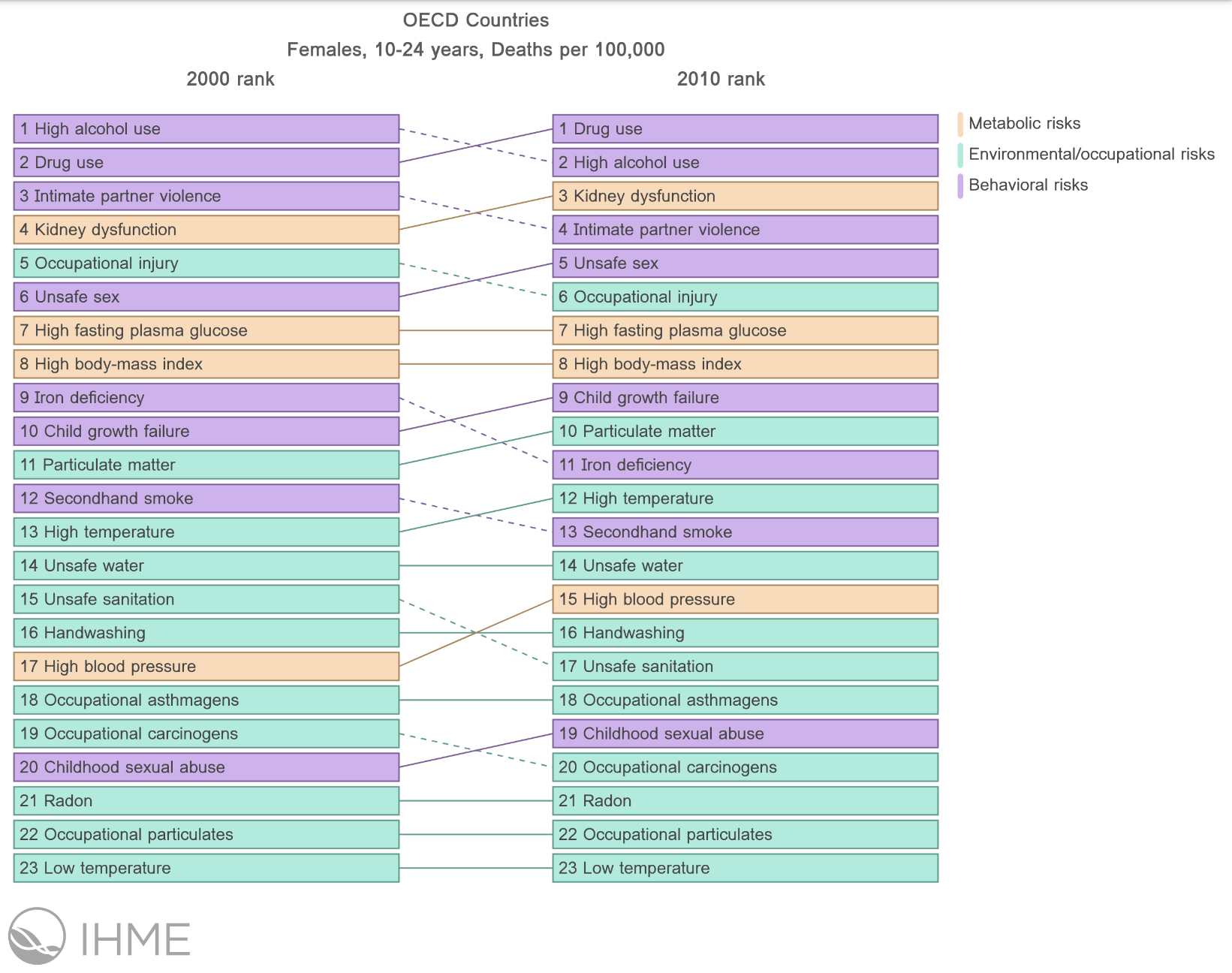

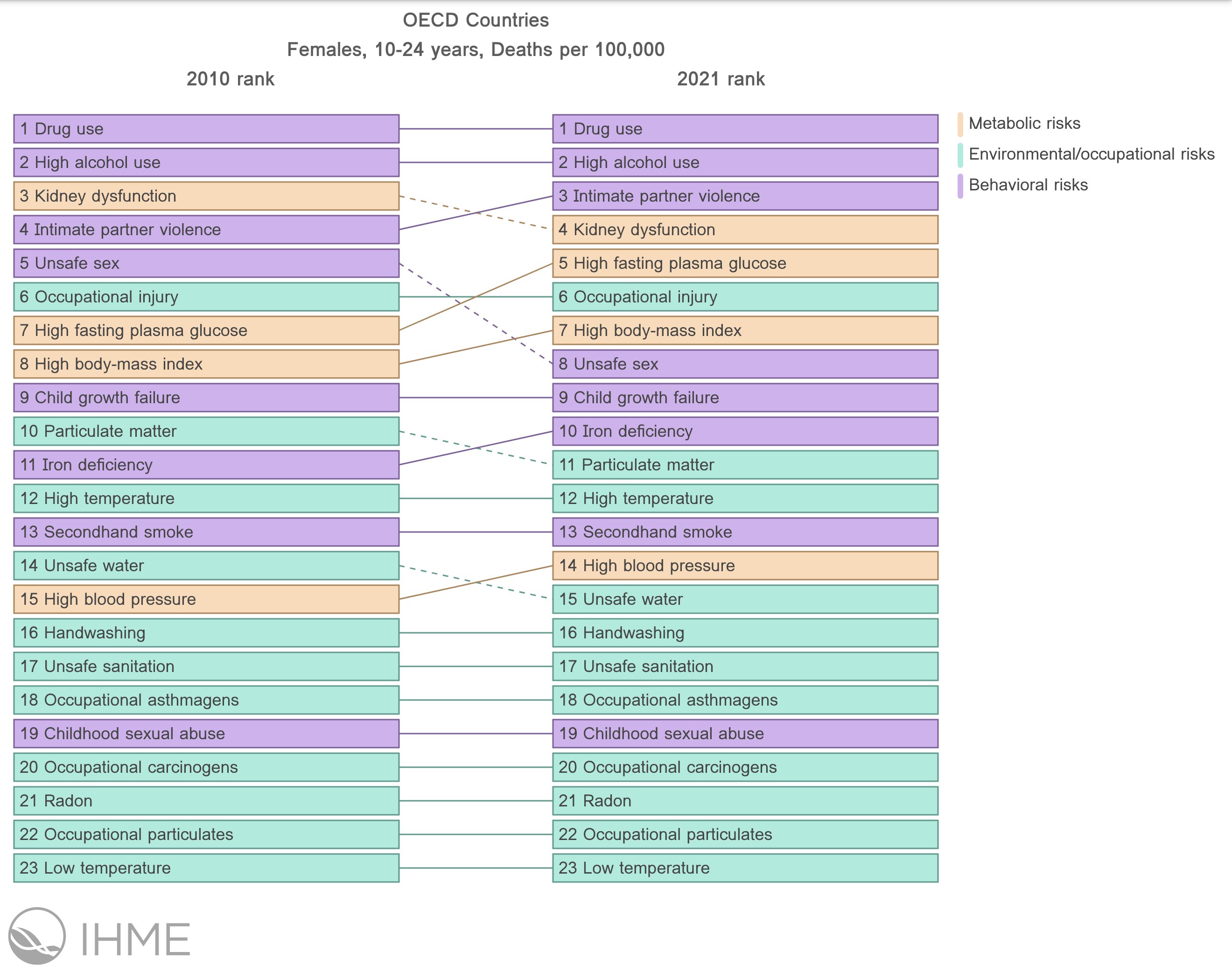


1. 10- 14 years


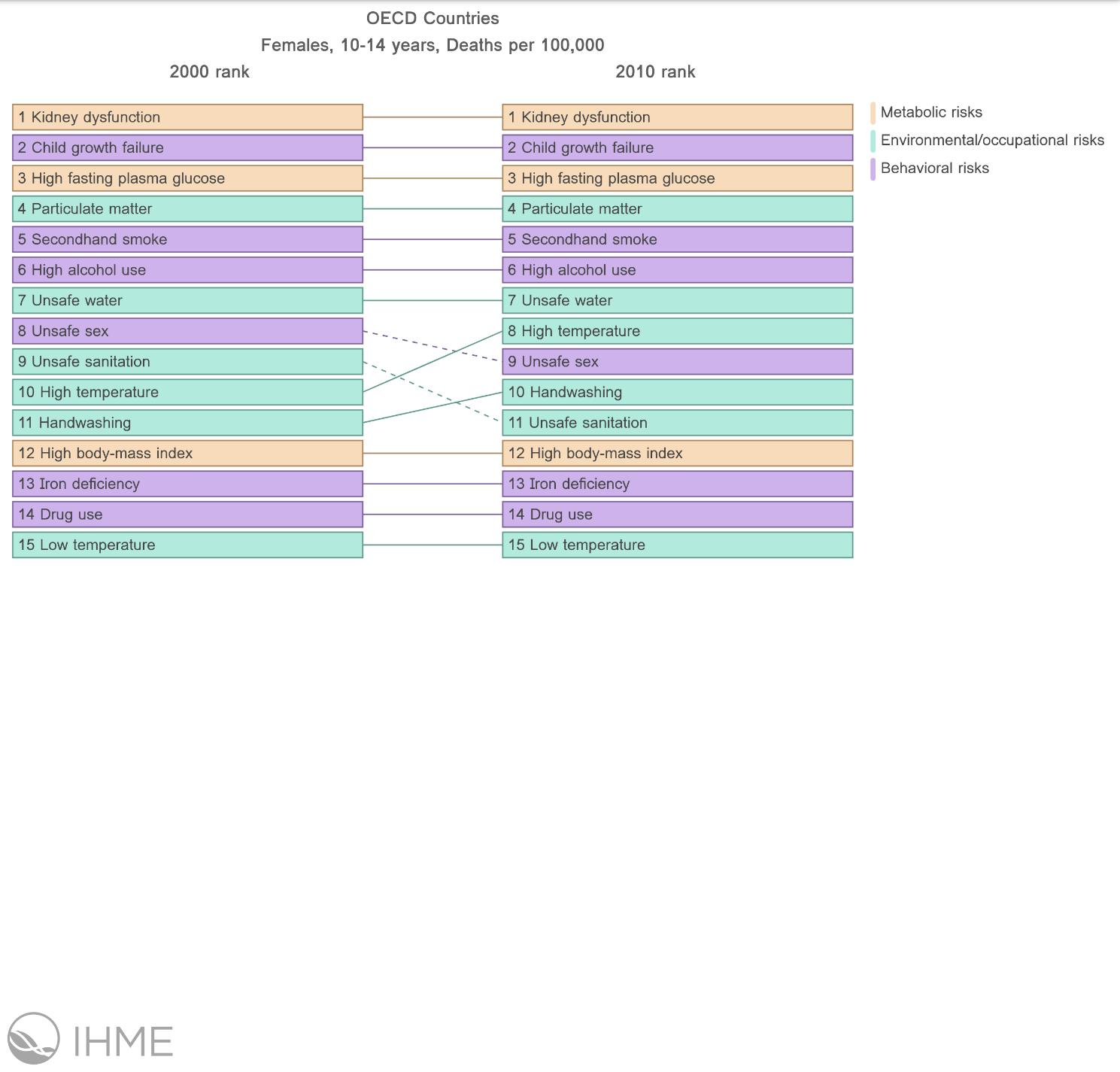

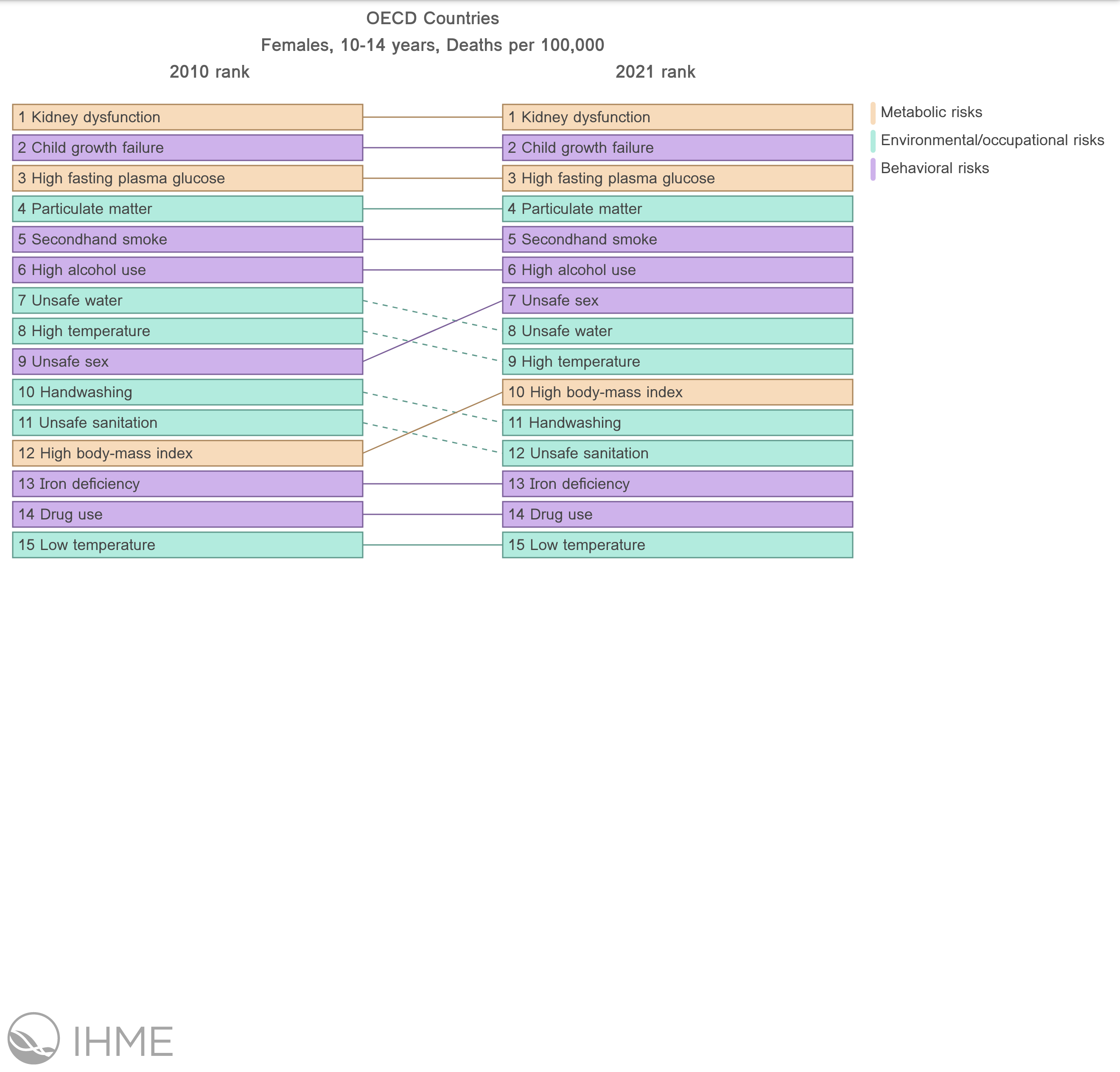


1. 15-19 years


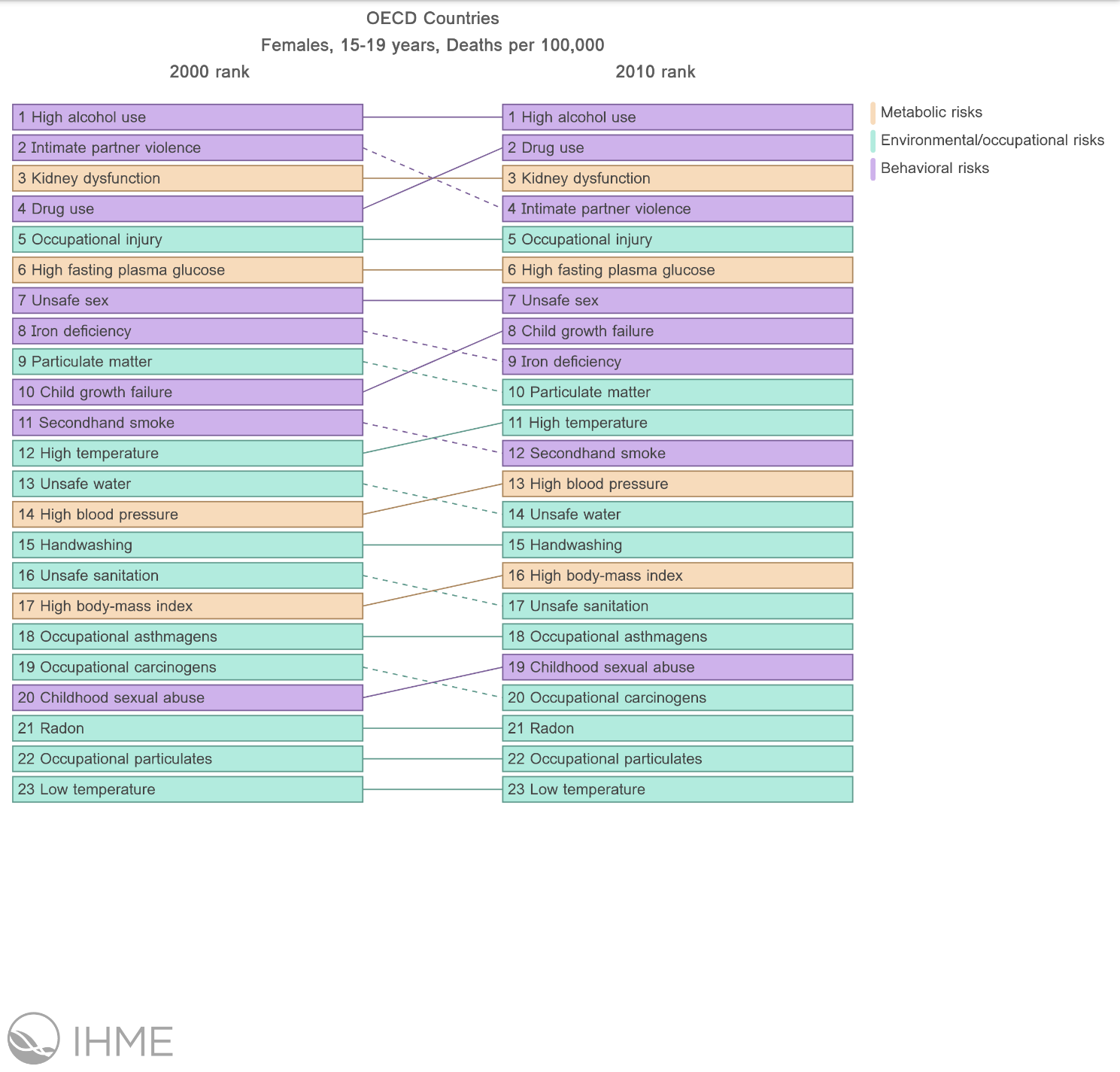

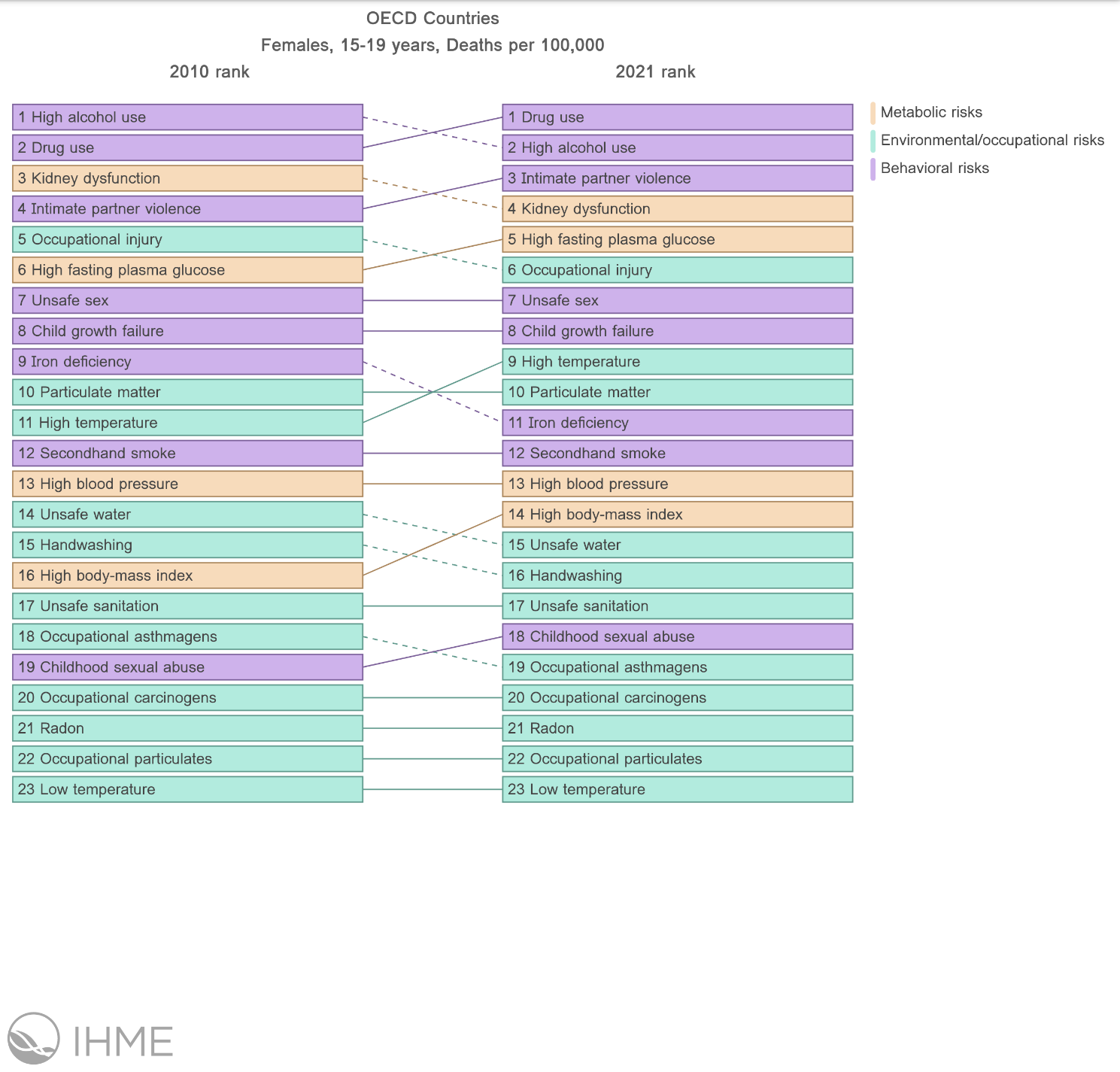


1. 20-24 years


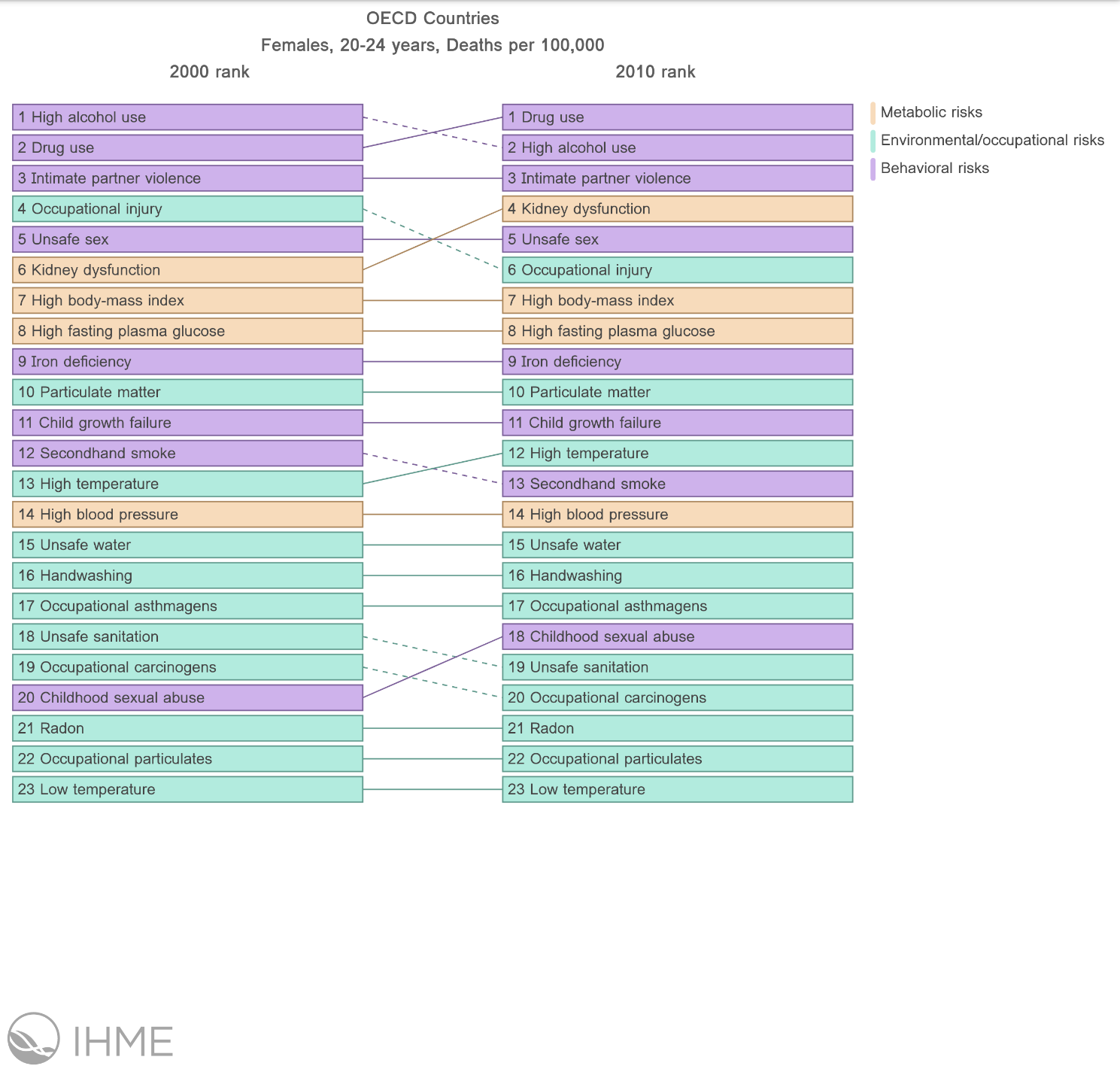

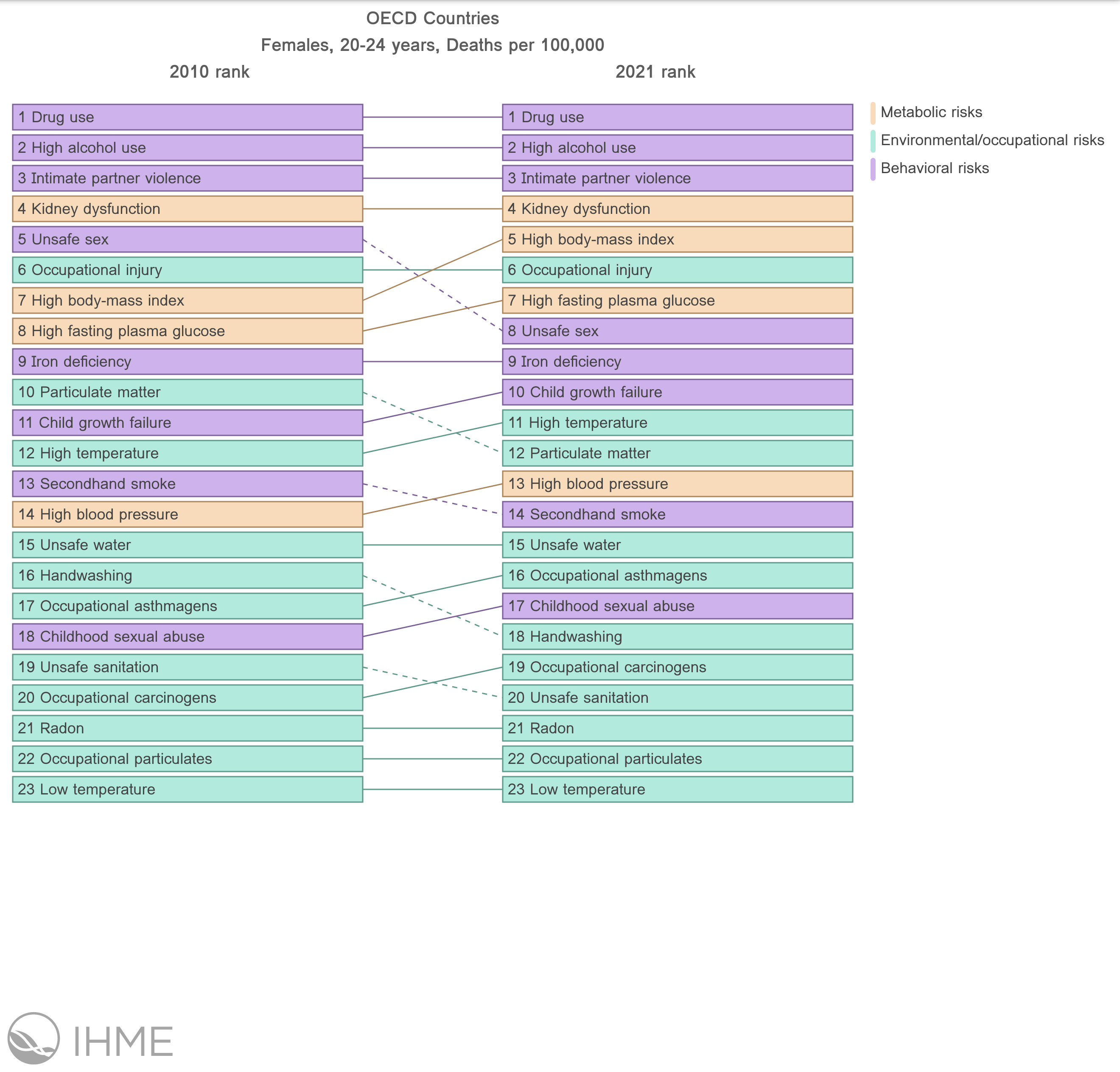


#### Figure S6: Top 15 risk factors of death for female adolescents and young adults (10-24 years) in Australia in the years 2000, 2010, and 2021

1. 10-24 years


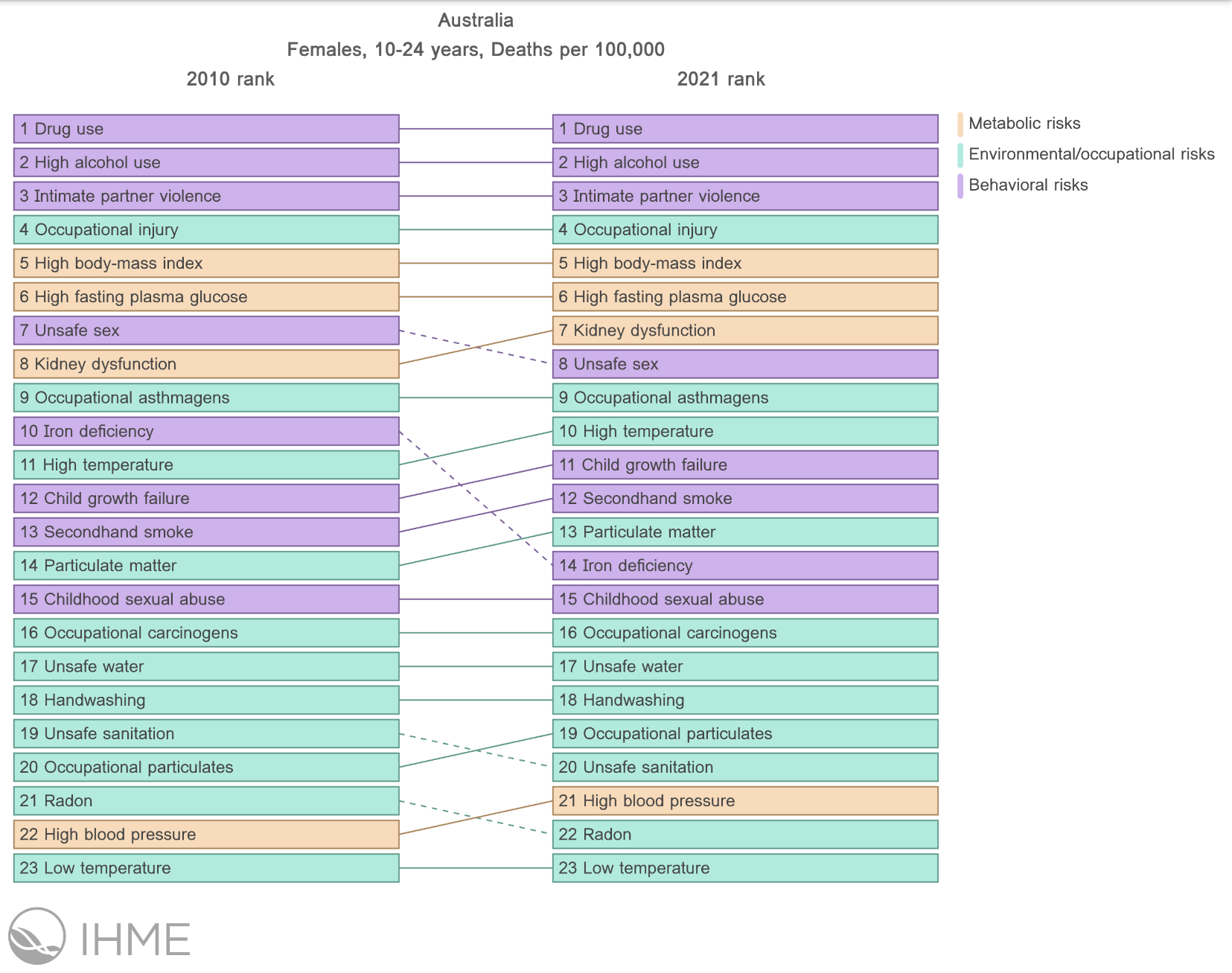

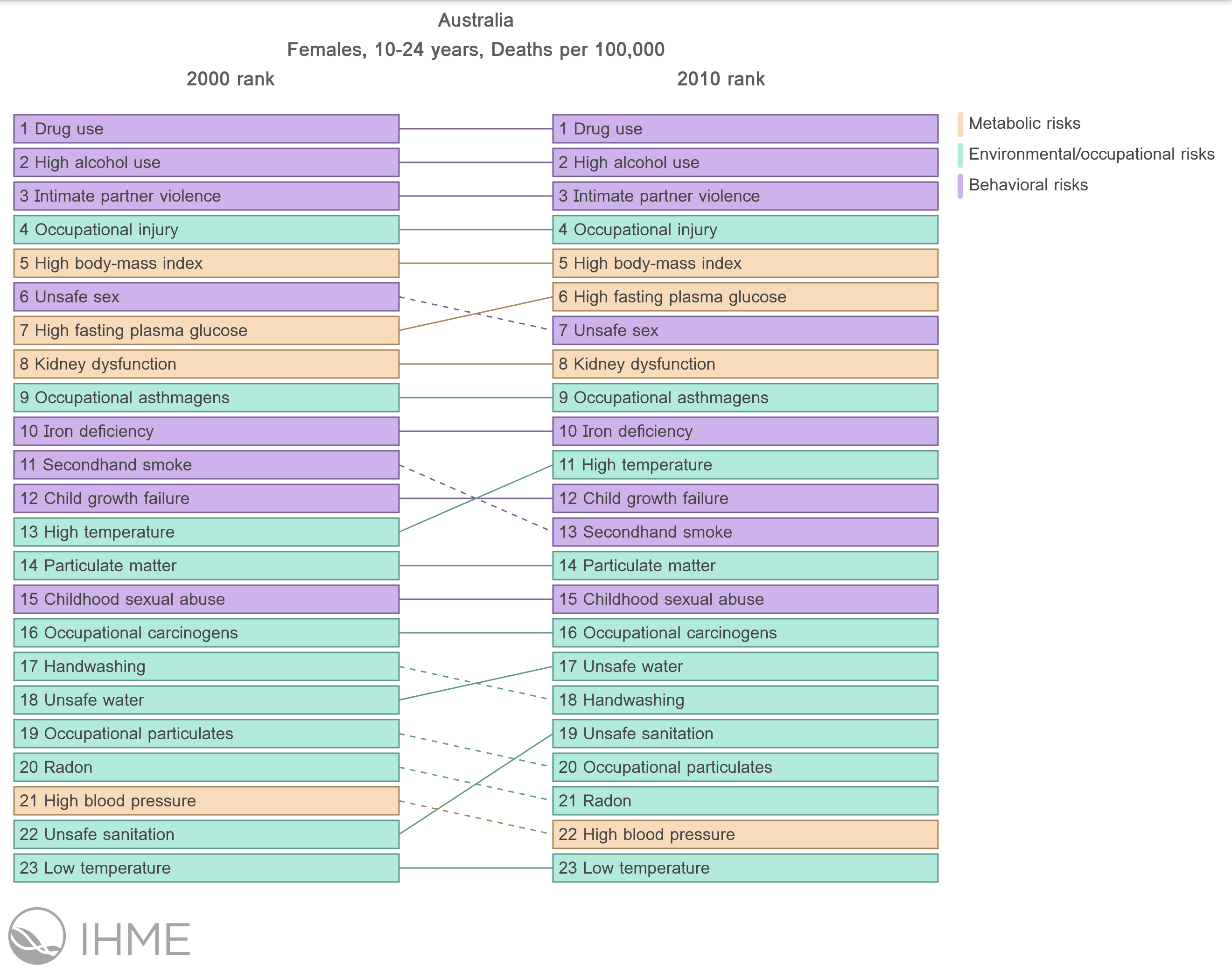


1. 10- 14 years


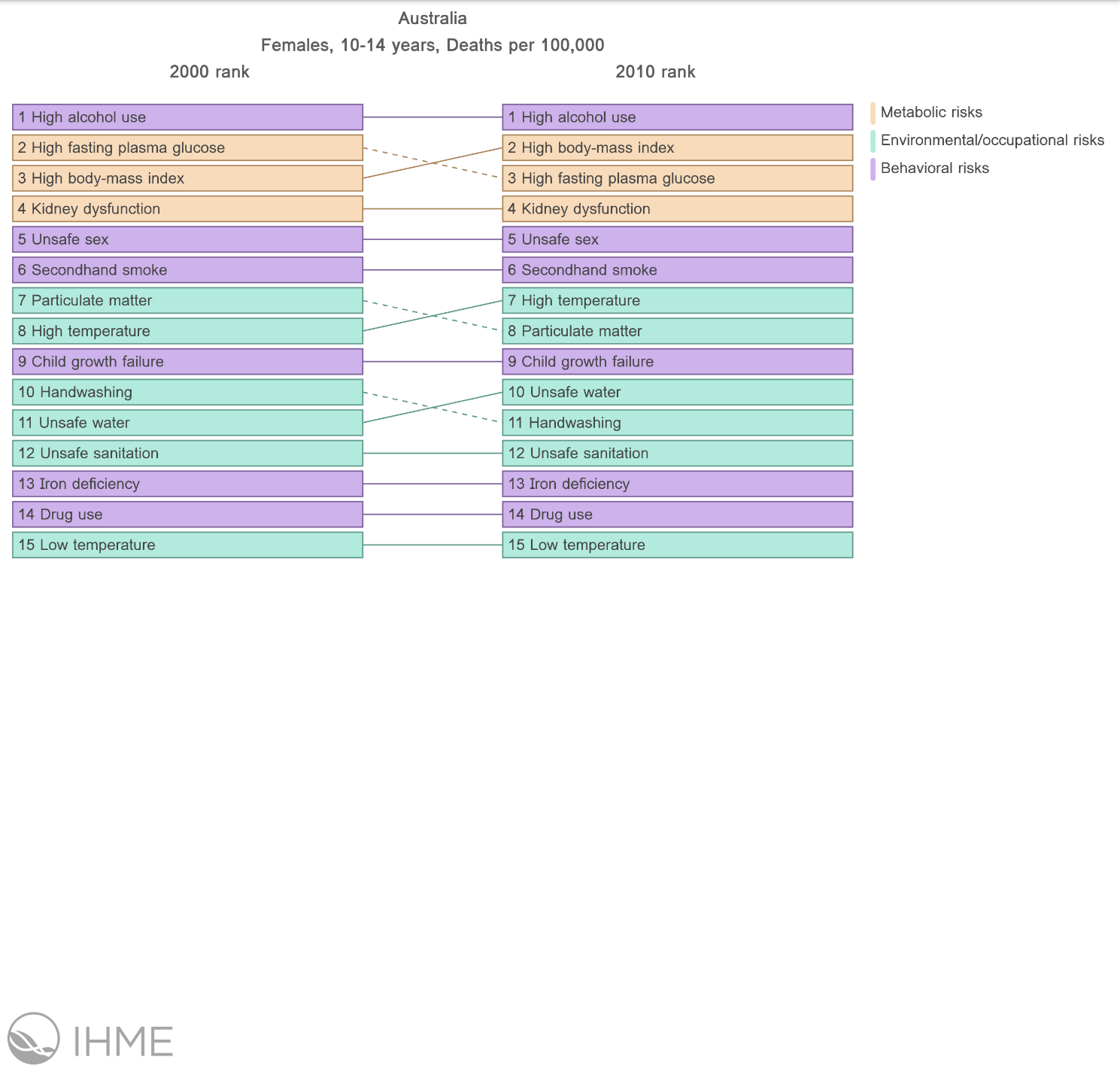

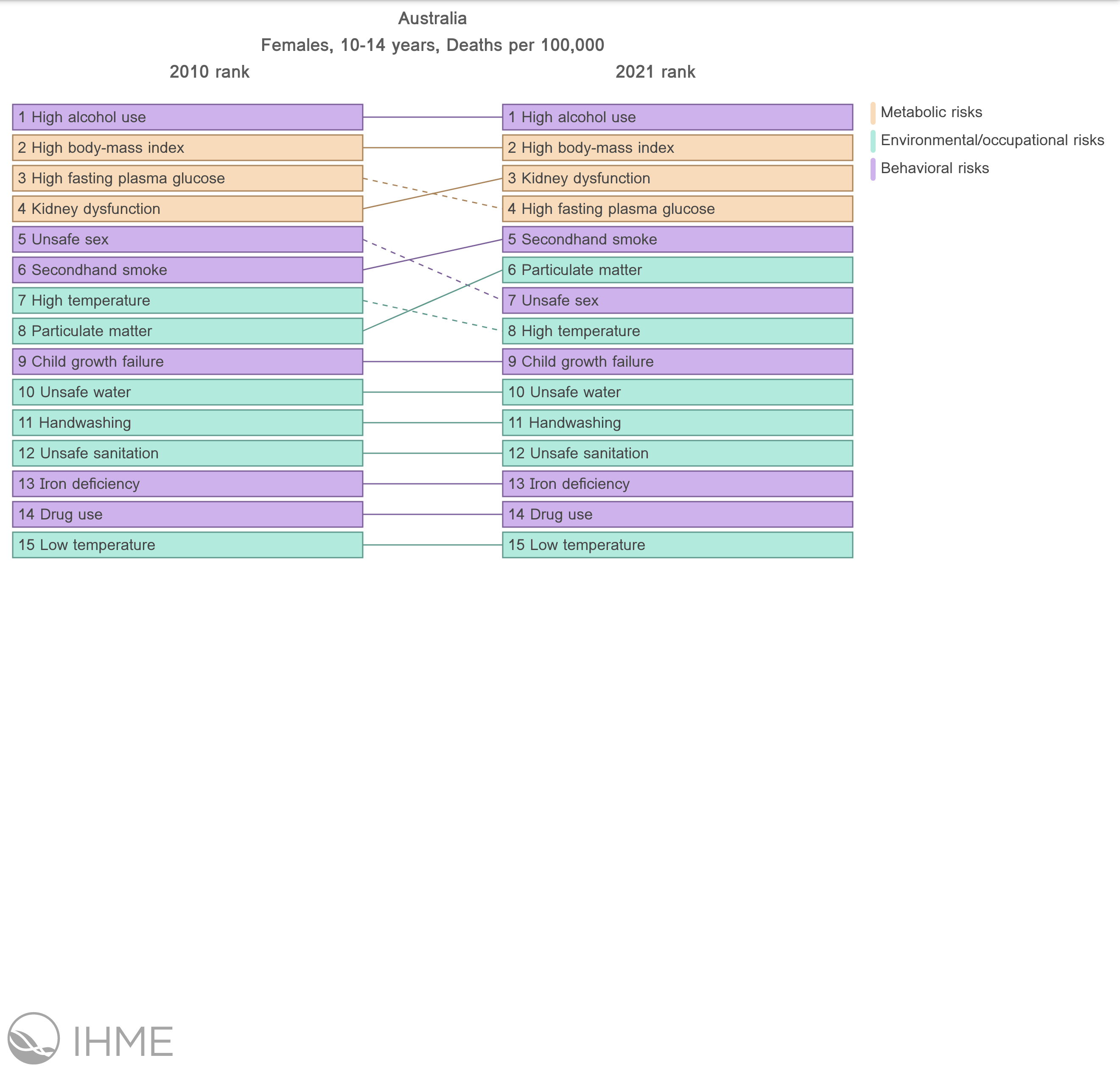


1. 15-19 years


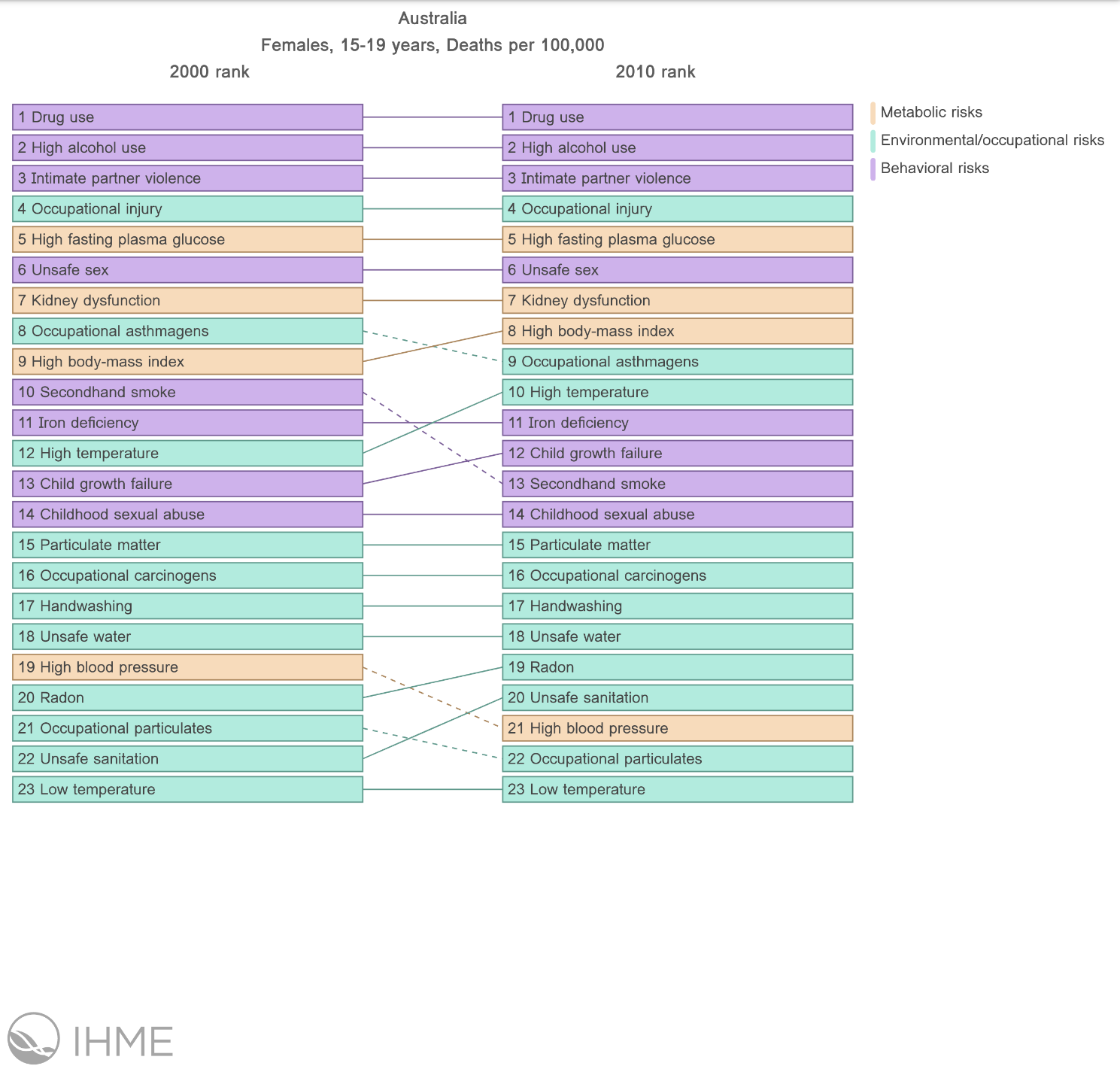

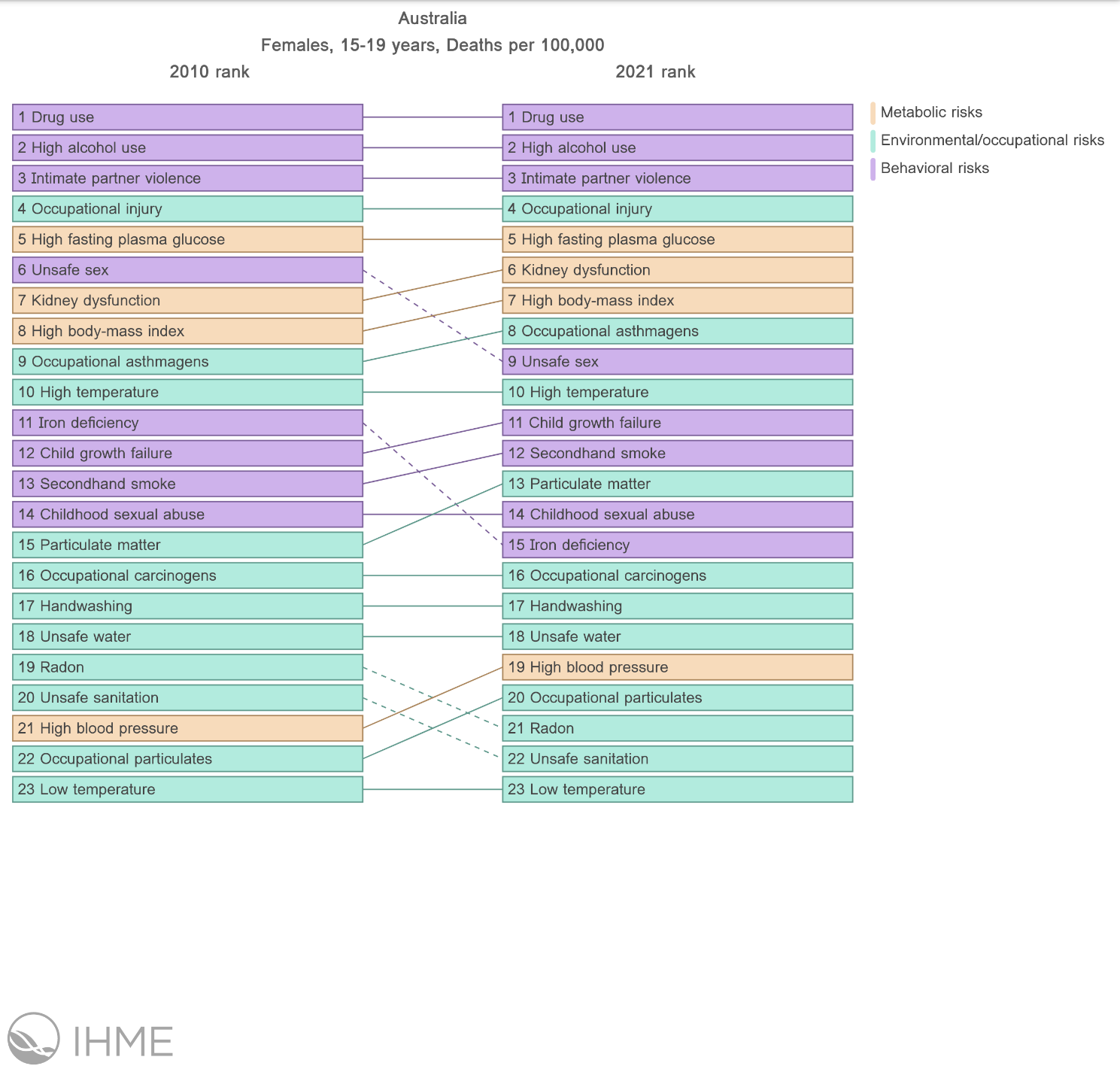


1. 20-24 years


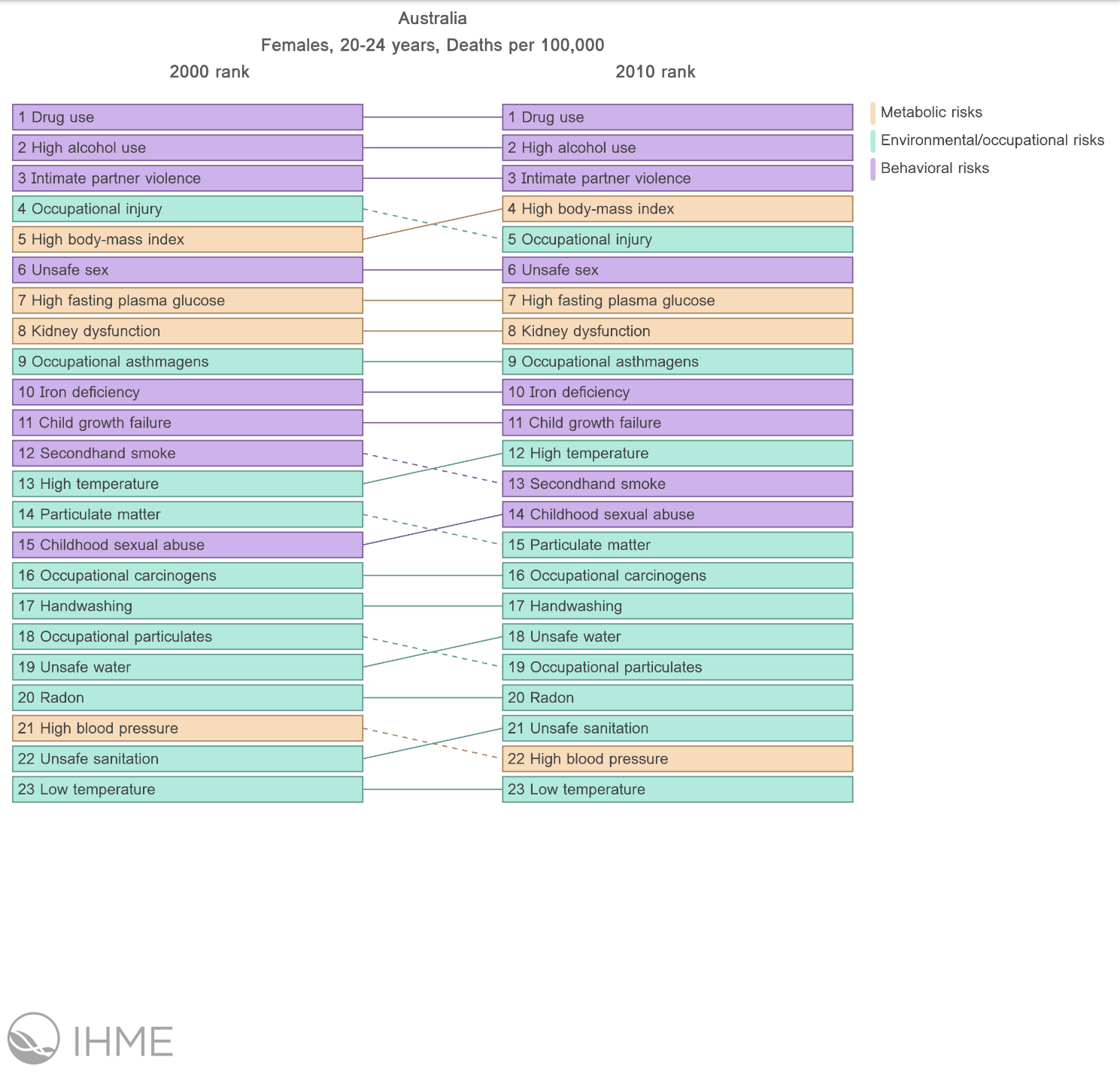

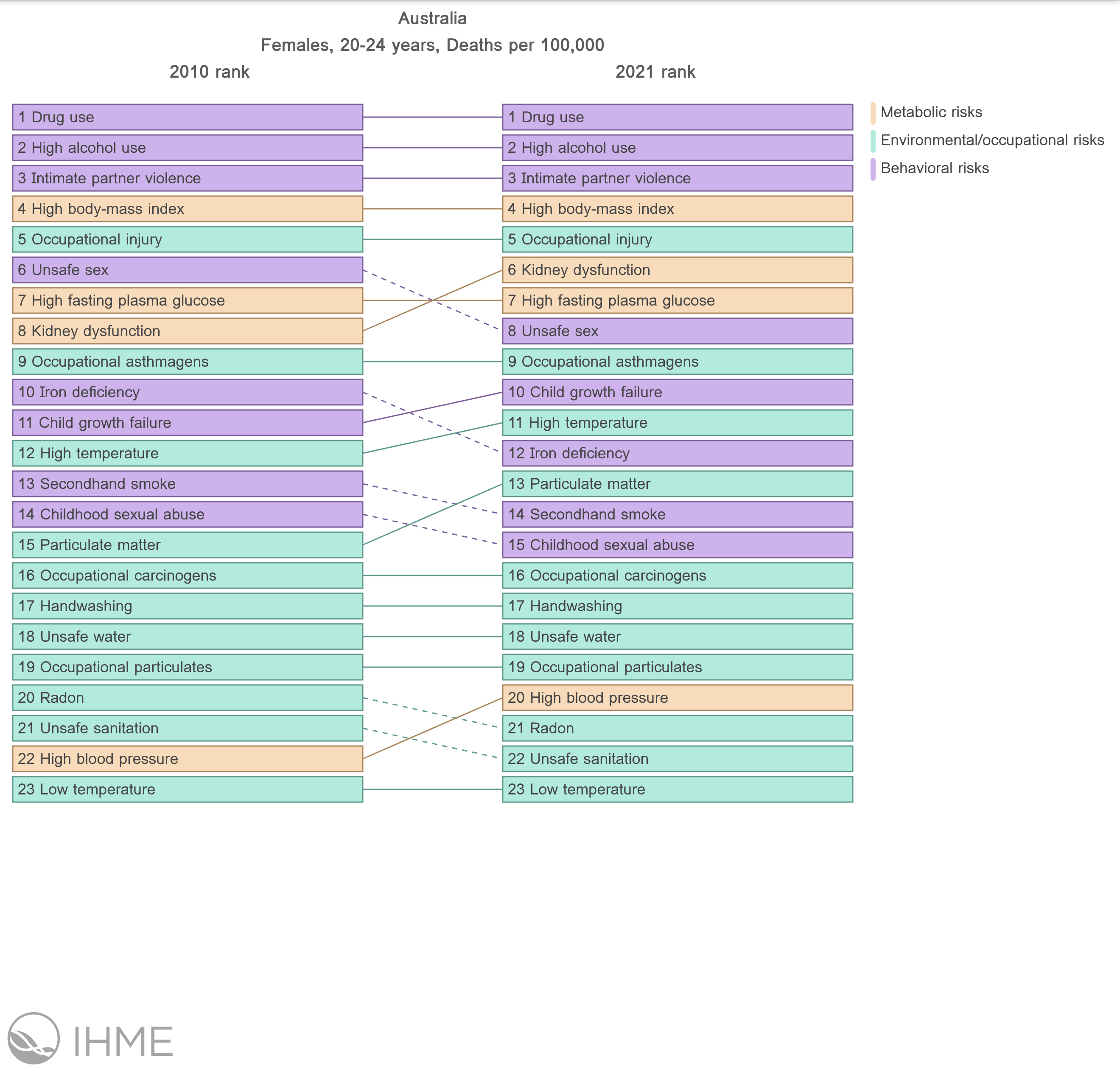

Supplement: online supplemental file 1 [file bmjph-3-2-s001.docx]
